# Design and Development of COX-II Inhibitors: Current Scenario and Future Perspective

**DOI:** 10.1021/acsomega.3c00692

**Published:** 2023-05-09

**Authors:** Sandhya Chahal, Payal Rani, Kiran, Jayant Sindhu, Gaurav Joshi, Aravindhan Ganesan, Subha Kalyaanamoorthy, Mayank, Parvin Kumar, Rajvir Singh, Arvind Negi

**Affiliations:** † Department of Chemistry, COBS&H, CCS Haryana Agricultural University, Hisar 125004, India; ‡ Department of Pharmaceutical Sciences, Hemvati Nandan Bahuguna Garhwal (A Central) University, Chauras Campus, Tehri Garhwal, Uttarakhand 249161, India; § Adjunct Faculty at Department of Biotechnology, Graphic Era (Deemed to be) University, 566/6, Bell Road, Clement Town, Dehradun, Uttarakhand 248002, India; ⊥ ArGan’sLab, School of Pharmacy, University of Waterloo, Waterloo, Ontario N2G 1C5, Canada; ¶ Department of Chemistry, University of Waterloo, Waterloo, Ontario N2L 3G1, Canada; # University College of Pharmacy, Guru Kashi University, Talwandi Sabo, Punjab 151302, India; ∥ Department of Chemistry, Kurukshetra University, Kurukshetra 136119, India; □ Department of Bioproducts and Biosystems, School of Chemical Engineering, Aalto University, Espoo 02150, Finland

## Abstract

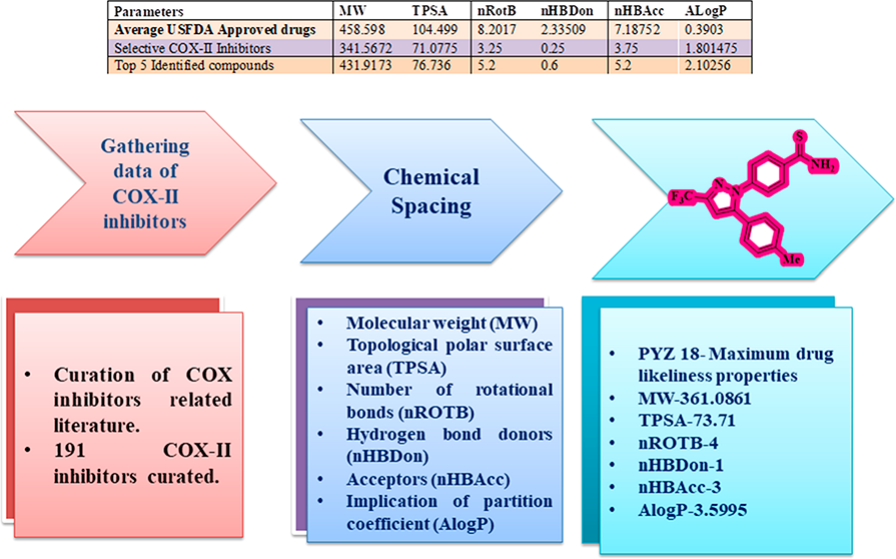

Innate inflammation beyond a threshold is a significant problem involved in cardiovascular diseases, cancer, and many other chronic conditions. Cyclooxygenase (COX) enzymes are key inflammatory markers as they catalyze prostaglandins production and are crucial for inflammation processes. While COX-I is constitutively expressed and is generally involved in “housekeeping” roles, the expression of the COX-II isoform is induced by the stimulation of different inflammatory cytokines and also promotes the further generation of pro-inflammatory cytokines and chemokines, which affect the prognosis of various diseases. Hence, COX-II is considered an important therapeutic target for drug development against inflammation-related illnesses. Several selective COX-II inhibitors with safe gastric safety profiles features that do not cause gastrointestinal complications associated with classic anti-inflammatory drugs have been developed. Nevertheless, there is mounting evidence of cardiovascular side effects from COX-II inhibitors that resulted in the withdrawal of market-approved anti-COX-II drugs. This necessitates the development of COX-II inhibitors that not only exhibit inhibit potency but also are free of side effects. Probing the scaffold diversity of known inhibitors is vital to achieving this goal. A systematic review and discussion on the scaffold diversity of COX inhibitors are still limited. To address this gap, herein we present an overview of chemical structures and inhibitory activity of different scaffolds of known COX-II inhibitors. The insights from this article could be helpful in seeding the development of next-generation COX-II inhibitors.

## 1 Introduction

Inflammation is a natural defensive reaction of the body against many types of intrinsic and extrinsic stimuli. Prolonged inflammation is often related to pathogenesis and progression of cancer,^1^ arthritis,^2^ autoimmune,^3^ cardio-vascular,^4^ and neurological disorders. Cyclooxygenase (COX) is important for triggering multiple inflammatory signaling. The overproduction of intermediates of the arachidonic acid (AA) cascade by COX is liable to produce inflammatory diseases in humans.^5^ The two main isoforms of the COX include COX-I and COX-II.^6^ COX-I is constitutive and is critical for the synthesis of prostaglandins and related entities. It is helpful in the regulation of platelet activity and gastric and renal functions. However, COX-II enzyme is induced in response to the inflammatory stimuli and has a pathological impact on living beings. It is not expressed in normal physiological states inside the human body and is typically generated in cells under pathological conditions. The uncontrolled inflammatory condition is thus related to COX-II and is responsible for the disease-related inflammatory reactions.^7^

**Figure 1:**
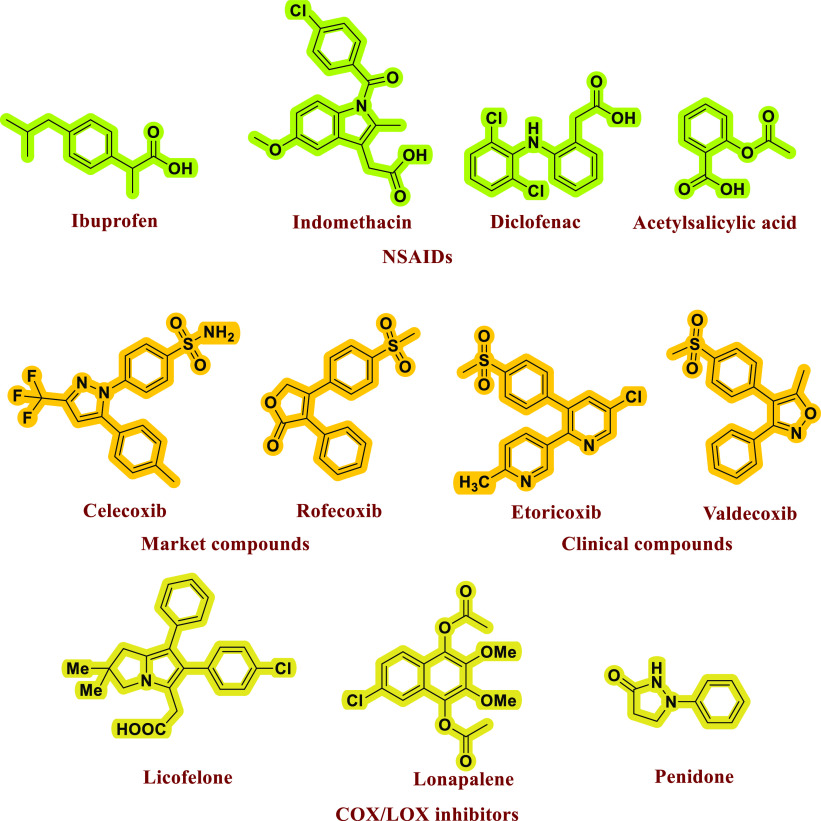
Some representative examples of COX-II inhibitors.

Considering the above facts and to overcome unwanted inflammation, several COX inhibitors have been developed, and few of them are also being utilized for treatment. The first-generation COX inhibitors include nonsteroidal anti-inflammatory drugs (NSAIDs) such as indomethacin and diclofenac, which inhibit both COX-I and COX-II enzymes. However, due to this nonselective targeting of COX-I, these agents trigger undesirable side effects of peptide ulcers and stomach bleeding to name a few (Figure 1).^8−11^ Therefore, to overcome the concerns of side effects of the nonselective COX inhibitors, COX-II selective anti-inflammatory agents (COXIBs) have, therefore, been developed. Some of the typical examples of these second-generation NSAIDs include Celecoxib, Valdecoxib, and Rofecoxib. These drugs have high selectivity indices toward COX-II inhibition over COX-I.^12,13^ The most common structural feature of COXIBs is as shown in Figure 1.

Therefore, the therapeutic benefits of COX-II selective inhibitors in the development of safer anti-inflammatory agents have been widely acknowledged. Nevertheless, many of the market-approved selective COX-II inhibitors like Rofecoxib have been removed from the market because of the cardiovascular side effects associated with these molecules. Since these COX-II selective compounds are known to inhibit many biosynthesis processes, they are prone to negatively impact the naturally balanced biochemical pathways, and, as a result, lead to high blood pressure and myocardial infection.^14^ In this context, some new lead compounds were designed that have a six-membered ring or bicyclic heterocyclic core with increased safety profiles.^15,16^

This review presents an overview of remarkable advances in the area of COX-II inhibitor development. Initially, we briefly describe the physiological and pathophysiological roles of COX, along with the structural insights of these enzymes gained from the previously reported X-ray crystal structures. Subsequently, we present an in-depth summary of different classes of known COX inhibitors and their inhibitory activities. Finally, we perform a chemical spacing analysis to identify the best-known inhibitors with maximal drug likeness properties and the possible reasons for their safety issues. This review will serve as a useful resource to understand the structure–activity relationship for wide scaffolds of COX inhibitors and, therefore, will seed the development of next generation of safe drugs for inflammatory conditions.

## 2 Physiological and Pathophysiological Roles of COX

The COX enzymes are critical for synthesizing prostanoids, as they are involved in the first step of the proteinoid biosynthetic pathway. From the early 1990s, two isoforms of COX, COX-I and COX-II, have been identified and their individual physiological and pathological roles have also been well characterized. It is known that COX-I, i.e., the housekeeper isoform, is involved in the homeostasis phenomenon and extensively controls the mucosa protection, platelet aggregation, and renal blood flow-related processes. COX-II, on the other hand, is related to pathology and adaptation processes.^17^ The most important pathological conditions that are related to COX-II expression are inflammation, pain, fever, CNS ischemia, Alzheimer, cancer, and related conditions. Further, COX-II was also found to be associated with several adaptation-related processes. The most important adaptive responses of COX-II protein include renin secretion from the kidney, wound/ulcer healing, female reproductive system, bone metabolism, and vascular protection related activities. Therefore, the role of COX-II under pathological condition is quite clear and that is also evident from many studies.^17^

**Figure 2:**
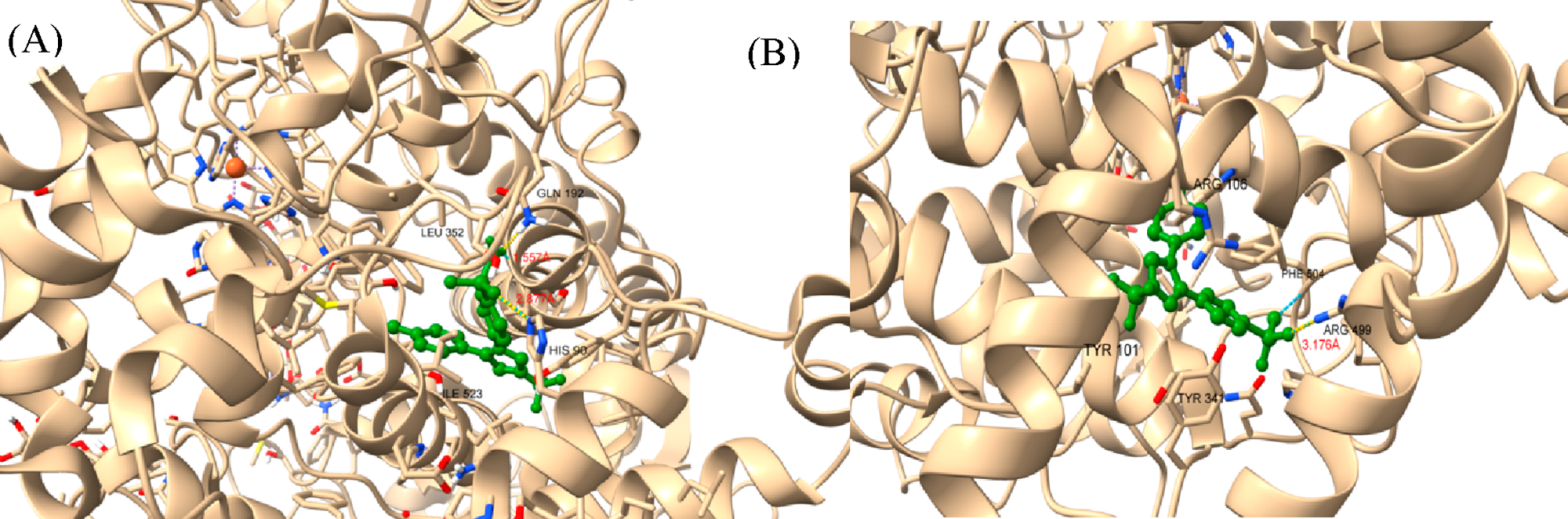
Active pockets for drug binding of (A) COX-I and (B) COX-II.

The inflammation can increase the COX-II-dependent synthesis of prostaglandin, making the peripheral nociceptors hypersensitive toward pain-related sensations. Apart from that, the central role of COX-II toward painful sensation is also evidenced by multiple studies. It has been established that prostaglandins within the CNS tend to sensitize the central nervous system to induce the hyperalgesia-related phenomenon. It has been further observed that COX-II was found overexpressed in the dorsal horn of the spinal cord under trauma-related conditions. Similarly, there are epidemiological evidences describing the preventative role of COX-II-targeted NSAIDs in Alzheimer’s disease-related conditions.^17^ For example, previous studies have shown the overexpression of COX-II within the hippocampus, cortex, and related regions in the brains of patients with Alzheimer’s disease. Moreover, upregulation of COX-II in Alzheimer’s disease is quite common, which also correlates with amyloid protein deposition within the neuritic plaques.^17^ Based on all these observations, the pathological role of COX-II is quite evident, and selective COX-II inhibitors seem highly beneficial under diverse types of disease conditions (Figure 2).

## 3 Brief Overview of the Structures of COX

**Figure 3:**
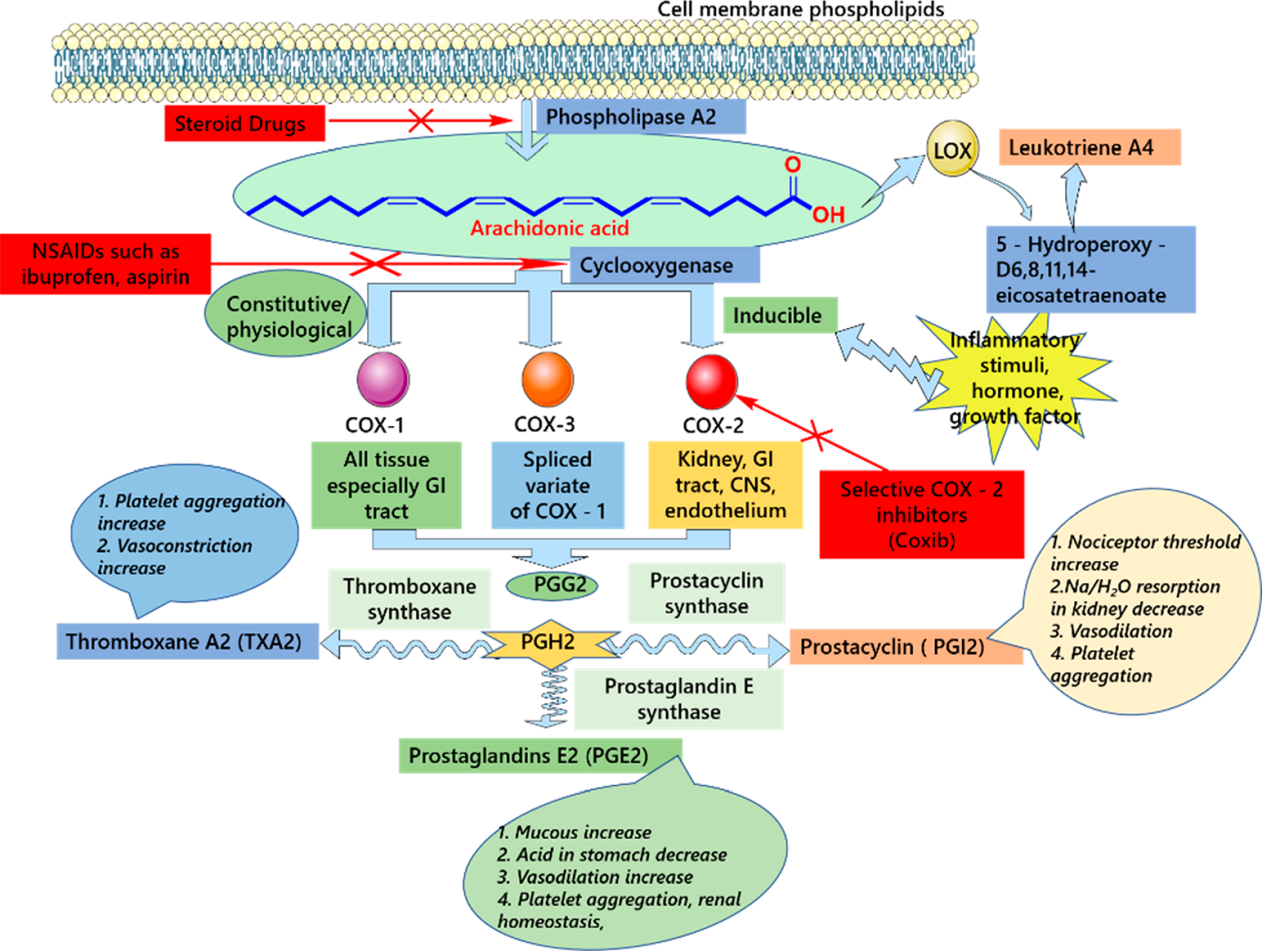
Biochemical pathway of COX inhibitors

COX enzymes are membrane-bound enzymes located in the endoplasmic reticulum and lumen of the nuclear envelope.^18^ Both COX-I and COX-II share a high level of sequence conservation (>60% of identify) and structural homology as evidenced by the reported X-ray crystal structures (PDB ID 6Y3C and 5KIR).^19−21^ Structurally, COX enzymes exist as homodimers of ∼70 kDa, and each monomer of the enzymes encompasses three domains namely the N-terminal epidermal growth factor (EGF) domain (residue 34 to 72), a membrane-binding domain (residue 73–116), and the bulky globular C-terminal domain that is involved in the enzyme catalysis.^22^ The activation of cyclooxygenase requires catalysis of hydroperoxide, which oxidizes the prosthetic group found at the peroxidase active site to an oxo-ferryl heme complex. Then the oxidized heme molecule abstracts an electron from Tyr-385 found in the active site of COXs and results in abstraction of 13-pro-S-hydrogen from arachidonic acid and starts the cyclooxygenases reaction.^23,24^ Therefore, COX enzymes contain three distinct binding sites for binding COX substrate, peroxidase (POX), and the heme prosthetic group, which are structurally correlated to support the catalytic role of the enzymes.^25^ The binding sites of COX and POX are located at the opposite faces of the C-terminal catalytic domain, while the heme is located within a narrow pocket at the base of the POX^20,26,27^ site. The membrane binding domains of COX enzymes are made up of four alpha helices that help in linking the enzymes to the lipid bilayer of the cell membrane.^23^ The active site of COX enzymes extends from an “*L-shaped*” tunnel emerging from the membrane binding domain.^27^ The bottom end of this tunnel, closer to the membrane biding domain, presents a large surface area that is commonly known as the “*lobby*” region. This lobby site is interconnected to the COX active site via a slender hydrophobic path. It has been proposed that the substrate or the inhibitor had to first access the lobby region and subsequently pass through the narrow constriction before binding into the active site in COX (Figure 3).

The surface residues of the catalytic domain and the EGF domain are involved in the formation and stabilization of the dimer interface in COX-enzymes. The sequences, structures, and the active sites of the two COX isoforms exhibit a high level of similarity; they do also exhibit some important differences. For example, COX-II has been identified to possess a unique lateral pocket formed my residues such as Val523 and Arg513, which have been substituted as Ile523 and His513 in COX-I. Such amino acid differences^28,29^ among the two COX isoforms have also shown to shape their solvent accessible surface area (SASA) properties differently: COX-II exhibits a larger SASA than that seen in COX-I. In addition, Vane et al.^30^ have also summarized some notable disparities in the selectivity of molecules (substrates and inhibitors) binding to the two COX isoforms. COX-2 can oxygenate a wide range of fatty acid substrates (e.g., linolenic acids and eicosapentaenoic acid) more effectively than COX-I enzyme. The differences between the two COX isoforms observed at molecular level have been major factors in designing selective inhibitors.^31^ Owing to physiological and pharmaceutical significance of COX enzymes, there has been ample work in the literature that describes their structures and interactions with substrates and inhibitors, including in-depth review articles in these aspects.^32−34^ The following sections of the article will mainly focus on the detailed discussion on different classes of COX-inhibitors to understand their structure–activity relationships.

## 4 Recently Reported COX Inhibitors

Due to the therapeutic benefits of inhibiting COX enzymes in various inflammatory-related conditions, extensive efforts went into the development of diverse classes of COX inhibitors in past decades. A wide variety of chemical scaffolds have been exploited to synthesize heterocyclic COX inhibitors and these include derivatives based on pyrazoles, isoxazole, thiazole, benzoxazole, coumarin, quinoline, and pyridine, to name a few. In addition, several inhibitors have been proposed through molecular hybridization, a rational design approach that combines pharmacophore fragments from multiple compounds, and through extraction from natural sources. These inhibitors exhibit a broad range of activity profiles and selectivity against the two COX enzymes. The following section will review inhibitors within different chemical scaffolds, their chemical structures, and inhibitory activity from the literature. We will attempt to provide some basis on their structure–activity relationships and the possible mode-of-action against the COX enzymes. These resources should be helpful to understand the current state of COX-inhibitors and guide the future development of safe and selective inhibitors.

### 4.1 Pyrazole Derivatives as Anti-inflammatory Agents

**Figure 4:**
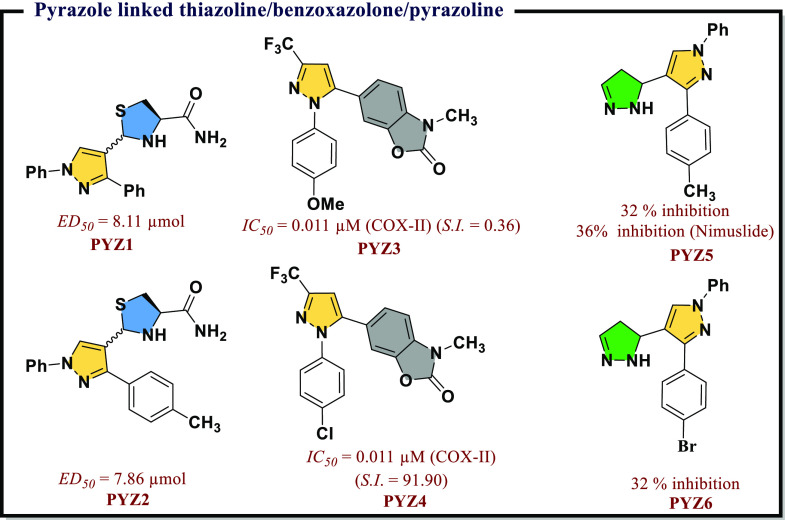
Pyrazole linked thiazoline/benzoxazolone/pyrazoline as potential COX-II inhibitors.

Pyrazole fragment have received huge attention because of anti-inflammatory, anticancer, antifungal, and other biological properties. Celecoxib and antipyrine are two established drugs from this class with promising anti-inflammatory and analgesic properties. After Celecoxib, pyrazole has received considerable attention for the development of safer anti-inflammatory molecules.^35^ Most of the pyrazole derivatives are reported to show COX-II selectivity and anti-inflammatory abilities. In a report by Bekhit et al.,^36^ a series of 4-thiozolylpyrazolyl derivatives was developed, showing *in vitro* COX potential and anti-inflammatory potency *in vivo*. Herein, the two compounds, **PYZ1** and **PYZ2**, exhibited COX-II selectivity and minimal ulcerogenic effects (Figure 4).

**Figure 5:**
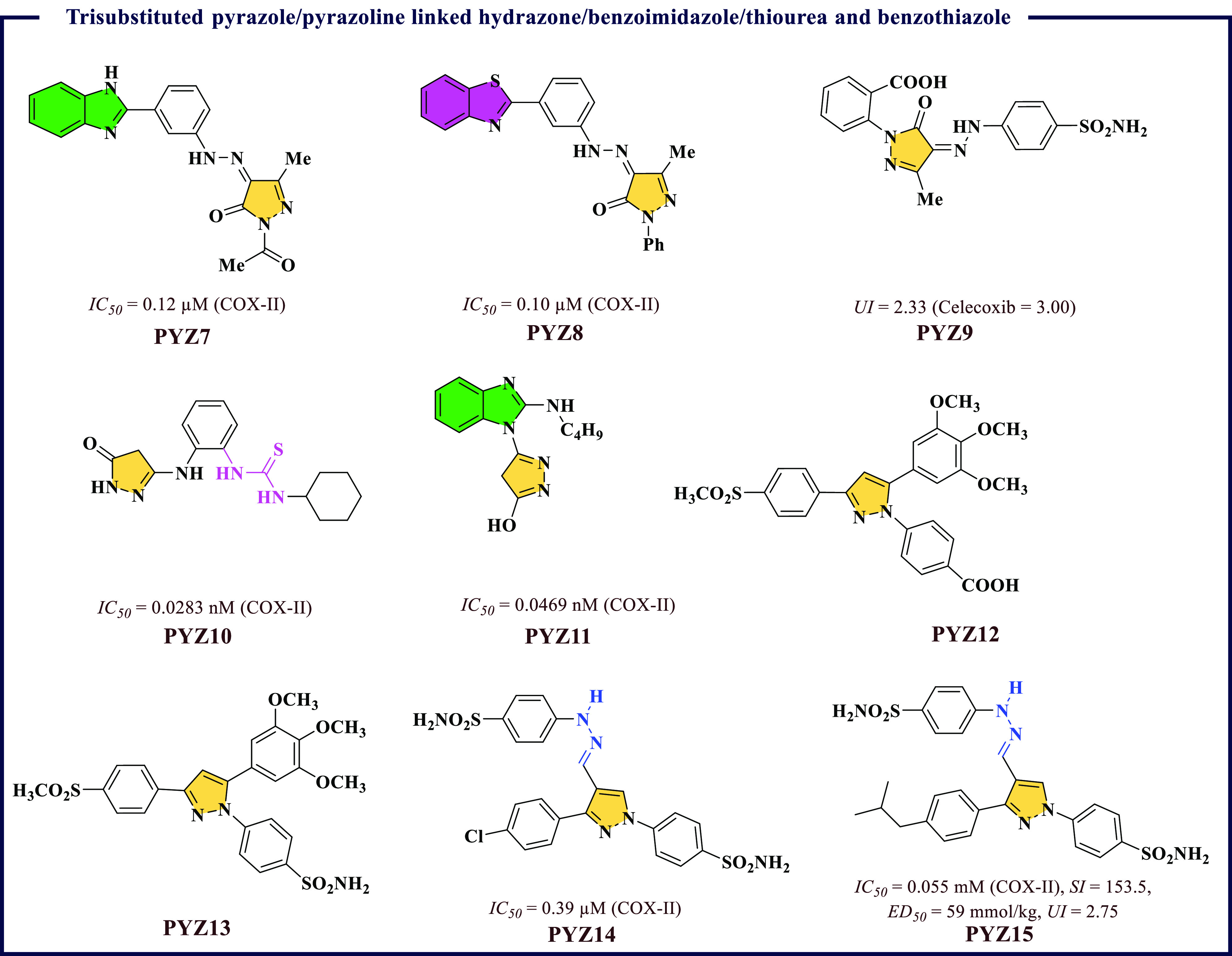
Trisubstituted pyrazole/pyrazoline linked hydrazone/benzoimidazole/thiourea and benzothiazole as potential COX-II inhibitors.

Series by Eren et al.^37^ that include diaryl heterocycles derivatives with 2-oxo-5*H*-furan, 2-oxo-3*H*-1,3-oxazole, and 1*H*-pyrazole as main scaffolds have been reported (Figure 4). Among all the molecules, **PYZ3** was most potent (*IC*_*50*_ = 0.011 μM) against COX-II protein. It showed 38-times more potency than Rofecoxib, but it was not selective against COX-II protein. However, **PYZ4**, a modified version of **PYZ3**, revealed manifold increase in the selectivity toward COX-II protein. Sharma et al.^38^ also reported a similar series that include pyrazolyl pyrazolines-based moieties. Two molecules, **PYZ5** and **PYZ6**, showed excellent anti-inflammatory potential (Figure 4). Abdelgawad et al.^39^ reported a novel series of benzo-pyrazole-based hybrid molecules. The reported compounds (Range of *IC*_*50*_ = 0.10–0.27 μM) were found better than Celecoxib (*IC*_*50*_ = 1.11 μM) for COX-II protein; **PYZ8** was found to be most potent and maximum COX-II selectivity was achieved by **PYZ7** molecule (Figure 5). Similarly, a series of analgesic molecules with 4-aryl-hydrazonopyrolones functionalities also revealed the anti-inflammatory potential and *in vitro* COX-II/5-LOX inhibitory action.^40^ Most of the compounds were found to be good inhibitors of COX-II (*IC*_*50*_ = 0.66–2.04 μM) and 5-LOX (*IC*_*50*_ = 0.52–1.59 μM) in comparison to Celecoxib (*IC*_*50*_ = 0.89 μM) and Zileuton (*IC*_*50*_ = 0.77 μM), respectively. Compound **PYZ9** (*U.I.* = 2.33) exhibited maximum activity with *IC*_*50*_ value of 0.72 μM along with better gastric profile than Celecoxib (*UI* = 3.00) (Figure 5).

**Figure 6:**
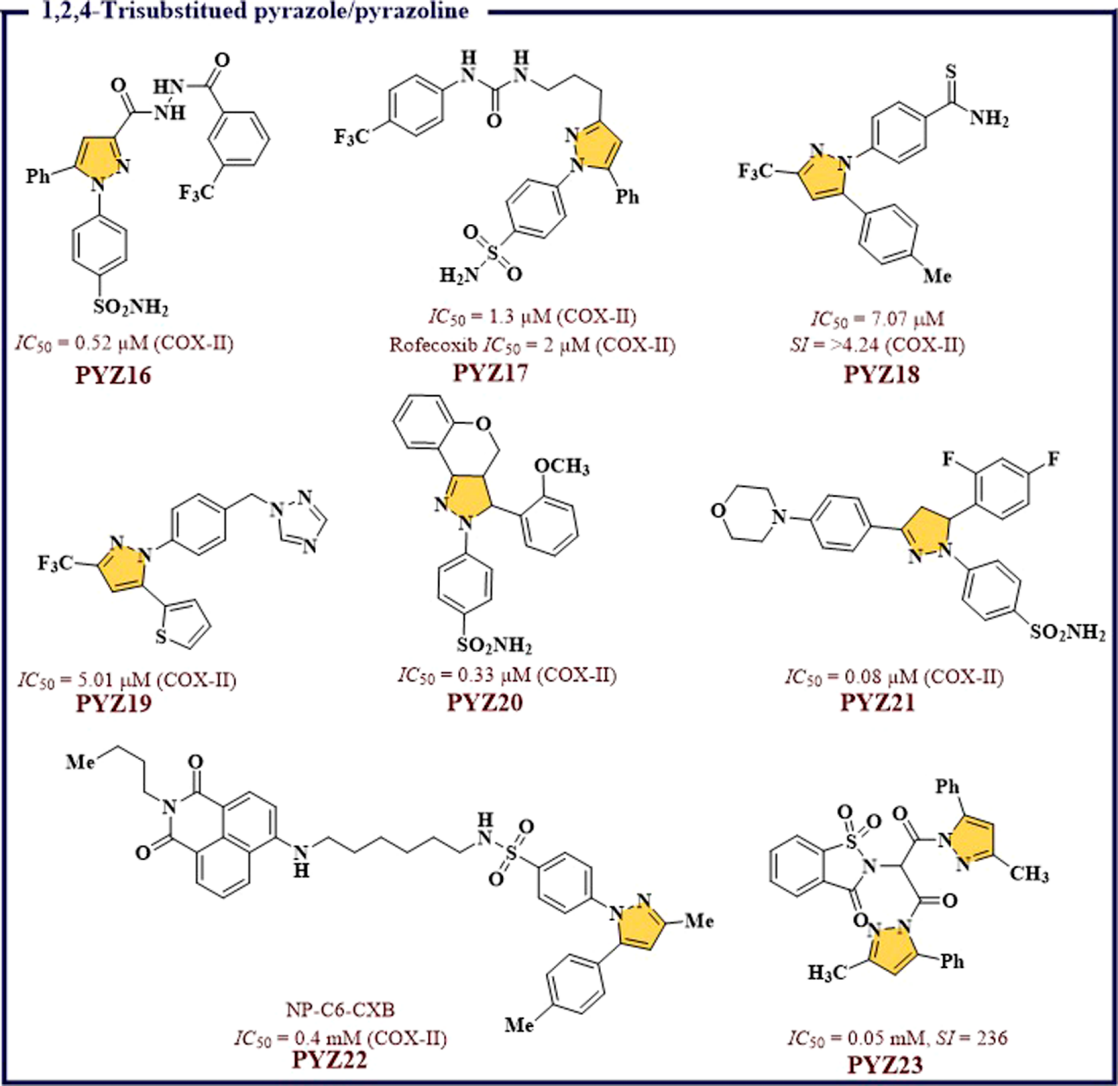
1,2,4-Trisubstituted pyrazole/pyrazoline as potential COX-II inhibitors.

A series of pyrazole-thiourea-benzimidazole based hybrid molecules was successfully designed by Moneer et al.,^41^ and all the molecules were found selective toward COX-II protein. The most promising compounds (Figure 5), **PYZ10** and **PYZ11**, were identified to exhibit COX-II inhibition with *IC*_*50*_ values of 0.0283 nM and 0.2272 nM, respectively. A similar report by Abdellatif et al.^42^ had reported a series of triarylpyrazoline derivatives with carboxy and sulphonyl groups. Among all the reported molecules, **PYZ12** and **PYZ13**, with trimethoxyphenyl-based structural feature, showed maximum potency against COX-II protein (Figure 5). Continuing their efforts in this direction, Abdellatif et al.^43^ further reported a series of 1,3,4-trisubstituted-pyrazole derivatives and many of these were found better then Celecoxib for COX-II, and **PYZ14** was found the best molecule (Figure 5). Abdellatif further developed a series of molecules with diarylpyrazoles- and triarylpyrazoles-based functionalities.^44^ These molecules were found more selective toward COX-II in comparison to COX-I, and **PYZ15** was found to be the best molecule. Pavase et al.^45^ designed novel sulfonamides having diarylpyrazoles by computational studies. Best computational hits were synthesized and assessed for their *in vitro* activity against COX-I/II. Weak inhibitory activity against COX-I and moderate against COX-II with *IC*_*50*_ value in the range of 0.52–22.25 μM was observed for all the compounds. Highest COX-II inhibitory and selectivity (*IC*_*50*_ = 0.52 μM (COX-II), *S.I.* = 10.73) were observed for **PYZ16** as compared to standard drug Celecoxib (*IC*_*50*_ = 0.78 μM, *S.I.* = 9.51). *In vivo* anti-inflammatory activity of **PYZ16** exhibited 64.28% inhibition in comparison to 57.14% for Celecoxib (Figure 6). Similarly, a report by Hwang et al.^46^ also revealed the role of 1,5-diarylpyrazoles-urea based hybrid molecules in COX-II inhibition. Among all, **PYZ17** was found showing good COX-II inhibitory potential (Figure 6). Chandana et al.^47^ had reported new 1,5-diaryl-based analogs of Celecoxib that exhibited anti-inflammatory (AI) activity *in vivo*. Most of the compounds exhibited more pronounced COX-II inhibition, but they also inhibited COX-I effectively with less selectivity against COX-II. Maximum potency and selectivity were observed for **PYZ18** against COX-II with an *IC*_*50*_ = 7.07 μM and *S.I.* = >4.24 (Figure 6). Chandna et al.^48^ reported a series of pyrazolylbenzyltriazoles based compounds. These compounds have exhibited moderate to strong inhibitory activity against COX-II and COX-I. Among all, **PYZ19** inhibits COX-II activity by >70% with an *IC*_*50*_ = 5.01 μM compared with 86.04% inhibition in case of reference drug, i.e., Celecoxib (Figure 6). Chen et al.^49^ synthesized and evaluated a new class of COX-I/II inhibitory dihydropyrazole sulfonamide derivatives. Among them, **PYZ20** exhibited maximum potency and selectivity against COX-II with an *IC*_*50*_ = 0.33 μM as compared to reference drug Celecoxib (*IC*_*50*_ = 0.052 μM). Qiu et al.^50^ reported a sequence of dihydropyrazole sulphonamide whereby reasonable potency against COX-II was observed for **PYZ21** with an *IC*_*50*_ value of 0.08 μM (Figure 6). Similarly, a report by Gurram et al.^51^ had shown the COX-II targeting potential of **PYZ22**-related molecules. It is a naphthylamide conjugate system, showing *IC*_*50*_ = 0.4 mM against COX-II protein. **PYZ23** is yet another COX-II selective molecule that is reported by Taher et al.^52^ The report contains a benzenesulfonamide and 1,2-benzisothiazol-3(2*H*)-one-1,1-dioxide derivatives that displayed good to moderate COX-I and II inhibition. **PYZ23** was found to be the most potent selective inhibitor of the COX-II protein.

**Figure 7:**
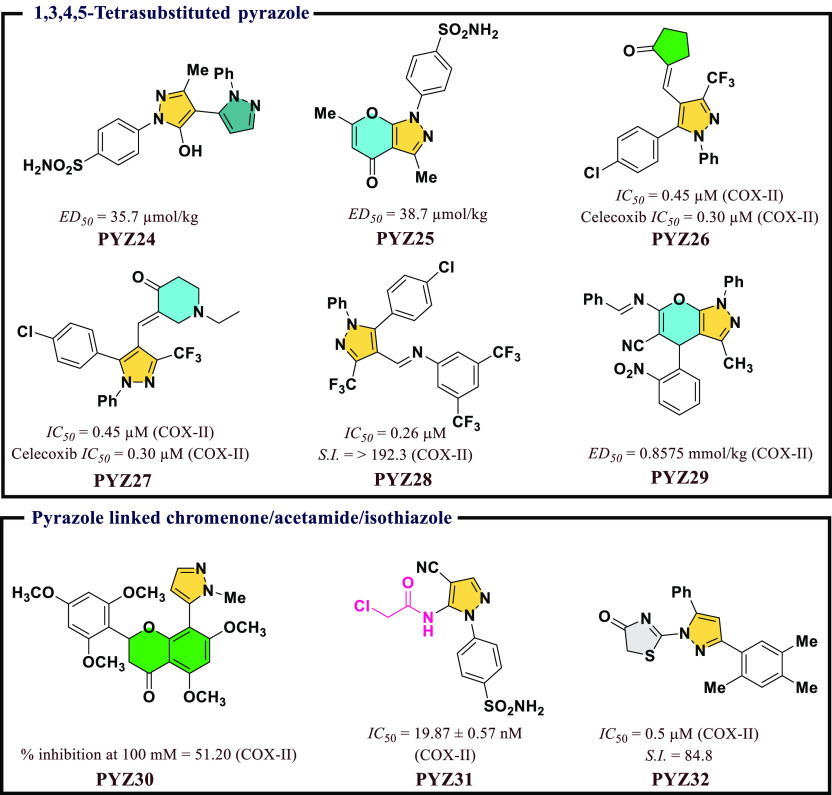
1,3,4,5-Tetrasubstituted pyrazole and pyrazole linked chromenone/acetamide/isothiazole as potential COX-II inhibitors.

Faidallah et al.^53^ reported a series of novel nonacidic poly substituted pyrazoles and pyrano[2,3-*c*]pyrazoles. The anti-inflammatory activity of the synthetic molecules was evaluated, and most of the synthesized compounds exhibited *ED*_*50*_ value of 35.7–75.2 mmol/kg. Herein, **PYZ24** and **PYZ25** (Figure 7) showed *ED*_*50*_ values of 35.7 and 38.7 μmol/kg, respectively, against COX-II inhibition and were as competent as Celecoxib (*ED*_*50*_ = 32.1 μmol/kg). A novel series of synthetic arylhydrazones and 1,5-diphenyl pyrazole with potent anti-inflammatory potencies through the inhibition of COX enzymes were reported by El-Sayed et al.^54^ Among all, **PYZ26** and **PYZ27** exhibited low micro molar range of inhibitory action against to COX-II. Similarly, El-Sayed and co-workers^55^ also developed a series of new pyrazole and pyrazoline derivatives to inhibit ovine COX-I/II isozymes. Among all, **PYZ28** exhibited optimum COX-II inhibitory activity (*IC*_*50*_ = 0.26 μM) and selectivity (= >192.3) comparable with standard drug Celecoxib (*IC*_*50*_ = 0.28 μM and *S.I.* = 178.57). Murahari et al.^56^ designed and synthesized a novel series of pyrazole derivatives using ligand-based approach, and among them, **PYZ29** was found to be the best molecule (Figure 7).

A series of novel pyrazole functionalized flavones was reported by Chavan et al.,^57^ and most of the compounds inhibited both COX-I and COX-II, but some of them exhibited selective inhibition for COX-II. Herein, **PYZ30** exhibited most significant inhibitory activity against COX-II (Figure 7). Hassan et al.^58^ reported a series of new pyrazole derivatives and evaluated them *in vitro* against COX-I and COX-II. *IC*_*50*_ value of 19.87 nM was observed for **PYZ31** against COX-II, which revealed the presence of better activity profile in comparison to Celecoxib (35.56 ± 1.02 nM) (Figure 7). A series of dihydro-pyrazolyl-thiazolinone derivatives was developed and evaluated for their inhibitory activity against COX-II by Qiu et al.^59^ Most of the compounds have low toxicity and excellent inhibitory potential against both COX-I and II, but some of them inhibit COX-II selectively. Among them, **PYZ32** was the most potent (*IC*_*50*_ = 0.5 μM for COX-II) in comparison to standard drug Celecoxib (*IC*_*50*_ = 0.1 μM for COX-II) (Figure 7).

**Figure 8:**
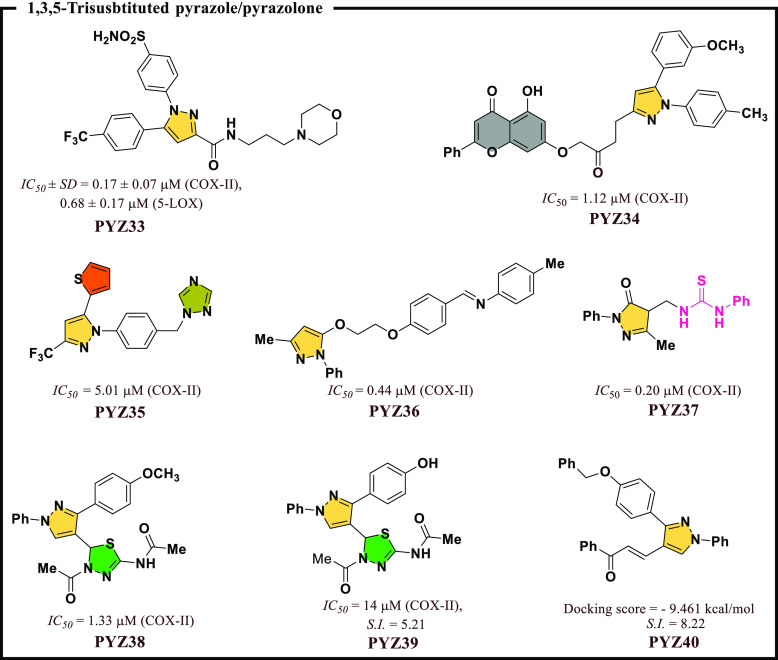
1,3,5-Trisubstituted pyrazole/pyrazolone as potential COX-II inhibitors.

**Figure 9:**
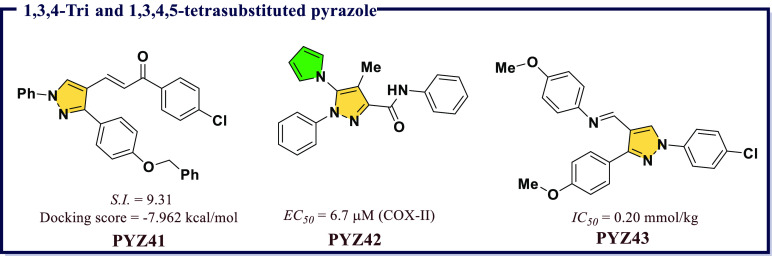
Some 1,3,4-tri and 1,3,4,5-tetrasubstituted pyrazoles as potential COX-II inhibitors.

Li et al.^60^ reported a library of compounds based on diaryl-1,5-diazoles and morpholine framework. The developed library had COX-II/5-LOX inhibitory potential, and **PYZ33** was found best among all these molecules (Figure 8). A report by Ren et al.^61^ developed a novel series of 1,5-diarylpyrazole derivatives clubbed with chrysin and explored their anti-inflammatory response. Most compounds have shown COX-II inhibitory potential, and **PYZ34** was found to be the best molecule (Figure 8). Yan et al.^62^ had designed a series of dihydropyrazole framework consisting of benzo oxygen heterocycles and sulfonamide moiety. This work resulted in **PYZ35** molecules, showing good biological potential (Figure 8). Tewari et al.^16^ also developed a series of novel pyrazole analogues with anti-COX potential. Among all the screened compounds, **PYZ36** exhibited anti-inflammatory activity along with optimum COX-II inhibitory potential (*IC*_*50*_ = 0.44 μM) (Figure 8). Dube et al.^63^ reported new pyrazolone derivatives whereby they have shortlisted eight derivatives with pronounced COX-II inhibition potential. In particular, **PYZ37** (Figure 8) had 2-fold higher inhibitor potency (*IC*_*50*_ = 0.2 μM) than Celecoxib (*IC*_*50*_ = 0.4 μM). Another study by Alegaon et al.^64^ also reported a few 1,3,4-trisubstituted pyrazole derivatives with moderate inhibitory activity against COX-II with *IC*_*50*_ ranging between 1.33 and 17.5 μM. This screening identified *N*-(4-acetyl-5-(3-(4-methoxyphenyl)-1-phenyl-1*H*-pyrazol-4-yl)-4,5-dihydro-1,3,4-thiadiazol-2-yl)acetamide **PYZ38** (Figure 8) to display effective and selective COX-II inhibitory potential (*IC*_*50*_ = 1.33 μM; *S.I.* > 60). Similarly, Alegaon and co-workers^65^ reported a few novel derivatives with potent anti-inflammatory activity whereby **PYZ39** (Figure 8) was found to show significant COX-II inhibitory potential with *IC*_*50*_ of 6.5 μM,

Harras et al.^66^ also reported novel 1,3,4-trisubstituted pyrazole derivatives with COX-I and COX-II inhibition potential. Two chalcones, **PYZ40** (Figure 8) and **PYZ41** (Figure 9), were the most selective COX-II inhibitors (*S.I.* = 8.22 and 9.31, respectively). Manvar et al.^67^ also reported a novel pyrazolecarboxamide derivative, **PYZ42**, that was found targeting the overexpressed COX-II induced under HCV conditions. A selection of experimentally validated novel 1,3,4-trisubstituted pyrazoles with potent anti-inflammatory and analgesic activities with a better GIT tolerance than phenylbutazone were reported by Ragab et al.^68^ Herein, the 4-substitution of phenyl moiety attached to pyrrole ring with an electron withdrawing fluoro group found increased activity of the compounds. **PYZ43** was identified the most active anti-inflammatory and analgesic agent with better % protection (66.67) and lower ulcer indices than phenylbutazone (Figure 9).

**Figure 10:**
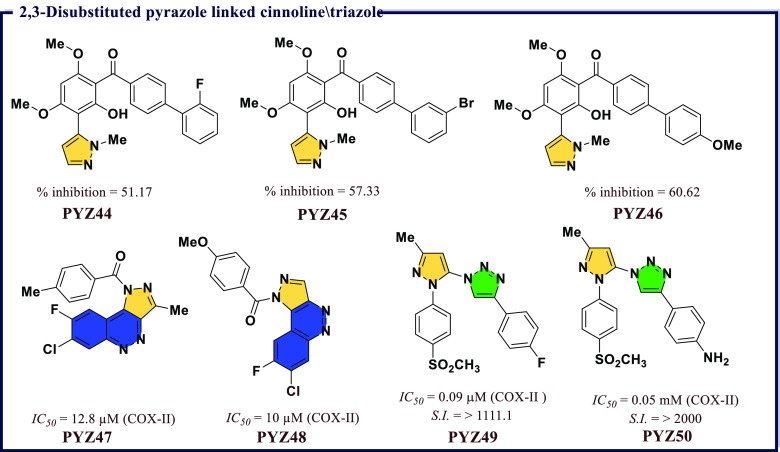
2,3-Disubstituted pyrazole linked cinnoline/triazole as potential COX-II inhibitors

Bandgar et al.^69^ reported a list of novel pyrazole integrated benzophenones that were synthesized and confirmed to show promsing anti-inflammatory activity in the cell-based carrageenan paw edema in rats and potent COX inhibition in the *in vitro* assay. The entire series of benzophenone analogues exhibited considerable inhibition of COX-II (44–60%) at 100 μM. Among the synthesized compounds, (2′-fluoro-[1,1′-biphenyl]-4-yl)(2-hydroxy-4,6-dimethoxy-3-(1-methyl-1*H*-pyrazol-5-yl)phenyl)methanone (**PYZ44**), 3′-bromo-[1,1′-biphenyl]-4-yl)(2-hydroxy-4,6-dimethoxy-3-(1-methyl-1*H*-pyrazol-5-yl)phenyl)methanone (**PYZ45**), and 2-hydroxy-4,6-dimethoxy-3-(1-methyl-1*H*-pyrazol-5-yl)phenyl)(4′-methoxy-[1,1′-biphenyl]-4-yl)methanone (**PYZ46**) were found to be active anti-inflammatory agents in addition to potent antioxidant activity. The compound **PYZ46** having methoxy substituent at the fourth position of phenyl ring appeared to be the most active compound in this series against COX-II enzyme (Figure 10).

Tonk et al.^70^ reported a series of pyrazolo[4,3-*c*]cinnoline derivatives and evaluated for anti-inflammatory and antibacterial activity as compared to naproxen. The compounds that exhibited favorable anti-inflammatory potency were also assessed for their ulcerogenic and lipid peroxidation activity. Among all, 7-chloro-8-fluoro-3-methyl-1*H*-pyrazolo[4,3-*c*]cinnolin-1-yl)(*p*-tolyl)methanone (**PYZ47**) and 7-chloro-8-fluoro-1*H*-pyrazolo[4,3-*c*]cinnolin-1-yl)(4-methoxyphenyl)methanone (**PYZ48**) were found to be optimal anti-inflammatory agents having percentage inhibition of 74 and 80%, respectively, after 4 h. Moreover, docking studies revealed that **PYZ48** was found to exhibit stronger binding interaction with COX-II active site than **PYZ47**. These compounds did not exhibit specific antibacterial activity against tested strains. Methyl or methoxy group at the *p*-location of phenyl group could be responsible for higher potency (Figure 10). Bhardwaj et al.^71^ had designed and synthesized a succession of 5-azidopyrazole derivatives and assessed their anti-inflammatory activity in cell-based assays. Among all, 4-(4-fluorophenyl)-1-(3-methyl-1-(4-(methylsulfonyl)phenyl)-1*H*-pyrazol-5-yl)-1*H*-1,2,3-triazole (**PYZ49**) and 4-(1-(3-methyl-1-(4-(methylsulfonyl)phenyl)-1*H*-pyrazol-5-yl)-1*H*-1,2,3-triazol-4-yl)aniline (**PYZ50**) were found to be highly potent anti-inflammatory agents. **PYZ49** and **PYZ50** were 25-times more potent than standard drug Celecoxib (*ED*_*50*_ = 10.8 mg kg^–1^ at 3 h). Molecular docking studies revealed that these two compounds optimally fit within the active site of COX-II (*E*_intermolecular_ = −15.9 and −16.8 kcal/mol) (Figure 10).

### 4.2 Isoxazole Derivatives as Anti-inflammatory Agents

**Figure 11:**
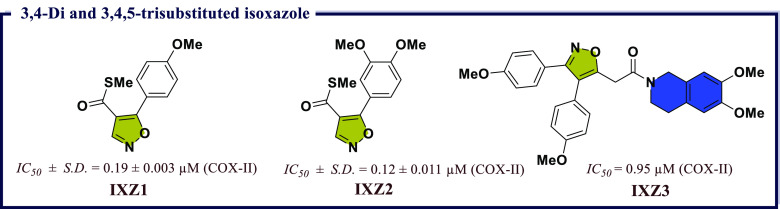
3,4-Di- and 3,4,5-trisubstituted isoxazole as potential COX-II inhibitors

Compounds comprising isoxazole moiety serve as a structural framework for drug used to treat different type of disease and infections. There are many recognized drugs like Parecoxib and Leflunomide that are constructed using isoxazole moiety as basic framework. Leflunomide serves as an immunosuppressive drug and exhibits therapeutic applications when used alone or in combination with other drugs in rheumatoid or transplantation. Parecoxib is used as an anti-inflammatory drug and acts on COX-II. These applications lead researchers to derive novel isoxazole derivatives associated with biological applications.^72^ Isoxazole having an azole with O atom next to N exhibits a diverse range of biological activity, and especially shows COX-II and HIV inhibitory activity. Isoxazole ring containing compounds are found to exhibit selective COX-II inhibitory activity. So, Joy et al.^73^ had reported two series of novel isoxazole derivatives comprising methoxy and dimethoxy functional groups with good COX-I/II inhibitory potential. Herein, *S*-methyl-5-(4-methoxyphenyl)isoxazole-4-carbothioate (**IXZ1**) and *S*-methyl 5-(3,4-dimethoxyphenyl)isoxazole-4-carbothioate (**IXZ2**) exhibited strong COX-II inhibitory activity, and none of the compounds showed activity against COX-I. In addition, a docking experiment was carried out on both of the compounds. Thus, these two compounds can be good candidates in the future for COX-II inhibition (Figure 11). Perrone et al.^74^ reported a series of isoxazole based scaffold inhibitors. The reported compounds were tested to determine COX-I/II inhibitory activity. The results determined the presence of both COX-I and COX-II inhibitory activity in different groups of compounds. Among them, 2-(3,4-*bis*(4-methoxyphenyl)isoxazol-5-yl)-1-(6,7-dimethoxy-3,4-dihydroisoquinolin-2(1*H*)-yl)ethan-1-one (**IXZ3**) exhibited most potent activity against COX-II with an *IC*_*50*_ value of 0.95 μM. It had showed interaction with P-glycoprotein. Docking studies revealed the molecular aspects of the observed COX selectivity (Figure 11).

### 4.3 Oxadiazole Derivatives as Anti-inflammatory Agents

Oxadiazole was found to be an important skeleton associated with various biological activities like antifungal, antimalarial, analgesic, anti-inflammatory, analgesic, and antituberculosis activities. Zibotentan drug in clinical trials was found to exhibit high anticancer activity.^75^ Also, 1,3,4-oxadiazoles revealed a diverse range of biological activities. 1,2,4-Oxadiazoles were also reported as anti-inflammatory agents with selective COX-II inhibition. These facts enthused scientists to develop more oxadiazole derivatives and evaluate their biological potential.^76^

**Figure 12:**
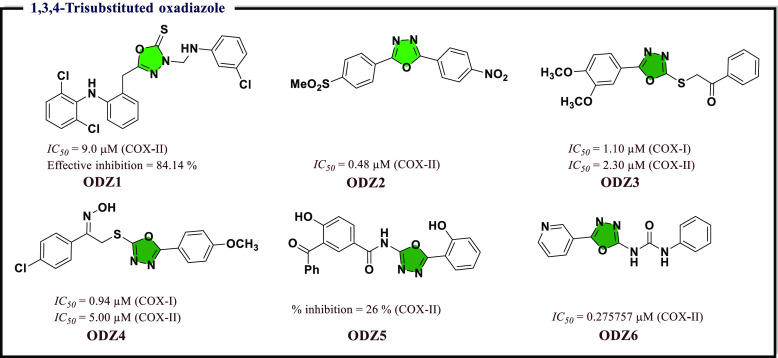
1,3,4-Trisubstituted oxadiazole as potential COX-II inhibitor.

Palkar et al.^77^ had reported a few new 5-[2-(2,6-dichlorophenylamino)benzyl]-3-(substituted)-1,3,4-oxadiazol-2(3*H*)-thione derivatives that were confirmed to possess acute anti-inflammatory activity against COX-I/II *in vivo*. Along with this, the tested compounds were screened for analgesic activity and antipyretic activity using an acetic acid induced writhing model and a yeast induced pyrexia model, respectively. Among the reported compounds, 3-(((3-chlorophenyl)amino)methyl)-5-(2-((2,6-dichlorophenyl)amino)benzyl)-1,3,4-oxadiazole-2(3*H*)-thione (**ODZ1** in Figure 12) exhibited the most promising and significant anti-inflammatory activity with an *IC*_*50*_ value 9.0 μM. This compound also displayed a lower degree of ulcerogenic potency (2.1–2.6) when compared to Diclofenac (5.5–6.2), Aspirin (4.7–5.2), and Flurbiprofen (5.9–6.6) tested in this work. Ulcerogenic potency is a well-established fact that the vast majority of anti-inflammatory (NSAIDs) medications available in the market cause ulcers and therefore are known to possess ulcerogenic potential.^78^ Ulcerogenic potential thus is defined as the tendency of NSAIDs majorly non selective COX-II or preferential COX-I inhibitors to induce the ulcer as a major side effect on their long-term use. Primarily the inhibition of cytoprotective prostaglandin that chiefly includes PGE_2_ and PGI_2_ is known to increase the gastric secretions that consequently damages the gastric mucosa, thereby eliciting the ulcer.^79^ As far as research on NSAIDs is concerned, the results also revealed that the extent of drug dissolved in gastric pH is also the major factor to cause ulceration apart from the inhibition of cytoprotective prostaglandin synthesis.^80^ Few reports suggest that steric, electronic, and quantum chemistry may significantly affect how ulcerogenic NSAIDs are affected.^81^

In another study, Grover et al.^82^ reported a series of 2,5-diaryl-1,3,4-oxadiazoles derivatives. The synthesized compounds were evaluated for *in vitro* COX-I/II inhibitory activity. All of the compounds displayed COX-II selective inhibition. The compounds having a methyl sulfonyl group exhibited selective COX-II inhibitory activity. Among them, 2-(4-(methylsulfonyl)phenyl)-5-(4-nitrophenyl)-1,3,4-oxadiazole (**ODZ2** in Figure 12) was found to be most potent and selective against COX-II having an *IC*_*50*_ value of 0.48 μM and *S.I.* value of 132.83. It has been concluded that EWG at the phenyl ring enhanced COX-II inhibition and EDG decreased it. The compounds that passed *in vitro* screening were further assessed *in vivo* and were confirmed to show anti-inflammatory activity comparable to or better than that of Celecoxib (the standard compound in this study). To check the cytotoxicity of the most potent compounds, cytotoxicity evaluation was carried out against RAW 264.7 and J774A.1 cells. The tested compounds did not show any cytotoxicity. *In silico* molecular docking studies revealed that the compounds having a methyl sulfonyl group showed a greater docking score in comparison to thiomethyl derivatives for COX-II. This can be due to a large volume of COX-II side pocket that promotes the insertion of a methyl sulfonyl group. Compound **ODZ2** showed hydrogen-bonding interaction with Tyr385 through the oxygen atom of SO_2_CH_3_ as Celecoxib.

COX inhibitors are often associated with numerous side effects. So, nitric oxide releasing NSAIDs have been found to be anti-inflammatory scaffold due to their gastric careful properties. Nitric oxide can control the discharge of inflammatory intermediaries from macrophages, leukocytes, and endothelial cells. In addition, oxadiazoles are associated with various biological activities. Thus, a series of novel 1,3,4-oxadiazole/oxime (NO donating group) hybrids were reported by Ellah et al.^83^ The synthesized compounds were screened for antioxidant, anti-inflammatory, and ulcerogenic activities. The results indicated that the compounds exhibited noteworthy anti-inflammatory activity with 69.60–109.60% of Indomethacin (INM) activity after 4 h. Some of the compounds had good COX-II inhibition (*IC*_*50*_ = 2.30–6.13 μM) under condition compared to INM (*IC*_*50*_ = 24.60 μM). Among all, 2-((5-(3,4-dimethoxyphenyl)-1,3,4-oxadiazol-2-yl)thio)-1-phenylethan-1-one (**ODZ3**) and (*Z*)-1-(4-chlorophenyl)-2-((5-(4-methoxyphenyl)-1,3,4-oxadiazol-2-yl)thio)ethan-1-one oxime (**ODZ4**) showed the most potent inhibitory activity against COX-I and COX-II. Compound **ODZ4** had the capability to inhibit both COXs noncompetitively (Figure 12).

Puttaswamy et al.^84^ reported novel 2-(4-hydroxy-3-benzoyl)benzamide-5-phenyl-1,3,4-oxadiazole derivatives and evaluated them for *in vitro* anti-inflammatory activity by screening against human red blood cells. From this evaluation, it was found that compound **ODZ5** with hydroxyl substituent at the *o*-position of the phenyl group attached to the fifth carbon of the oxadiazole ring and exhibited noteworthy membrane stabilizing activity. The inhibitory concentration (*IC*_*50*_) of **ODZ5** was found to be 153.43 μg/mL in comparison to the standard drug INM (*IC*_*50*_ = 121.68 μg/mL), which exhibited inflammatory angiogenesis activity. *In vivo*, 3-benzoyl-4-hydroxy-*N*-(5-(2-hydroxyphenyl)-1,3,4-oxadiazol-2-yl)benzamide (**ODZ5**) exhibited anti-inflammatory activity and inhibited 26% activity of COX-II. *In silico* molecular docking studies revealed the presence of H-bonding interaction of **ODZ5** with Asp125 and also displayed ionic interaction between the oxadiazole ring and carboxylic group of amino acid Ala86 (Figure 12).

Sayed et al.^85^ reported novel heterocyclic oxadiazoles, *viz*. 2-subsituted-5-(4-pyridyl)-1,3,4-oxadiazoles, 2-subsituted-5-(3-pyridyl)-1,3,4-oxadiazoles, and 2-subsituted-5-(phenyl or 4-chlorophenyl-1,3,4-oxadiazoles. All compounds were evaluated for their *in vitro* COX-I/II inhibitory activity; new therapeutic approaches assumed cytotoxic effect associated with selective COX-II inhibition comparable to the standard drugs Indomethacin, diclofenac sodium, and Celecoxib. Then nine selected compounds were subjected to cytotoxic screening against UO-31 renal cancer cell line using MTT assay. The tested compounds showed potent activity against EGFR with the highest activity being observed for compound **ODZ6**, showing nearly double the potency of the reference drug Erlotinib. Moreover, molecular docking and dynamics were performed against EGFR in order to understand the possible binding interactions underlying between these small molecules and kinase enzyme ATP binding pocket essential amino acids. Finally, it was observed that 1-phenyl-3-(5-(pyridin-3-yl)-1,3,4-oxadiazol-2-yl)urea (**ODZ6**) had a potential to serve as a lead compound for developing novel anticancer therapeutic agents (*IC*_*50*_ = 0.2757357 μM) (Figure 12).

### 4.4 Pyrrole, Pyrolidine, and Imide Derivatives as Anti-inflammatory Agents

**Figure 13:**
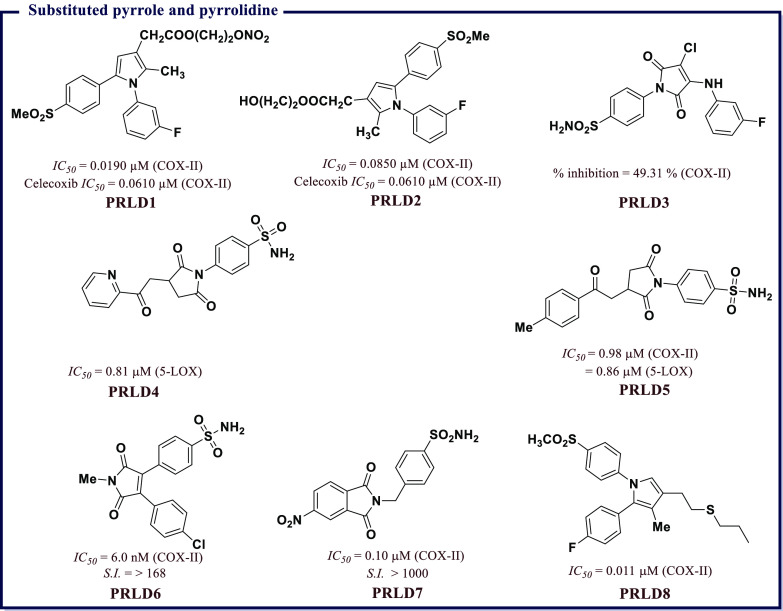
Substituted pyrrole and pyrrolidine as potential COX-II inhibitors.

Among well-known NSAIDs, pyrrole ring derivatives are of noteworthy interest. Some of the representative examples are benzo[*b*]pyrrole derivatives such as Indomethacin, Acemetacin, and Etodolac, and pyrrole derivatives like tolmetin and ketorolac. These compounds block prostaglandin synthesis by nonselective inhibition of COX-I and COX-II (Indomethacin, Acemetacin, Tolmetin, and Ketorolac) or by selective inhibition of COX-II (Etodolac).^86^ Biava et al.^87^ reported a series of pyrrole derived nitroxy esters and their corresponding alcohols. The synthetic molecules were assessed for biochemical inhibition of COX-II and their anti-inflammatory and antinociceptive potencies *in vivo*. Among the screening compounds, 2-(nitrooxy)ethyl-2-(1-(3-fluorophenyl)-2-methyl-5-(4-(methylsulfonyl)phenyl)-1*H*-pyrrol-3-yl)acetate (**PRLD1**) and 2-hydroxyethyl 2-(1-(3-fluorophenyl)-2-methyl-5-(4-(methylsulfonyl)phenyl)-1*H*-pyrrol-3-yl)acetate (**PRLD2**) were found to be the preferential inhibitors of COX-II (Figure 13). Reale et al.^88^ also screened a collection of synthetic 1,5-diarylpyrrol-3-sulfur derivatives for their COX-II inhibition using biochemical and cell-based assays. Few sulfoxides and sulfones showed appreciable COX-II inhibitory activity as their *IC*_*50*_ values ranged between 0.034 and 0.060 μM. For example, 1-(4-fluorophenyl)-2-methyl-5-(4-(methylsulfonyl)phenyl)-3-(2-(propylthio)ethyl)-1*H*-pyrrole (**PRLD8** in Figure 13) was identified as a potent inhibitor of COX-II (*IC*_*50*_ = 0.011 μM) *in vitro* with confirmed anti-inflammatory and antinociceptive activities *in vivo*.

Firke et al.^89^ reported a few maleimide analogs containing benzenesulfonamide scaffolds with confirmed COX-I/II inhibitory activity *in vitro* and their anti-inflammatory potency using the carrageenan induced rat paw edema method. 4-(3-Chloro-4-((3-fluorophenyl)amino)-2,5-dioxo-2,5-dihydro-1*H*-pyrrol-1-yl)benzenesulfonamide (**PRLD3**) was the most potent molecule as it was able to achieve 49.31% inhibition of COX-II at 1 μM concentration. Jan et al.^90^ reported a couple of *N*-substituted pyrrolidine-2,5-dione derivatives, 4-(2,5-dioxo-3-(2-oxo-2-(pyridin-2-yl)ethyl)pyrrolidin-1-yl)benzenesulfonamide (**PRLD4**) and 4-(2,5-dioxo-3-(2-oxo-2-(*p*-tolyl)ethyl)pyrrolidin-1-yl)benzenesulfonamide (**PRLD5**), that exhibited COX-II inhibition and anti-inflammatory potentials. Both compounds had an *IC*_*50*_ of ∼0.8 μM, which was on par with the standard compound (Zileuton with an *IC*_*50*_ of 0.63 μM). In another study, Kim et al.^91^ developed a series of 1*H*-pyrrole-2,5-diones as potent COX-II inhibitors. For example, 4-(4-(4-chlorophenyl)-1-methyl-2,5-dioxo-2,5-dihydro-1*H*-pyrrol-3-yl)benzenesulfonamide (**PRLD6** in Figure 13) inhibited COX-II (*IC*_*50*_ = 6.0 nM) with an *S.I.* ≥ 168, thereby confirming its specificity toward anti-COX-II activity. A group of cyclic imides was developed as selective COX-II inhibitors by Suwaidan et al.^92^ Some synthesized compounds proved to be potent COX-II inhibitors with *IC*_*50*_ ranging between 0.1 and 4.01 μM. Structure–activity relationship identified 4-((5-nitro-1,3-dioxoisoindolin-2-yl)methyl)benzenesulfonamide (**PRLD7**) as a potent (*IC*_*50*_ = 0.1 μM) and selective (*S.I.* > 1000) COX-II inhibitor. **PRLD7** was also able to reduce inflammation *in vivo* (*ED*_*50*_ = 72.4 mg/kg) relative to diclofenac (*ED*_*50*_ = 114 mg/kg). Molecular docking studies revealed that the homosulfonamide fragment of **PRLD7** was situated deep inside the 2°-pocket of the COX-II active site, where the SO_2_NH_2_ group participates in H-bonding interaction with Gln192 (2.95 Å), Phe518 (2.82 Å), and Arg513 (2.63 and 2.73 Å) (Figure 13).

### 4.5 Thiazole and Thiadiazole Derivatives as Anti-inflammatory Agents

**Figure 14:**
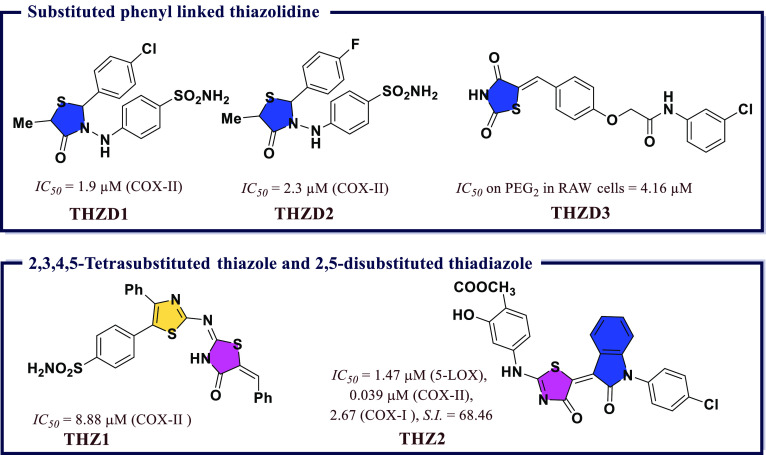
Substituted phenyl linked thiazolidine, 2,3,4,5-tetrasubstituted thiazole, and 2,5-disubstituted thiadiazole as potential COX-II inhibitors

NSAIDs are among commonly used analgesics and antipyretics medications. Continuous efforts have been devoted by medicinal chemists to reduce the inflammation in several cancers by developing novel NSAIDs. The exact mechanism of NSAIDs to induce apoptosis and inhibit angiogenesis in cancer cells was not explained. However, both COX-dependent and independent pathways were reported to have a role.^93^ Abdelazeem et al.^94^ synthesized a series of diphenylthiazole-substituted thiazolidinone and assessed their dual potency of COX-II inhibition and anticancer activity. Cytotoxicity assay revealed that the most effective compounds have *IC*_*50*_ values between 8.88 and 19.25 μM against five different human cancer cell lines. Interestingly, the most potent anticancer compound, i.e., 4-(2-((*E*)-((*Z*)-5-benzylidene-4-oxothiazolidin-2-ylidene)amino)-4-phenylthiazol-5-yl)benzenesulfonamide (**THZ1**), displayed good COX-II inhibition comparable to that of Celecoxib (*IC*_*50*_ = 8.88 μM). Strong binding affinity of **THZ1** for COX-II was revealed in molecular docking studies. These results collectively demonstrated the positive activity of new compounds as indicators of future growth in potential anticancer agents (Figure 14).

A series of 4-aminosalicylate based thiazolinones were reported by Allah et al.^95^ The synthetic compounds were screened for their cytotoxicity behavior and found to exhibit less cytotoxicity. Further, the compounds with good activities were investigated for their anti-inflammatory potential with dual balanced inhibition. Among all, two compounds (*IC*_*50*_ = 41 and 44 μM) were found equipotent to Celecoxib (*IC*_*50*_ = 49 μM) and Zileuton (*IC*_*50*_ = 15 μM), while methyl 4-[(5*Z*-(4-chlorobenzyl)-2-oxoindolin-3-ylidene)-4(*H*)-oxo-1,3-thiazol-2-yl)amino]-2-hydroxybenzoate (**THZ2**) had the most potent dual inhibitory activity for COX-II and 5-LOX. *In vivo* anti-inflammatory activity of **THZ2** (% inhibition = 63 ± 5) had an anti-inflammatory activity comparable to those of Indomethacin and Celecoxib (% inhibition of edema = 60 ± 9) and higher than that of diclofenac potassium (% inhibition = 52 ± 29) (Figure 14).

### 4.6 Thiazolidine Derivatives as Anti-inflammatory Agents

Abdellatif et al.^96^ synthesized two sequences of thiazolidin-4-one derivatives. The synthesized compounds were evaluated for their anti-COX-I/II activity (*in vitro*) and anti-inflammatory potency (*in vivo*). Two compounds, 4-((2-(4-chlorophenyl)-5-methyl-4-oxothiazolidin-3-yl)amino)benzenesulfonamide (**THZD1**) and 4-((2-(4-fluorophenyl)-5-methyl-4-oxothiazolidin-3-yl)amino)benzenesulfonamide (**THZD2**), inhibited COX-II with *IC*_*50*_ values of 1.9 μM and 2.3 μM, respectively, in comparison to reference drug Celecoxib (*IC*_*50*_ = 1.33 μM). It is worth mentioning here that both **THZD1** and **THZD2** were selective toward COX-II over COX-I. After successful prescreening of the compounds for *in vitro* COX inhibition, the anti-inflammatory potential of all the molecules was also evaluated *in vivo* and found to be greater than that of Celecoxib. Both compounds also displayed maximum % of edema inhibition, i.e., 61.8% and 67%, respectively, after 3 h. The inhibitory potential of **THZD1** was equipotent to Celecoxib; however, **THZD2** was found to be more potent than Celecoxib. In addition to this, reduction in ulcerogenic potential (Ulcerogenic potential thus is defined as the tendency of NSAIDs majorly non selective COX-II or preferential COX-I inhibitors to induce the ulcer as a major side effect on their long-term use) was also observed for these compounds in comparison to Celecoxib (85% and 92%). A similar type of molecular interaction as that present in Celecoxib was observed for **THZD1** and **THZD2**. The *p*-sulfamoylphenylamino moiety of both compounds fits strongly in the secondary pocket of COX-II surrounded by His75, Ser339, Arg499, and Gln178. **THZD2** showed three H-bonding interactions with the amino acids His75 (distance = 3.16 Å), Ser339 (distance = 2.02 Å), and Arg499 (distance = 2.69 Å) with a docking score of −16.40 kcal/mol. However, **THZD1** showed two H-bonding interactions with the amino acids Ser339 (distance = 2.14 Å) and Arg499 (distance = 2.78 Å) with a docking score of −17.15 kcal/mol. These two compounds can be good candidates for the future development of novel drugs (Figure 14). A series of 20 eight compounds based on thiazolidine-2,4-dione moiety was developed by Ma et al.^97^ The synthesized compounds evaluated for inhibitory potency for the production of nitric oxide, iNOS and PGE_2_. The oral administration of **THZD3** possessed protective properties in different *in vivo* models including adjuvant-induced arthritis rat model and carrageenan-induced paw edema model at the dose of 50 mg/kg. Among all, (*Z*)-*N*-(3-chlorophenyl)-2-(4-((2,4-dioxothiazolidin-5-ylidene)methyl)phenoxy)acetamide (**THZD3**) exhibited potent inhibitory activity against iNOS (*IC*_*50*_ = 8.66 μM), iNOS-mediated NO, and COX-II derived PGE_2_ production (*IC*_*50*_ = 23.55 and 4.16 μM) on LPS induced Raw 264.7 cells. Docking study displayed that **THZD3** perfectly fit into the active site of murine iNOS and suppressed the expression of iNOS protein as determined by Western blot analysis (Figure 14).

### 4.7 Non-heterocyclic Compounds as Anti-inflammatory Agents

**Figure 15:**
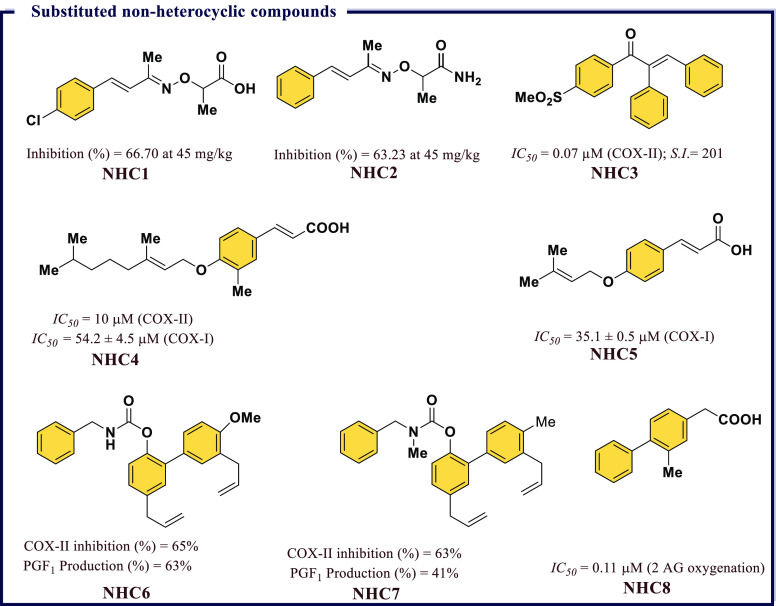
Substituted nonheterocyclic compounds as potential COX-II inhibitors.

Organic compounds possessing side chain heteroatoms in the form of imines, esters, acids, amides, thioamides, sulfones, ethers, and α,β-unsaturated carbonyl compounds possess a wide range of biological activities especially COX inhibitory properties. For example, Gamal et al.^98^ reported a few novel substituted benzylidene acetone oxime ethers that were able to reduce inflammation in the carrageenan induced rat paw edema model. The highest anti-inflammatory response was particularly observed for 2-((((2*E*,3*E*)-4-(4-chlorophenyl)but-3-en-2-ylidene)amino)oxy)propanoic acid (**NHC1**) and 2-((((2*E*,3*E*)-4-phenylbut-3-en-2-ylidene)amino)oxy)propanamide (**NHC2**) that achieved >60% of edema reduction, which was equivalent to the standard drug diclofenac sodium. Among the series, the acetic acid derivatives were found to be more active than propanoic acid derivatives. The anti-inflammatory responses of both **NHC1** and **NHC2** were explored in detail by comparing their *ED*_*50*_ values with that of standard drug, i.e., diclofenac sodium, using three graded doses. Both **NHC1** and **NHC2** showed *ED*_*50*_ values of 29.92 mg/kg and 34.30 mg/kg, respectively, in comparison with diclofenac sodium with 30.65 mg/kg. The analgesic response observed for both these compounds had no better response comparaed with the standard drug. The ulcerogenic effect observed in **NHC1** was comparable with that of diclofenac sodium, which might be attributed to the presence of a free carboxylic group. However, negligible ulcerogenic effect was observed in **NHC2** due to the presence of an amide group. Both compounds possess higher therapeutic effect and less toxicity than diclofenac sodium. Molecular docking studies revealed the presence of extra interactions in the hydrophobic pocket of the enzyme in **NHC2** as compared with **NHC1**, which describes the selective inhibitory response of **NHC2** for COX-II (Figure 15).

Arfaie et al.^99^ studied the effect of geometrical parameters on the selectivity and COX-II inhibitory potential in a series of acyclic (*E*)- and (*Z*)-1,2,3-triaryl-2-propen-1-ones. All the compounds possess a methylsulfonyl pharmacophore at the C_1_ phenyl ring, and substituents were varied at the C_3_ position to explore the *in vitro* COX-I/COX-II structure–activity relationship. (*Z*)-1-(4-(Methylsulfonyl)phenyl)-2,3-diphenylprop-2-en-1-one (**NHC3**) was able to inhibit COX-II with an *IC*_*50*_ of 0.07 μM, which was comparable to the activity of Celecoxib (*IC*_*50*_ = 0.06 μM). A detailed geometrical and substituent effect showed higher selectivity and COX-II potency of *Z*-propenones over *E*-isomers (Figure 15).

Over the last 15 years, oxyprenylated secondary metabolites have attracted significant interest as potential phytochemicals due to valuable biological activities associated with them. 4′-Geranyloxyferulic acid (prenyloxycinnamic acid) is one of the oxyprenylated secondary metabolites having a geranyl chain attached to the phenolic group related biosynthetically to ferulic acid. A detailed study was carried by Epifano and co-workers and displayed a 41% reduction in edema formation by 4′-geranyloxyferulic acid compared with Indomethacin (62%). These studies inspired Genovese et al.^100^ to explore the anti-inflammatory response of novel natural and semisynthetic derivatives of prenyloxycinnamic acid. The results identified that 3-(4′-gernyl-3′-methoxy)phenyltrans propenoic acid (**NHC4**) and 3-(4′-isopentenyloxy)phenyl-2-trans propanoic acid (**NHC5**) displayed significant *IC*_*50*_ values (54.2 ± 4.5 μM and 35.1 ± 0.5 μM, respectively) against COX-I. The anti-inflammatory response in terms of COX-II inhibition was recorded for **NHC4** on isolated monocytes stimulated with LPS (10 μg/mL). A 41% reduction in edema formation at a dose of 0.3 μmol/cm^2^ was observed for **NHC4** in comparison with 62% reduction observed with Indomethacin. A dose dependent inhibition of LPS-induced COX-II expression was also observed for both compounds, i.e., **NHC4** and **NHC5** (Figure 15).

Over a period, traditional medicines have been derived from the bark of the root and stem of the *Magnolia* family. 4-*O*-Methylhonokiol is one of the bioactive compounds with promising anti-inflammatory response isolated from plants of *Magnolia* family. Lee et al.^101^ synthesized a sequence of COX-II selective 4-*O*-methylhonokiol analogs using a key strategy of modifying potential soft spots (e.g., phenol and olefin) or by altering the polar surface area via incorporating heterocycles such as isoxazole and triazole. All the tested compounds were explored for their inhibitory potential against COX-II. Moreover, the PGF_1_ production was compared with that of Celecoxib and 4-*O*-methylhonokiol. Direct inhibition of COX-II enzyme was observed for all the synthesized compounds at 100 nM with an inhibition range of 22–65%. Moreover, most of them exhibited inhibitory effects and PGF_1_ production without macrophage NO production. Especially, 3′-5-diallyl-4′methoxy-[1,10-biphenyl]-2-yl-benzylcarbamate (**NHC6**) and 3′-5-diallyl-4′methoxy-[1,10-biphenyl]-2-yl-benzyl(methyl)carbamate (**NHC7**) exhibited more potent inhibitory activity against COX-II and PGF_1_ production. Higher % inhibition was shown by these compounds (65 and 63%) as compared to Celecoxib (60%) (Figure 15).

The COX-II catalyzed oxygenation of arachidonic acid (AA), endocannabinoids 2-arachidonoylglycerol (2-AG), and arachidonoylethanolamide (AEA) resulted in prostaglandin-H_2_ (PGH_2_) along with its glycerylesters (PGH_2_-G) and ethanolamides (PGH_2_-EA). These oxygenated products metabolized into PGs, which had unique role in macrophages and tumor. Marnett et al.^102^ observed that ibuprofen and mefenamic acid exhibited more rapid and selective inhibitiors of COX-II as compared to potent inhibitors of 2-AG and AEA than AA oxygenation. Moreover, they explained the substrate selectivity in terms of binding site alteration. This alteration inhibits 2-AG and AEA oxygenation only, and AA oxygenation requires rapid and reversible binding of inhibitors to both the sites. A substrate-selective (*R*)-enantiomer of the arylpropionic acid (Profen) was developed, which was found to be inactive toward COX-II, which might be due to rapid unidirectional inversion to (*S*)-enantiomer *in vivo*. In an attempt to overcome the disadvantage associated with these compounds, the synthesis of achiral derivatives of five Profen scaffolds was achieved. The synthesized compounds were then evaluated for substrate-selective inhibition using *in vitro* and cellular assays. The size of the substituents had a significant effect on the inhibitory strength, as smaller substituents enable greater potency but lesser selectivity. Inhibitors based on the flurbiprofen scaffold possessed greatest potency and selectivity, as desmethylflurbiprofen (**NHC8**) exhibited an *IC*_*50*_ of 0.11 μM against 2-AG oxygenation. Each flurbiprofen derivative had a lower *IC*_*50*_ for 2-AG inhibition and lower % inhibition of AA oxygenation compared with the other derivatives of the each class. The crystal structure of **NHC8** complexed with COX-II demonstrated a similar binding mode as observed with other profens. Desmethylflurbiprofen exhibited a half-life in mice comparable to that of ibuprofen. The data presented suggest that achiral profens can act as lead molecules toward for substrate-selective *in vivo* COX-II inhibition (Figure 15).

**Figure 16:**
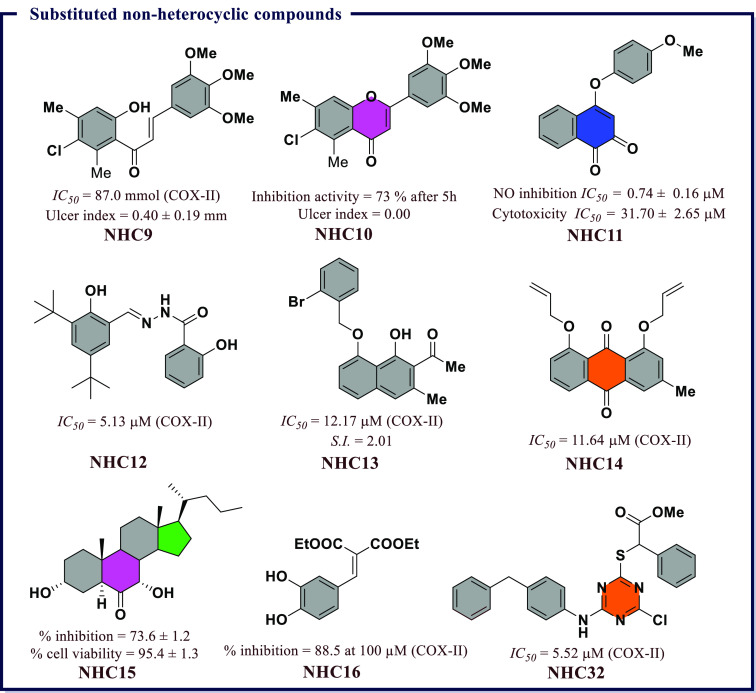
Substituted nonheterocyclic compounds as potential COX-II inhibitors.

In order to study the effect of ring architecture and different substituents, Bano et al.^103^ evaluated the anti-inflammatory activity of a novel series of 2′-hydroxychalcones, 2′-methoxychalcones, and their corresponding cyclic counterparts, i.e., flavanones and flavones. The synthetic molecules exhibited mild to strong inhibition (26% to 91%) in the *in vivo* carrageenan induced rat paw edema model. A strong substituents effect was observed, where incorporation of oxy group at *meta* position led to a significant increase in activity. Moreover, cyclization of 5′-chloro-2′-hydroxy-4’6′-dimethyl-3,4,5-trimethoxychalcone (**NHC9**) (% inhibition = 90.57) with its corresponding 6-chloro-5,7-dimethyl-3′,4′,5′-trimethoxyflavone (**NHC10**) (% inhibition = 73.33%) resulted in a decrease in % inhibition. **NHC9** showed inhibitory activity in LPS induced TNF-α production with COX-I (*IC*_*50*_ = 87.5 μmol) and COX-II (*IC*_*50*_ = 87.0 μmol) as compared to Indomethacin (*IC*_*50*_ = 0.063 μmol against COX-I and *IC*_*50*_ (0.48 μmol) against COX-II, respectively. Among the flavone derivatives, **NHC10** exhibited highest potency with anti-inflammatory activity of 68% at 3 h and 73% at 5 h (Figure 16). β-Lapachone is a naturally derived chemotherapeutic agent with potential anti-inflammatory effects and is found in the lapacho tree in South America. To develop new anti-inflammatory agents, Tseng et al. reported a new series of β-LAPA derivatives and explored their anti-inflammatory properties. It has been revealed that 4-(4-methoxyphenoxy)naphthalene-1,2-dione (**NHC11**) inhibited cytokines released in LPS-induced raw 264.7 cells. The study suggested that the anti-inflammatory activity of **NHC11** was linked with the suppression of NF-jB and MAPK signaling pathways. A low cytotoxicity (*IC*_*50*_ = 31.70 μM) and the potent anti-inflammatory activity exhibited by **NHC11** establish this as a potential lead for developing new anti-inflammatory agents (Figure 16).

There was a strong association between colorectal cancer (CRC) and chronic inflammation. It had been well established that the dual inhibitors of COX-II and 5-LOX are more potent than targeting COX or LOX alone. Ghatak et al.^104^ was inspired by the few reports on dual inhibitors in case of CRC and developed a novel series of *di*-*tert*-butyl-phenylhydrazone ligands as dual inhibitors. Among all, (*E*)-*N*′-(3,5-di-*tert*-butyl-2-hydroxybenzylidene)-2-hydroxybenzohydrazide (**NHC12**) was found to be the most potent in reversing the drug resistance with a significant *IC*_*50*_ (5.13 μM) in comparison to Celecoxib (*IC*_*50*_ = 6.49 μM). However, higher values for COX-I inhibition exhibited by all compounds indicated their preferential affinity for COX-II enzyme. In addition to this, **NHC12** also exhibited an *IC*_*50*_ value of 8.0 μM against LOX-5. *In silico* molecular docking studies revealed a good fit of these compounds in the COX-II and 5-LOX protein cavities and exhibited enhanced antiproliferative potency compared to standard dual COX/LOX inhibitor, *viz*. Licofelone (Figure 16).

Nepodin and Chrysophanol had two naturally derived chemotherapeutic agents with significant COX inhibitory potential. In order to further optimize these lead molecules, Grover et al.^105^ had synthesized Nepodin and Chrysophanol derivatives by chemical modifications of the OH groups. All the synthesized derivatives were evaluated for COX-I and COX-II inhibitory potential to develop structure–activity relationships. The COX-II inhibitory potency and selectivity were determined by aromatic substitution. Within aromatic substitution, 1-(8-(2-bromobenzyloxy)-1-hydroxy-3-methylnaphthalen2-yl)ethanone (**NHC13**) displayed the highest COX-II selectivity (*S.I.* = 2.01), which might be due to the larger size of bromine. Among Chrysophanol derivatives, 1,8-bis(allyloxy)-3-methylanthracene-9,10-dione (**NHC14**) showed the highest COX-II inhibition (*IC*_*50*_ = 11.64 μM). ADMET properties of most active compounds were found to be similar to naproxen and displayed good oral absorption, solubility, and lipophilicity. Further, the activity profile of the molecule is supported by *in silico* molecular docking studies, which render **NHC13** as a lead molecule for future drug development (Figure 16).

Pirinixic acids analogs were reported as dual mPGES-1/5-LOX (5-lipoxygenase) inhibitors; based on this, Kang et al.^106^ developed novel *S*-triazine derivatives as isosteric analogs of pirinixic acid and screened their effects on prostaglandin (PGE_2_) generation in lipopolysaccharide (LPS)-induced RAW 264.7 cells. Among them, methyl 2-((4-((4-benzylphenyl)amino)-6-chloro-1,3,5-triazin-2-yl)thio)-2-phenylacetate (**NHC32** in Figure 16) was noncytotoxic with 90% inhibition of PGE_2_ production with an *IC*_*50*_ value of 5.52 μM. A variety of pro-inflammatory factors such as NO and TNF-α were released by activated microglia upon simulation of neuronal inflammation. The control of NO production in microglia was a potential target as it is excessive accumulation known to be toxic and thought to contribute to neuronal injury. Keeping in view the high penetration ability of steroidal compounds, Yang et al.^107^ synthesized derivatives of 5*a*-cholestan-6-one and tested their anti-inflammatory potencies in LPS-stimulated BV-2 microglia cells. Several analogs exhibited weak cell toxicity with potent NO production inhibitory activities. Among all, (3*R*,5*S*,7*S*,10*R*,13*R*,17*R*)-3,7-dihydroxy-10,13-dimethyl-17-((*R*)-pentan-2-yl)hexadecahydro-6*H* cyclopenta[*a*]phenanthren-6-one (**NHC15**) also expressively suppressed the expression of TNF-α and COX-II as well as inducible nitric oxide synthase (iNOS) in the cell-based assay. In addition to this, **NHC15** markedly reduced infarction volume in a focal ischemic mice model (Figure 16).

Hydroxycinnamic acids (HCAs) are naturally occurring phenolic compounds with good anti-inflammatory response. Modulation of carboxylic acid while considering the structural changes in commercially available NSAIDs leads to esters, which have the ability to suppress NF-κB and COX. In this context, the phenylpropanoid based frameworks were further explored by Silva et al.^108^ They had developed novel derivatives of hydroxycinnamic acids, including ethyl and diethyl esters, and explored them as COX inhibitors. The structural modifications in terms of esterification improved COX-I and COX-II inhibitory activities of these derivatives, where ethyl esters had good activity against COX-I. The most potent caffeic acid ethyl ester (**NHC16**) had 88.5 and 30.5% inhibitions against COX-II at 100 and 20 μM, respectively. However, the compound was found almost inactive against COX-I. Interestingly, diethyl esters showed selectivity toward COX-II. Docking studies revealed the presence of three H-bonds between **NHC16** and the active site of COX-II (4-OH···OH-Tyr355, 4-OH···NH-Arg120, and C=O···OH-Tyr385). However, only two H-bonds were observed with COX-I. Furthermore, Val523 residue in COX-II provides a wide hydrophobic pocket, which would accommodate diethyl esters (Figure 16).

**Figure 17:**
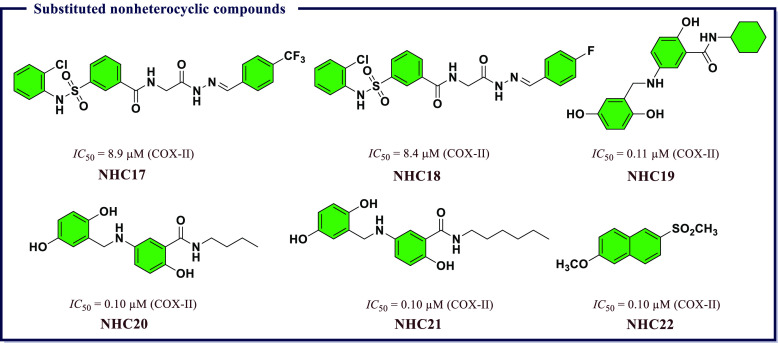
Substituted nonheterocyclic compounds as potential COX-II inhibitors.

*N*-Acylhydrazones (NAHs) represents a novel scaffold of selective COX-II inhibitors in the recently published independent pursuits of selective COX-II inhibitors. These findings on NAHs inspired Gorantla et al.^109^ to develop *N*-phenyl sulfonamide linked *N*-acyl hydrazones (NPS-NAH) by amalgamating acyl hydrazones with sulfonamide moiety using molecular hybridization approach and evaluating their anti-inflammatory potency. Among the screened compounds, 3-(*N*-(2-chlorophenyl)sulfamoyl)-*N*-(2-oxo-2-(2-(4-(trifluoromethyl)benzylidene)hydrazinyl)ethyl)benzamide (**NHC17**) and 3-(*N*-(2-chlorophenyl)sulfamoyl)-*N*-(2-(2-(1-(4-florophenyl)ethylidene)hydrazinyl)-2-oxoethyl)benzamide (**NHC18**) exhibited strong selective COX-II enzyme inhibition at *IC*_*50*_ values of 8.9 and 8.4 μM, respectively. These results validated the idea of exploiting the hybridization strategy for the identification of new *N*-phenyl sulfonamide-NAH derivatives with good anti-inflammatory response (Figure 17). Sulfasalazine, a derivative of 5-aminosalicylic acid (5-ASA), is a known as a potential agent for the treatment of inflammatory bowel disease. With the development of sulfasalazine, various attempts have been made to improve the pharmacokinetic and pharmacodynamics properties of 5-ASA. In a similar attempt, Mohamed et al.^110^ synthesized novel 5-ASA derivatives by incorporating them with Schiff’s bases and secondary amines using a molecular hybridization approach. The authors tested the synthetic compounds for anti-inflammatory activity using the *in vivo* carrageenan induced paw edema bioassay in male rats and compared them using Indomethacin (INM) as reference drug. All the compounds showed superior anti-inflammatory activity with edema inhibition in the range 40.5–114.1% compared to INM. Out of them, 5-[(2,5-dihydroxyphenyl)-methyl]amino-*N*-cyclohexylsalicylamides (**NHC19**) exhibited maximum anti-inflammatory activity with 114.12% edema inhibition and superior *GI* safety profile as compared to INM. Furthermore, **NHC19**, 5-[(2,5-dihydroxyphenyl)-methyl]amino-*N*-butylsalicylamides (**NHC20**), and 5-[(2,5-dihydroxyphenyl)-methyl]amino-*N*-hexylsalicylamides (**NHC21**) inhibit both COX-II and 5-LOX and are found to be most active against COX-II with significant *IC*_*50*_ values (0.11, 0.10, and 0.10, μM respectively). The inhibitory activity shown by all the compounds against 5-LOX was found to be 2–5-times greater than that of zileuton. *In silico* molecular docking studies revealed greater binding interactions of **NHC19**, **NHC20**, and **NHC21** with COX-II/5-LOX enzymes (Figure 17).

**Figure 18:**
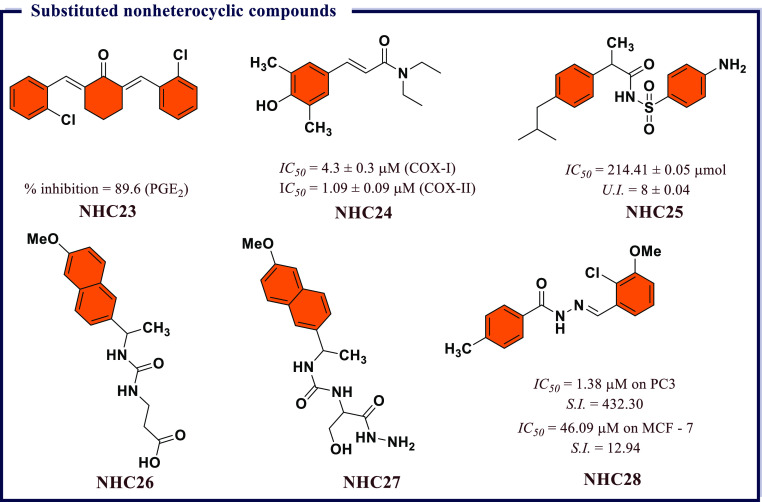
Substituted nonheterocyclic compounds as potential COX-II inhibitors.

In order to derive novel therapeutic agents from classic and commercial NSAIDs, Navarro et al.^111^ developed a series of compounds by replacing acetic or propionic acid with a methyl sulfone group and evaluated them for COX inhibitory activity using *in vitro* assay. The compound 2-methoxy-6-(methylsulfonyl)naphthalene (**NHC22** in Figure 17) screened was confirmed to exhibit anti-inflammatory activity in the carrageenan-induced paw edema method. **NHC22** exhibited superior inhibition of both COX-I and COX-II with *IC*_*50*_ values of 0.04 and 0.10 μM, respectively, as compared with standard drug naproxen (COX-I *IC*_*50*_ = 11.30 μM, COX-II *IC*_*50*_ = 3.36 μM). Kar et al.^112^ demonstrated the potential of diarylidene cyclohexanones as novel anti-inflammatory agents with significant inhibition against PGE_2_, which led to rationale of a structure–activity relationship. It was observed that substituents played a significant role in reducing PGE_2_ and were found to be an end product of the cyclooxygenase pathway. Most of the compounds inhibited the production of TNF-α induced PGE_2_ production by Hela cells and 5-LOX inhibition. Among them, 2,6-*bis*((*E*)-2-chlorobenzylidene)cyclohexan-1-one (**NHC23**) containing *o*-chloro had the best potency against PGE_2_ with 89.6% inhibition at 10 μM with an *IC*_*50*_ value of 6.7 μM (Figure 18).

Recently, a series of hydroxycinnamic acid derivatives were identified as modulators of human neutrophils with significant anti-inflammatory properties. In order to explore the ability of new phenolic cinnamic acid derivatives to selectively inhibit PGs production via COX-II, Ribeiro et al.^113^ synthesized a sequence of cinnamic acid derivatives, namely hexylamides, and evaluated them for COX-I and COX-II inhibitory activity in human blood. Structure–activity relationships revealed the essentiality of phenolic hydroxyl groups for both COX-I and COX-II inhibition. Furthermore, the presence of bulky hydrophobic *di*-*tert*-butyl groups in the phenyl ring strongly contributes to selective COX-II inhibition. It was discovered that (*E*)-*N*,*N*-diethyl-3-(4-hydroxy-3,5-dimethylphenyl)acrylamide (**NHC24**), possessing amide functionality, showed significant COX-I inhibition with good *IC*_*50*_ (4.3 ± 0.3 μM) to be higher than COX-II (*IC*_*50*_ = 1.09 ± 0.09 μM) (Figure 18).

A novel prodrug was synthesized by Asghar et al.^114^ by covalently coupling NSAIDs containing carboxylic groups with amino group functionalized anti-infectives. These derivatives have dual activity and better toxicity profiles. Along with this, the synthesized compounds have antiulcer, anti-inflammatory, and free-radical scavenging activities as compared to ibuprofen and sulphanilamide. Initially, the geometries of the tested compounds were optimized using the density functional theory (DFT) in the ground state. Then the geometrical parameters like bond lengths, torsion angle, bond angles, vibrational assignments, thermodynamics, and chemical shift were theoretically calculated and have good agreement with the experimentally determined data. Furthermore, *in silico* molecular docking was performed between *N*-(4-aminophenylsulfonyl)-2-(4-isobutylphenyl)propanamide (**NHC25**) and cyclooxygenase enzymes (COX-I/II). The result concluded that **NHC25** showed the highest binding affinities of −8.7 and −8.1 kcal/mol for COX-I and COX-II, respectively. *In vitro* and *in vivo* results revealed excellent activity for **NHC25** as compared to standard drug. The *in vitro* results of **NHC25** revealed higher inhibition of 48.4% as compared to ibuprofen (42.5%) (Figure 18).

Naproxen is a propionic acid derivative widely established as an anti-inflammatory drug to reduce the concentrations of PGE_2_ in different tissues. However, due to the presence of free-carboxylic acid in naproxen and its derivatives, they have associated gastrointestinal complications. Elhenawy et al.^115^ developed naproxenylamino acid derivatives and evaluated them for anti-inflammatory activity. Most of the synthesized compounds have excellent analgesic potency. Among all, 3-(3-(1-(6-methoxynaphthalen-2-yl)ethyl)ureido)propanoic acid (**NHC26**) and 1-(1-hydrazinyl-3-hydroxy-1-oxopropan-2-yl)-3-(1-(6-methoxynaphthalen-2-yl)ethyl)urea (**NHC27**) exhibited higher anti-inflammatory potency than naproxen with negligible ulcerogenic effects. These compounds may be considered as safer drugs than naproxen for treating inflammatory conditions. Further, the experimentally observed data supported by *in silico* molecular docking studies against COX-II indicated that both **NHC26** and **NHC27** showed stronger interactions with COX-II with a good oral bioavailability with no carcinogenesis affect (Figure 18).

Over the years, both selective and nonselective COX inhibitors have been widely studied for their effects in cancer treatment. In an attempt to explore this field, Senkardes et al.^116^ developed a series of sulfonylhydrazones and screened them for their cytotoxic activity contrary to prostate cancer (PC3), breast cancer (MCF-7), and L929 mouse fibroblast cell lines. It was observed that *N*′-[(2-chloro-3-methoxyphenyl)methylidene]-4-methylbenzenesulfonohydrazide (**NHC28**) was the most potent anticancer compound against both cancer cells lines with good selectivity (*IC*_*50*_ = 1.38 μM on PC3 with *S.I.* = 432.30 and *IC*_*50*_ = 46.09 μM on MCF-7 with *S.I.* = 12.94). Surprisingly, the most potent anticancer compounds showed 91% COX-II inhibition. Further investigation confirmed that the same compound displayed morphological alterations in PC3 and MCF-7 cells and promoted apoptosis through down-regulation of the Bcl-2 and upregulation of Bax expression. Molecular docking study of the tested compounds represented important binding modes that may be responsible for anticancer activity via inhibition of the COX-II enzyme. These studies open new horizons in the development of these compounds as potential anticancer agents (Figure 18).

### 4.8 Benzoxazole Derivatives as Anti-inflammatory Agents

Heterocycles, mainly benzoxazole, possess various biological activities such as anti-inflammatory, antibacterial, antifungal, analgesic, antihisatamine, antiparasitics, and antihelmintic effects. Literature revealed that benzoxazole moiety can be good template for COX-II inhibitory activity.^117^ Due to the broad spectrum of activities reported in literature, researchers have been worked on benzoxazole moiety, and some pioneering work on this has been discussed as follows.

**Figure 19:**
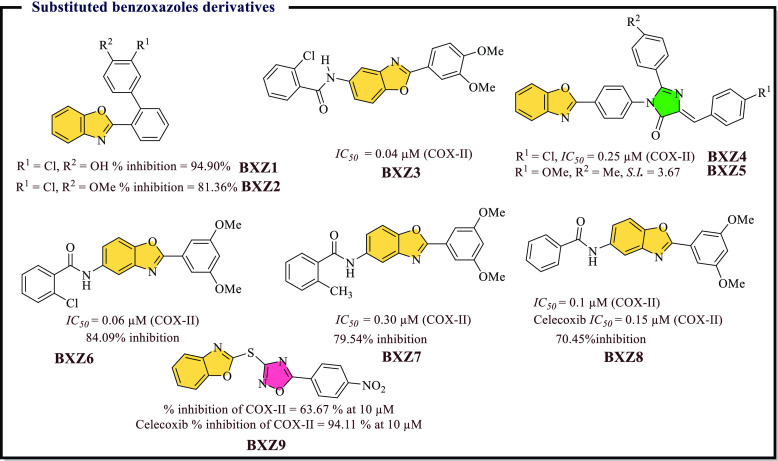
Substituted benzoxazoles derivatives as potential COX-II inhibitors.

Seth et al.^118^ synthesized a series of 2-(2-arylphenyl)benzoxazole derivatives using a scaffold hopping approach to develop novel COX-I/II inhibitors and evaluated the inhibitory activities of these molecules. Among them, 2-(3′-chloro-4′-methoxy-[1,1′-biphenyl]-2-yl)benzo[*d*]oxazole (**BXZ2**) showed the most effective and selective inhibitory activity against COX-II comparable to Celecoxib. One of the new synthesized compounds, 2′-(benzo[*d*]oxazol-2-yl)-3-chloro-[1,1′-biphenyl]-4-ol (**BXZ1**), was found to more potent than Celecoxib and diclofenac with 94% inhibition of COX-II. Computational studies revealed good binding affinities for both of these ligands with comparable docking scores as observed for Celecoxib. The presence of a V-shaped docking pose was similar to that of Celecoxib in the active site of COX-II, which proved COX-II selectivity. Compound **BXZ2** showed weak interaction of the methoxy group with the carbonyl group of Gln192; however, hydrogen-bonding interaction between hydroxyl group and carbonyl group of Gln192 was observed in case of **BXZ1**. These interactions were weak in the remaining synthesized compounds (Figure 19).

Mayank et al.^119^ synthesized a series of substituted *N*-(3,4-dimethoxyphenyl)-benzoxazole derivatives and screened their biological potential *via in vitro* COX-I and COX-II inhibitory activity. The most active COX inhibitors were tested for their ulcerogenic and anti-inflammatory activities. Among all, 2-chloro-*N*-(2-(3,4-dimethoxyphenyl)benzo[*d*]oxazol-5-yl)benzamide (**BXZ3**) showed 3.75-fold potency with % inhibition of 84.09 and *IC*_*50*_ value of 0.04 μM against COX-II as compared to reference drug Celecoxib and 1.02 μM against COX-I. Despite this, **BXZ3** also exhibited an improved gastric safety profile. SAR studies revealed that EWG at the *o*-position of phenyl ring and presence of nitro group at the *p*-position resulted in increased activity. In addition, benzoxazole moiety played a significant role, as nitrogen atom interacts with Arg120 via H-bond. Molecular docking studies revealed a noteworthy docking score for **BXZ3** (Figure 19).

Lamie et al.^120^ synthesized a novel series of 1,2-diaryl-4-substituted-benzylidene-5(4*H*)-imidazolone derivatives. The synthesized compounds were screened for COX-I/II and LOX inhibitory activity. Most of the compounds exhibited selectivity against COX-II with *IC*_*50*_ values in the range of 0.25 to 1.7 compared with reference drugs Indomethacin (*IC*_*50*_ = 9.47 μM) and Celecoxib (*IC*_*50*_ = 0.071 μM). Among all, (*Z*)-3-(4-(benzo[*d*]oxazol-2-yl)phenyl)-5-(4-chlorobenzylidene)-2-phenyl-3,5-dihydro-4*H*-imidazol-4-one (**BXZ4**) exhibited most potent activity against COX-II with *IC*_*50*_ value of 0.25 μM and (*Z*)-3-(4-(benzo[*d*]oxazol-2-yl)phenyl)-5-(4-methoxybenzylidene)-2-(*p*-tolyl)-3,5-dihydro-4*H*-imidazol-4-one (**BXZ5**) having *S.I.* = 3.67, which is nearly equal to that of Celecoxib (*S.I.* = 3.66). In addition to this, dual COX-II/LOX inhibitory activity was observed in the case of **BXZ4**. Furthermore, *in silico* molecular docking studies of **BXZ4** revealed that the nitrogen atom of benzoxazole showed one H-bond with His90 within the active site of COX-II. However, H-bond between carbonyl of imidazolone moiety and Asn180 was observed within the active site of LOX-5 (Figure 19).

Kaur et al.^121^ synthesized a series of *N*-(2-(3,5-dimethoxyphenyl)benzoxazole-5-yl)benzamide derivatives and evaluated them for *in vitro* COX-I/II inhibitory activity. Most of the compounds exhibited activity against COX-II with *IC*_*50*_ values less than 1 μM. The molecules showing significant inhibitory activity were further tested *in vivo* to evaluate their anti-inflammatory effects using the carrageenan-induced rat paw edema method. Among all, 2-chloro-*N*-(2-(3,5-dimethoxyphenyl)benzo[*d*]oxazol-5-yl)benzamide (**BXZ6**), *N*-(2-(3,5-dimethoxyphenyl)benzo[*d*]oxazol-5-yl)-2-methylbenzamide (**BXZ7**), and *N*-(2-(3,5-dimethoxyphenyl)benzo[*d*]oxazol-5-yl)benzamide (**BXZ8**) were the most potent compounds with 84.09, 79.54, and 70.45% of edema inhibition, respectively, as compared with standard drug ibuprofen (65.09% inhibition). Moreover, computational studies revealed the presence of H-bond with Arg120 and π–π interaction with Tyr355 within the active site of COX-II. In addition, docking score revealed that *o*-substituted and unsubstituted phenyl ring increased the activity. Compounds with electron withdrawing group at *p*-position of phenyl ring exhibited moderated activity, and reduced activity was observed from compounds substitutents with electron donating group at the *p*-position (Figure 19).

Yatam et al.^122^ designed oxadiazole linked benzoxazole derivatives using a scaffold hopping approach and evaluated their molecular level interactions with COX-I/II using *in silico* molecular docking. A series of 2-(((5-aryl-1,2,4-oxadiazol-3-yl)methyl)thio)benzo[*d*]oxazoles were synthesized and assessed *in vitro* for COX inhibition. Some of the compounds exhibited selectivity for COX-II enzyme, and none among them exhibited activity against COX-I. Among all, 2-(((5-(4-nitrophenyl)-1,2,4-oxadiazol-3-yl)methyl)thio)benzo[*d*]oxazole (**BXZ9**) showed excellent potency with 63.67% inhibition against COX-II at 10 μM concentration. Moreover, the *in vivo* anti-inflammatory activity evaluated using the carrageenan induced paw edema method showed maximum anti-inflammatory activity for **BXZ9** with 80.6% inhibition of edema observed along with potent antioxidant activity (Figure 19).

### 4.9 Isatin Derivatives as Anti-inflammatory Agents

Two isoforms, i.e., hCA IX and hCA XII, became the most promising targets in anticancer drug discovery. Therefore, there is a strong need to synthesize new drugs that target hCA IX and XII instead of hCA I and II. Literature studies established isatin as a good scaffold for the development of selective inhibitors for both tumor associated carbonic anhydrase isoforms IX and XII. Some drugs like Sunitinib and Nineredanib having isatin moiety are FDA approved.^123^

**Figure 20:**
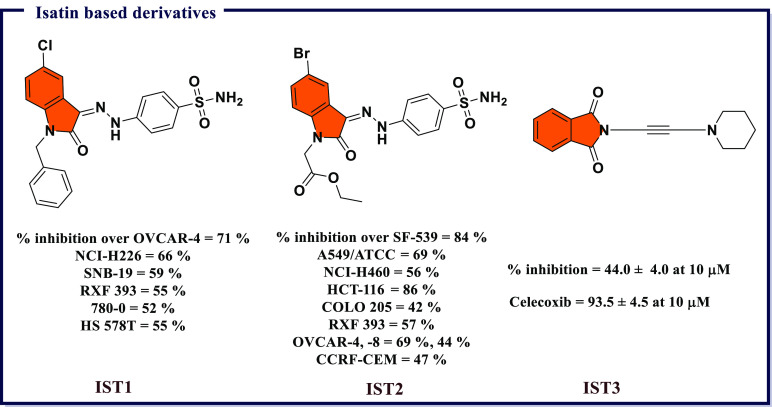
Isatin based derivatives as potential COX-II inhibitors.

Continuing the efforts in this direction, Ashour et al.^124^ synthesized two series of 3-hydrazinoisatin based sulfonamides. All the synthesized sulfonamides were biologically evaluated against hCA I, II, IX, and XII isoforms. The sulfonamides exhibited potent inhibitory activities toward transmembrane tumor-associated hCA isoforms IX and XII with *K*_*i*_ ranging from 8.3–65.4 nM and 11.9–72.9 nM, respectively. Among all, (*Z*)-4-(2-(1-benzyl-5-chloro-2-oxoindolin-3-ylidene)hydrazineyl)benzenesulfonamide (**IST1**) and ethyl (*Z*)-2-(5-bromo-2-oxo-3-(2-(4-sulfamoylphenyl)hydrazineylidene)indolin-1-yl)acetate (**IST2**) exhibited broad spectrum antiproliferative activity toward various cell lines. While both compounds exhibited nonsignificant inhibitory activity toward CDK2 and CDK9, **IST2** noticeably inhibited colony formation in HCT-116 cells in a concentration-dependent manner as compared to untreated control and in a single-digit mM range. Moreover, molecular modeling studies were performed to gain a vision for the possible binding interactions and affinities for the target isatin-based sulfonamides within hCA isoforms II and IX active sites (Figure 20).

Qaisi et al.^125^ synthesized aminoacetylenic isoindoline-1,3-dione derivatives. The evaluation of COX-I/II inhibitory activities of these derivatives showed that several molecules possessed significant anti-inflammatory activity. Among all, 2-(4-(piperidin-1-yl)but-2-yn-1-yl)isoindoline-1,3-dione (**IST3**) was most effective anti-inflammatory agent, even more effective than reference drug diclofenac, ibuprofen, and nearly as effective as Celecoxib (Figure 20).

### 4.10 Coumarin Derivatives as Anti-inflammatory Agent

Coumarins are a special class of flavonoids compounds that exhibit a variety of biological and pharmacological activities. Coumarin and its derivatives also had a beneficial effect on human health as they possess anti-inflammatory, antioxidant, and antibacterial activities. The styryl carbonyl moiety into a rigid framework of coumarin has anti-inflammatory activity. Both coumarin and its derivatives inhibited lipoxygenase (LOX) and cyclooxygenase (COX) pathways. A variety of pharmacophoric groups at different positions (C-3, C-4, and C-7) of coumarin resulted in different biological and synthetic coumarin derivatives.^126^

**Figure 21:**
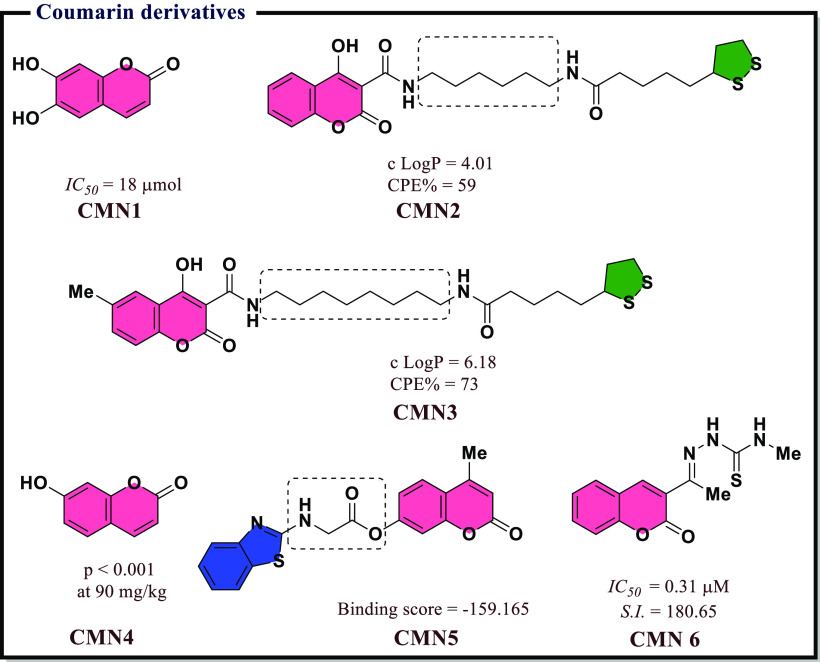
Coumarin derivatives as potential COX-II inhibitors

First, Silvan et al.^127^ isolated four coumarins derivatives from the EtOAc extract of the flower-tops of *Santolina oblongifolia boiss* (Compositae) (**CMN1**) for better inhibition of COX-II. These molecules exhibited good activities as inhibitors of eicosanoid-release from ionophore-stimulated mouse peritoneal macrophages. The 6,7-dihydroxycoumarin (aesculetin) molecule showed a significant activity (*IC*_*50*_ = 18 μmol) with an excellent percentage inhibition similar to the reference drug nordihydroguaiaretic acid (NDGA) (*IC*_*50*_ = 6 μmol). The % inhibition of **CMN1** against PGE_2_-release and LTC_4_-release was 71.73 and 71.20, respectively, with excellent anti-inflammatory activities (Figure 21).

After that, the 4-hydroxycoumarin moiety as a molecular template was utilized for the synthesis of various analogs with good biological activity. Further, Melagrak et al.^128^ utilized this moiety in the synthesis of a series of novel coumarin analogs and evaluated them for *in vivo* and *in vitro* antioxidant activity via inhibiting lipooxygenase and COX-II, respectively. With the help of structure–activity relationship (SAR), *N*-(6-(5-(1,2-dithiolan-3-yl)pentanamido)hexyl)-4-hydroxy2-oxo-2*H*-chromene-3-carboxamide (**CMN2**) and *N*-(8-(5-(1,2-dithiolan-3-yl)pentanamido)octyl)-4-hydroxy6-methyl-2-oxo-2*H*-chromene-3-carboxamide (**CMN3**) were found to be more potent *in vivo* than lipoic acid (c LogP = 2.39, CPE% = 29.6) (Figure 21). Exploring the therapeutic effects of umbelliferone, Vasconcelos et al.^129^ isolated coumarin from *Typha domingensis* and investigated it in a mouse model of bronchial asthma. With the treatment of umbelliferone (**CMN4**), a reduction of cellularity, eosinophil numbers in bronchoalveolar lavage fluids, decrease in mucus production, and lung inflammation at 60 and 90 mg/kg was observed in asthmatic mice. Further, the mechanism of action of **CMN4** helped in the progress of novel drugs for the treatment of asthma (Figure 21).

To develop novel conjugates, Sandhya et al.^130^ synthesized coumarin derivatives using various aromatic and heterocyclic amines. All the synthesized compounds exhibited better anti-inflammatory activities. Most of the compounds exhibited anti-inflammatory activity due to H-bonding with a receptor site. Among all, 4-methyl-2-oxo-2*H*-chromen-7-yl-*N*-1,3-benzothiazole-2-yl-glycinate (**CMN5**) exhibited good activity as compared to standard drugs. Docking studies revealed the presence of excellent binding interaction of **CMN5** with Arg 44 amino acid (binding score −159.165). The result revealed that heterocyclic derivatives seem to be more potent with nitrogen at the 7-position of coumarin. The inhibition value of **CMN5** increases after 1, 2, 3, and 4 h up to 40.6 (Figure 21).

**Figure 22:**
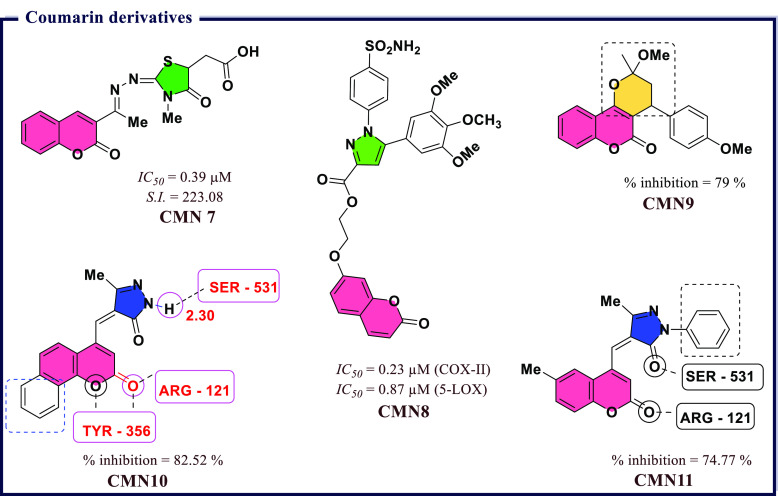
Coumarin derivatives as potential COX-II inhibitors.

Another class of COX inhibition was discussed, which had coumarin ring fused with thiazole and thiazolidinone. Dawood et al.^131^ synthesized two new series of coumarin derivatives having thiazoline and thiazolidinone moieties. Further, the synthesized compounds were evaluated *in vivo* using the carrageenan-induced rat paw edema model and *in vitro* against the human cyclooxygenase. Among all the synthesized compounds, *N*-ethyl-2-(1-(2-oxo-2*H*-chromen-3-yl)ethylidene)-hydrazine-1-carbothioamide (**CMN6**) and 3,5-dimethyl-2-{[1-(2-oxo-2*H*-chromen-3-yl)-ethylidene]-hydrazono}-thiazolidin-4-one (**CMN7**) had better anti-inflammatory activity. *In vivo*, both **CMN6** and **CMN7** displayed excellent anti-inflammatory potential and superior *GI* safety profiles (0–7% ulceration) over Indomethacin. *In vitro* experiment revealed high affinity and selectivity toward COX-II isoenzyme as compared to the standard drug Celecoxib (*IC*_*50*_ values ranging from 0.31 to 0.78 μM). With the help of molecular docking study, the presence of various binding interaction was explored (Figure 21, Figure 22).

Rayar et al.^132^ had reported a sequence of cyclocoumarol derivatives and evaluated them for anti-inflammatory activity using assay of PGE_2_ production. All the synthesized compounds had much more inhibition value than the later compound. Among all, 2-methoxy-2-methyl-(1-(4-methoxyphenyl))-3,4-dihydropyrano[3,2-*c*]chromen-5(2*H*)-one (**CMN9**) was most potent with inhibitory activity (79% inhibition) of PGE_2_ as compared to NS-398. But, none of the compounds exhibited inhibitory activity toward COX-I. With the help of molecular docking studies, the hydrogen bonding between the oxygen of the methoxy group with His90 and Arg513 at the active site of the COX-II enzyme was displayed (Figure 22). Further, the work was extended for the development of better inhibition at low concentration, and novel coumarin was synthesized using different hybrids. Kulkarni et al.^133^ reported a sequence of new coumarin-pyrazolone hybrids by condensing 3-methyl pyrazolone with 4-formyl coumarin. The synthesized molecules were evaluated for *in vitro* anti-inflammatory and anticancer activities. A number of tested molecules exhibited potent anti-inflammatory effect against COX-II enzyme. Among all, 3-methyl-4-((3-oxo-3*H*-benzo[*f*]chromen-1-yl)methylene)-1*H*-pyrazol-5(4*H*)-one (**CMN10**) and 3-methyl-4-((6-methyl-2-oxo-2*H*-chromen-4-yl)methylene)-1-phenyl-1*H-*pyrazol-5(4*H*)-one (**CMN11**) exhibited the most % inhibition of egg albumin in 100 μg/mL with 82.52 and 74.77% inhibition, respectively. Moderate anticancer activity was observed from compound when evaluated using *in vitro* methods. Molecular docking studies revealed that **CMN10** and **CMN11** showed comparatively good interaction with the COX-II enzyme (Figure 22).

### 4.11 Indole Derivatives as Anti-inflammatory Agents

Heterocyclic compounds consisting of a benzene ring fused with the pyrrole ring had various biological applications. Various indole alkaloids and plant growth hormones are well-known in nature for their COX-II inhibition properties. Over the past few years, various chemical compounds consisting of indole nucleus have been synthesized and evaluated in various biological activities.^134^ In the present review, different indole-based derivatives have been discussed as potential COX-II inhibitors.

**Figure 23:**
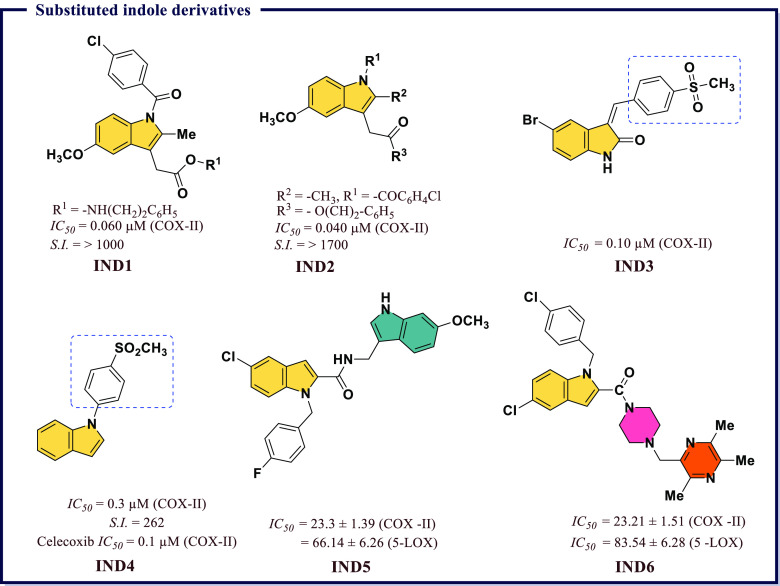
Substituted indole derivatives as potential COX-II inhibitors.

Kalgutkar et al.^135^ developed a series of Indomethacin ester and amide derivatives and evaluated their inhibitory activities against COX-II using carrageenan-induced footpad edema assay. Among them, 1-*p*-chlorobenzoyl-5-methoxy-2-methylindole-3-phenethyl amide (**IND1**) and 1-*p*-chlorobenzoyl-5-methoxy-2-methylindole-3-phenethyl ester (**IND2**) were found to be the better inhibitors of COX-II along with reduced gastrointestinal side effects of parent compound. Among all, primary and secondary amide derivatives were found to be potent inhibitors as compared to tertiary amide derivatives, as the carboxylate-binding region of COX-II includes Tyr355 and Glu524. The different esters and amides derivatives inhibited human COX-II with a lower range of *IC*_*50*_ but did not inhibit ovine COX-I activity at concentrations as high as 66 μM. Substitution of 4-chlorobenzoyl group in esters or amides with the 4-bromobenzyl functionality resulted in loss of compound activity. The amide derivatives behave as slow, tight-binding, and selective inhibitors in the inhibition kinetics (Figure 23). Lai et al.^136^ synthesized and evaluated 14 new 3-[4-(amino/methylsulfonyl)phenyl]methylene-indolin-2-one derivatives for *in vivo* anti-inflammatory activity using the carrageenan-induced paw edema rat model. Structure–activity relationship (SAR) revealed higher inhibitory potential for methylsulfonyl substituted derivatives against COX-I/II and 5-LOX than sulfamoyl substituted derivatives. Most of the compounds exhibited potent inhibitory activity when evaluated for biological analysis. They had also displayed strong analgesic activity using acetic acid-induced writhing assay in mice. It was observed that methylsulfonyl derivatives, i.e., (*Z*)-5-bromo-3-(4-(methylsulfonyl)benzylidene)indolin-2-one (**IND3**), showed maximum inhibitory activity against COX-II with *IC*_*50*_ value of 0.1 μM in comparison to DBF and TND. Better gastric tolerance was observed for **IND3** due to the presence of aminosulfonylphenyl or methylsulfonylphenyl moiety in comparison to standard compounds DBF and TND (Figure 23).

Structure–activity relationship (SAR) revealed higher cytotoxicity for *N*-(2-fluorophenyl)pyrrole subunit as compared to aromatic rings. A series of 4-(aryloyl)phenylmethyl sulfones were synthesized and evaluated for *in vivo* inhibitory activity using the carrageenan rat paw edema assay by Harrak et al.^137^ Among the synthesized *N*-arylindole derivatives, 1-(4-(methylsulfonyl)phenyl)-1*H*-indole (**IND4**) was found to the potent one with excellent *IC*_*50*_ value (0.3 μM) and selectivity index (262). **IND4** exhibited 48.7% of inhibition after 3 h, which was found to be more than the reference drug ibuprofen. *In silico* molecular docking studies revealed that the methylsulfone group of indole derivatives fit snuggly into the binding pocket of human COX-II. The additional information about the most potent compound was obtained with the help of QSAR, i.e., better correlation coefficient (*r*^2^ = 0.808) and dipole moment (Figure 23).

A series of novel indole-2-amides were tested *in vivo* for anti-inflammatory activity using the mice auricle edema model and with dexamethasone as a positive control by Huang et al. But some of these compounds are associated with several side effects with insignificant biological results. Further studies revealed that some of the indole-2-amide derivatives had better COX-II and 5-LOX inhibitory activities as compared to the reference drug Celecoxib. Among all the screened compounds, 1-benzyl-5-chloro-*N*-(2-(5-methoxy-1*H*-indol-3-yl)ethyl)-1*H*-indole-2-carboxamide (**IND5**) and 1-benzyl-5-chloro-1*H*-indol-2-yl)(4-benzylpiperazin-1-yl)methanone (**IND6**) displayed the highest COX-II inhibition at 23.3 nM and 23.21 nM, respectively, with selectivity index of 17.45 and moderate 5-LOX inhibitory activity (*IC*_*50*_ = 66 and 83.54 nM) comparable to positive controlled Zileuton (*IC*_*50*_ = 38.91 nM). The cytotoxic effects were not observed in the normal cells. The molecular docking studies revealed that the most active compound exhibited similar binding modes as exhibited by cocrystallized SC-558 ligand (Figure 23).^138^

**Figure 24:**
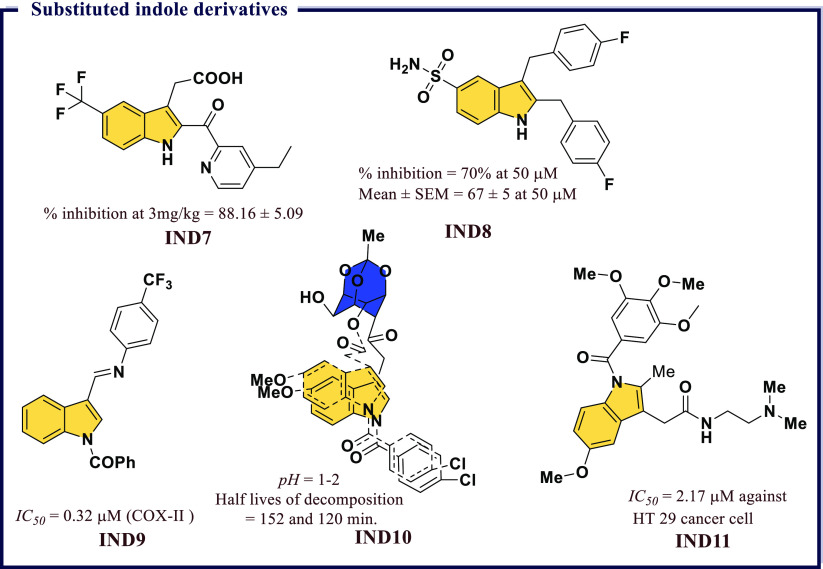
Substituted indole derivatives as potential COX-II inhibitors.

Hayashi et al.^139^ synthesized a series of [2-(6-or-5-substituted)-1*H*-indol-3-yl]acetic acids as a new class of antipyretic and anti-inflammatory drugs. They screened all the compounds for COX-II inhibition using carrageenan rat paw edema assay with the elimination of PGE_2_ in the edema site. Among all acid derivatives, 2-[(4-ethylpyridin-2-yl)carbonyl]-5-(trifluoromethyl)-1*H*-indol-3-yl acetic acid (**IND7**) was found to be most potent with a significant *IC*_*50*_ value (*IC*_*50*_ = 0.00229 mM) against COX-II activity in HUVEC assay and COX-I activity in HWB assays (*IC*_*50*_ = 42.00 μM). The correlation coefficient (*r*^2^ = 0.9819) was found to be very significant between the dose amount and % inhibition. For the oral administration, half-maximal effect (*ED*_*50*_ = 1.68 mg/kg) was observed over a range of doses like 0.3, 1.0, 3.0, and 10 mg/kg with 78.17% inhibition (Figure 24).

Estevão^140^ studied different substitution patterns on the indole scaffold and generated a class of new indolic compounds for further biological activity. The substitution patterns at different positions (C-5, C-3, and N-1) of sulfonamide or methylsulfone have been explored. Among all the substituted indolic derivatives, 2,3-*bis*(4-fluorobenzyl)-1*H*-indole-5-sulfonamide (**IND8**) resulted in 67 ± 6% (50 μM) of COX-II inhibition and 18.2 ± 8.5% of COX-I inhibition, which was close to Celecoxib. Docking studies revealed higher selectivity for the dialkylated compounds (at C-2 and C-3) compared to the monoalkylated ones due to similar binding patterns as observed in SC-558. The inhibition value of these derivatives at 50 μM was found to be 70% for COX-II and low for COX-I (18 ± 9%). Substitution with larger moiety did not exhibit significant activity due to their bulkiness, as they were unable to fit inside the binding pocket. Moreover, different directions of sulfonamide group in the binding pocket were revealed via saturation transfer difference NMR experiments (Figure 24).

In the development of selective COX-II inhibitors, indole ring was selected as a template to design novel indole ring based NSAIDs. In this respect, the Schiff base analogs of Indomethacin possess a safety profile and are selective in nature similar to Celecoxib. Kaur et al.^141^ synthesized *di*-substituted-indole Schiff bases derivatives (N-1, C-3) and evaluated them as inhibitors of cyclooxygenase isozymes (COX-I/COX-II). Out of these derivatives, some compounds identified as effective and selective COX-II inhibitors with excellent *IC*_*50*_ and good selective index (*IC*_*50*_ = 0.32–0.84 μM range, and selectivity index *S.I.* = 113 to >312 range). Especially, 1-benzoyl-3-[(4-trifluoromethylphenylimino)methyl] indole (**IND9**) was found as the most potent (COX-I *IC*_*50*_ > 100 μM; COX-II *IC*_*50*_ = 0.32 μM) and selective (*S.I.* > 312) COX-II inhibitor against the reference drug Indomethacin (COX-I *IC*_*50*_ = 0.13 μM; COX-II *IC*_*50*_ = 6.9 μM, COX-II, *S.I.* = 0.02). Molecular modeling studies revealed that the presence of CF_3_ substituent on indole ring enhanced the biological activities (Figure 24). Kadirvel et al.^142^ developed novel myo-inositol-Indomethacin esters to reduce serious problems of gastrointestinal (*GI*) and colorectal cancer after prolonged exposure. Among all, 4,6-*bis*-*O*-2-[1-(4-chlorobenzoyl)-5-methoxy-2-methyl-1*H*-indol-3-acetyl]-myo-inositol-1,3,5-orthoacetate (**IND10**) had long half-life (152 min), which implied that the compounds may be stable in the stomach with minimal hydrolysis upon oral administration (between pH 4.0–8.5) at 37 °C over 24 h. It was observed that **IND10** had two sterically hindered Indomethacin groups at diaxial position on the myo-inositol ring. Further, it undergoes acid cleavage and results in stable penta-equatorial chair conformation. This compound showed comparable biological activity to both the reference compound and its isopropyl derivatives (Figure 24). The selectivity index or ratio (*S.I.*) indicates the NSAID concentrations required for the COX-I and COX-II inhibition. This is an *in vitro* technique that compares the selectivity of COX inhibitors and that considers the ratio of the *IC*_*50*_ of COX-1 with COX-II. A *S.I.* of 1.0 indicates non selectivity, <1 indicates preferential COX-I inhibitor, while the *S.I.* value of >1 is considered to be COX-II selective.^143^

Sulindac sulfide inhibits the growth of colon tumor cells through the induction of apoptosis using phosphodiesterase (PDE_5_) inhibition. Furthermore, certain limitations are associated with Indomethacin and sulindac when utilized in cancer therapy. Various carboxylic acid functionalized Indomethacin analogs have been developed by Chennamaneni et al.^144^ using structure–activity relationship and explored against colon cancer cells HT29. Among all, *N*-(2-dimethylaminoethyl)-1-(3,4,5-trimethoxybenzoyl)-5-methoxy-2-methyl-1*H*-indole-3-acetamide (**IND11**) with *tri*-methoxy groups exhibited better potency as compared to tubulin polymerization with *IC*_*50*_ around 2.71 μM. The tubulin polymerization inhibited value of the most potent compound **IND11** at 8 and 10 μM was 20 and 56% in 10 min, respectively. However, no inhibition value was noticed at 25 μM, indicating that the active site of tubulin was found to inhibit through the process of saturation. Further, molecular docking studies help to find out the binding mode of this compound in tubulin (Figure 24).

**Figure 25:**
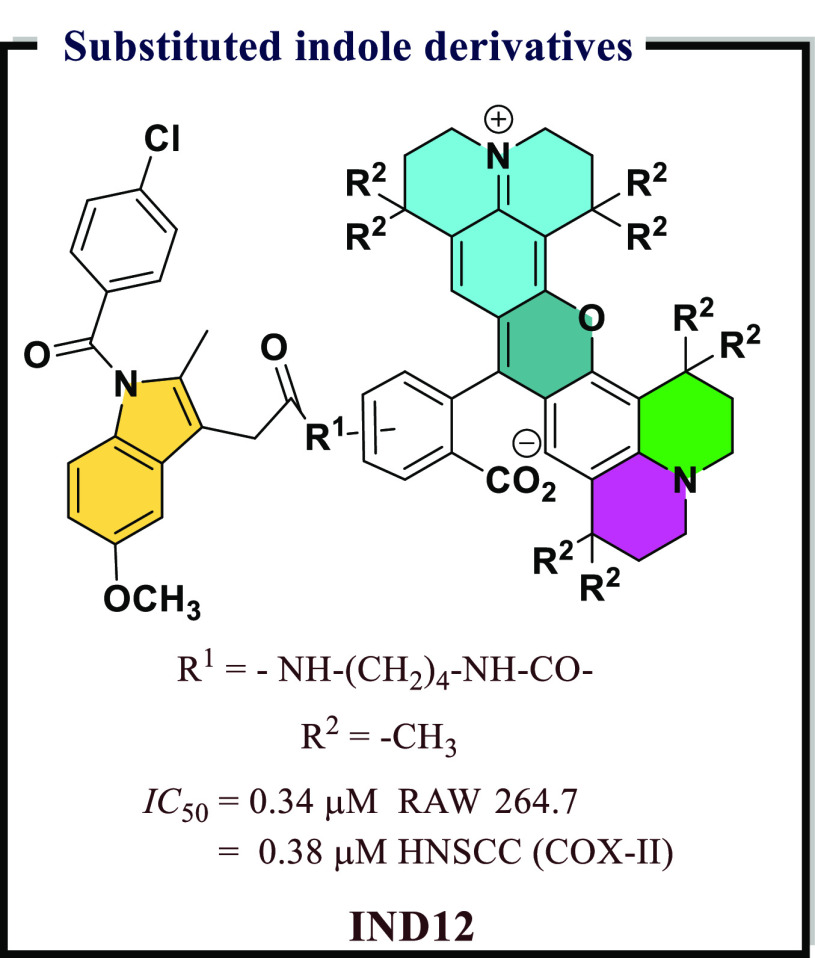
Substituted indole derivatives as potential COX-II inhibitors.

Uddin et al.^145^ utilized structure–activity relationship and developed a wide range of effective and targeted optical imaging agents by conjugation of Indomethacin with carboxy-X-rhodamine dyes to detect COX-II inhibition in inflammatory tissues and premalignant and malignant tumors. Tethering of fluorescent functional groups onto NSAIDs or COXIBs leads to dual-function fluorescent COX-II inhibitors. Further, these compounds were evaluated *in vitro* and *in vivo* as COX-II targeted agents in cells and tumors. In order to develop selective COX-II binding fluorescent probes, a four-carbon *n*-alkyl linker was conjugated with bulky zwitterionic fluorescent functionalities such as ROX. Fluorophores like metal or halide salts and highly polar organic poly(carboxylic acid)s were not suitable for developing COX-II targeted imaging agents. Further, a dramatic reduction in the inhibitory potency was observed with an increase in alkyl chain length. Among all synthesized compounds, the fluorescent conjugates containing bulkier rhodamine dye (**IND12**) have significant *IC*_*50*_ (0.34 μM, 0.38 μM, respectively) values in RAW 264.7 and in 1483 HNSCC cells against COX-II, respectively. A very high degree of selectivity in tissues and tumors was observed in these compounds (Figure 25).

**Figure 26:**
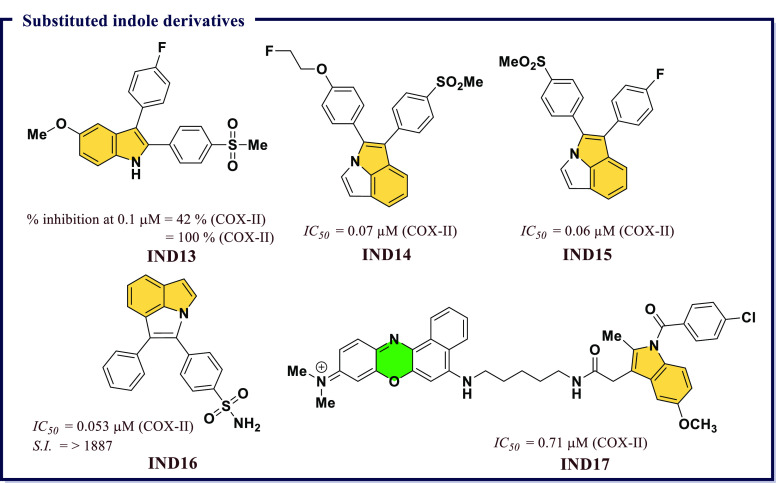
Substituted indole derivatives as potential COX-II inhibitors.

Antioxidative properties of a compound govern its biological activity. Compounds with promising antioxidant properties were found to possess a diverse range of biological activities. Indole is one established class of heterocyclic compounds which possess significant antioxidant response. Considering this fact, Laube et al.^15^ synthesized a class of 2,3-diarylindoles substituted with fluorine or methoxy group and evaluated their inhibitory activity using fluorescence-based and enzyme immunoassay-based assay. Most of the compounds possess autofluorescent properties both *in vitro* and *in vivo* with an emission maximum (443–492 nm), and inhibit COX-II enzyme in micromolar range (0.1 μM). The redox activities of the synthesized compounds were also determined. Among them, 3-(4-fluorophenyl)-5-methoxy-2-[4-(methylsulfonyl)phenyl]-1*H-*indole (**IND13**) was found to be most potent with excellent % inhibition at 0.1 μM (Figure 26).

Continuing their work in this field, Laube et al.^146^ further extended the scope of their work by developing a series of diarylsubstituted heterocycles based on tricyclic dihydropyrrolo[3,2,1-*hi*]indole and pyrrolo[3,2,1-*hi*]indoles. The synthesized compounds were evaluated for COX-II inhibition and exhibited excellent COX-II inhibition with *IC*_*50*_ ranging from 20 to 2500 nM. Among all, 4-[4-(2-fluoroethoxy)phenyl]-5-[4-(methylsulfonyl)phenyl]-1,2-dihydropyrrolo[3,2,1-*hi*]indole (**IND14**) and 1-(4-fluorophenyl)-2-[4-(methylsulfonyl)phenyl]pyrrolo[3,2,1-*hi*]indole (**IND15**) exhibited selective COX-II inhibitory activity with excellent *IC*_*50*_ value of 70 nM and 60 nM, respectively. *In silico* molecular docking studies were performed to predict the probable mode of binding in the active site of COX-II. The results revealed that fluorine substituent could be a promising candidate for the development of ^18^F radiolabeled COX-II inhibitors and can be utilized in positron emission tomography (PET) (Figure 26).

Previous studies revealed that the specific site for COX-II inhibition should have low metabolic stability, fast excretion from the body, and high lipophilicity. It was observed with the help of positron emission tomography that COX-II inhibitors were used for noninvasive imaging due to their significant biological importance. A second series of sulfonamide-substituted dihydropyrrolo[3,2,1-*hi*]indoles were developed and evaluated by Laube et al.^147^ Further, these derivatives were transformed into more hydrophilic *N*-propionamide-substituted derivatives. Sulfonamide-substituted compounds have better potency and selectivity than methylsulfonyl-substituted derivatives. A significant decrease in lipophilicity was observed in *N*-propionamide-substituted analogs without COX-II inhibition potency. The pyrrolo[3,2,1-*hi*]indoles derivatives were found to be more potent as well as highly selective inhibitors for COX-II with *IC*_*50*_ in a narrow microrange (0.053–0.092 μM). Among all, 1-phenyl-2-[4-(sulfamoyl)phenyl]pyrrolo[3,2,1-*hi*]indole (**IND16**) had better inhibition value (*IC*_*50*_ = 53 nM) for COX-II. Hence, the sulfonyl propionamides derivatives can be regarded as potential prodrugs for development of more refined radiotracers (Figure 26).

Near-infrared (NIR) based imaging agents have been extensively used for *in vivo* detection of cancer sites as they have minimal photodamage to biological sample, minimal interference from background autofluorescence, and acceptable to the fluorescent light through biological tissues. Wang et al.^148^ designed the first golgi-localized cyclooxygenase-II (COX-II)-specific near-infrared (NIR) based fluorescent probe (Niblue-C6-IMC able) (**IND17**), which exhibited high tissue penetration capacity in tumors. The binding affinity of this compound displayed excellent inhibition of COX-II (*IC*_*50*_ = 0.71 nM) as compared to IMC (*IC*_*50*_ = 0.75 nM). Hence, these data confirmed that these fluorescent probes serve as potent and COX-II-selective inhibitors in cancer cells (Figure 26).

**Figure 27:**
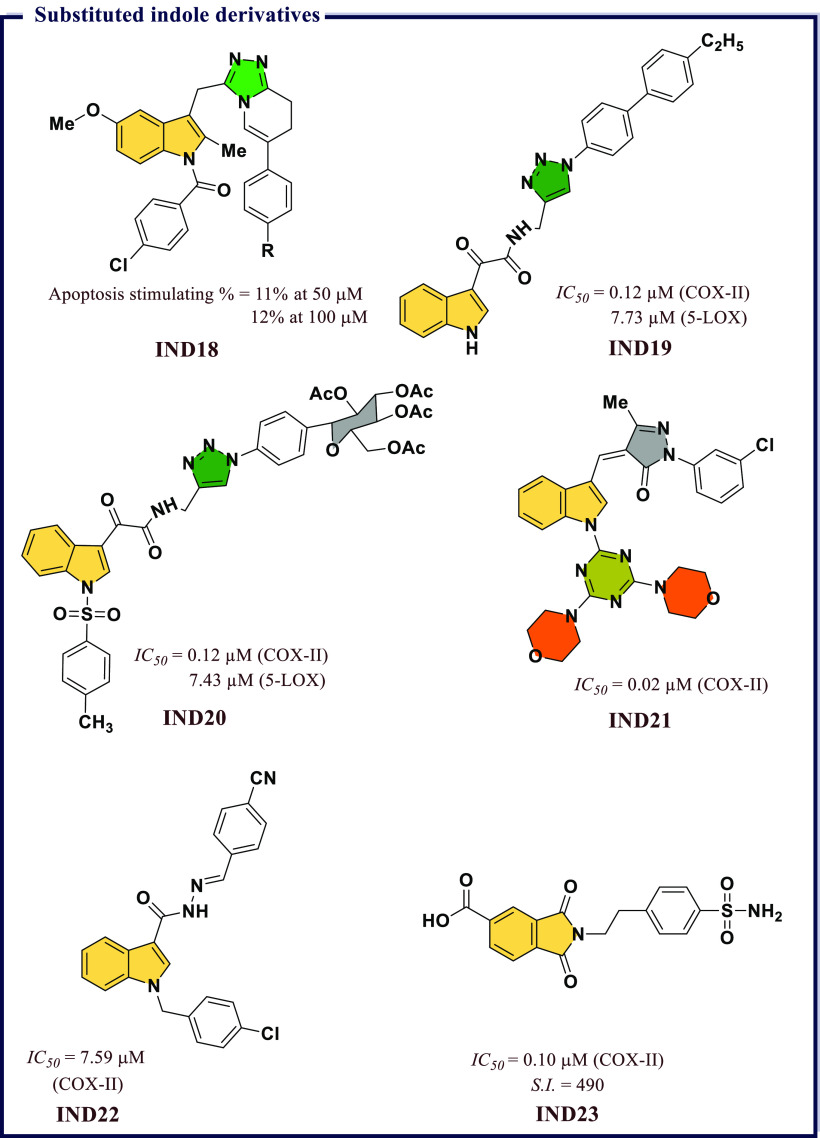
Substituted indole derivatives as potential COX-II inhibitors.

1,2,4-Triazole has established itself as a privileged scaffold and gained significant potency in the area of cancer research. Considering this fact, Sever et al.^149^ synthesized a new series of 1,2,4-triazolo[3,4-*b*]-1,3,4-thiadiazines and evaluated them against T98 human glioma cell line. For the inhibitory effect of different derivatives, the MTT assay was carried out on the proliferation of T98 human glioma cell line. Among all, 3-[5-methoxy-2-methyl-1-(4-chlorobenzoyl)-1*H*-indole-3-yl)methyl]-6-(4-methylphenyl)-7*H*-1,2,4-triazolo[3,4-*b*]-1,3,4-thiadiazine (**IND18**) exhibited 25% and 40% inhibitory activity at 50 and 100 μM, respectively. Moreover, the apoptosis stimulating percentage was 11% and 12%, respectively. *In silico* docking studies revealed that the dose-dependent catalytic active site of COX-II was similar to Indomethacin (Figure 27).

Naaz et al.^150^ have developed two series of 1,2,3-tethered indole-3-glyoxamides to control cancer cell proliferation and gastric ulceration. The derivatives were evaluated for COX-II inhibition using carrageenan-induced hind paw edema model. Among all, *N*-((1-(4-ethylphenyl)-1*H*-1,2,3-triazole-4-yl)methyl)-2-(1*H-*indol-3-yl)-2-oxoacetamide (**IND19**) and (2*S*,4*R*,5*S*)-2-(acetoxymethyl)-6-(4-((2-oxo-2-(1-tosyl-1*H-*indol-3-yl)acetamido)methyl)-1*H*-1,2,3-triazol-1-yl)tetrahydro-2*H*-pyran-3,4,5-triyltriacetate (**IND20**) exhibited good COX-II inhibition (*IC*_*50*_ = 0.12 μM) and better selectivity index over COX-I (0.058 and 0.046). These compounds also displayed 5-LOX inhibitory activity (*IC*_*50*_ = 7.73 and 7.43 μM) in comparison to nordihydroguaiaretic acid (*IC*_*50*_ = 7.31 μM). **IND19** displayed excellent antiproliferative activity against DU145 prostate cancer line. *In vitro* tubulin assay revealed that **IND19** acts as tubulin polymerization inhibitor via obstruction of with microtubulin dynamic. Molecular docking studies suggested good binding affinity toward COX-II and 5-LOX and also revealed that both compounds occupy the colchicines binding site of tubulin polymer (Figure 27).

Kaur et al.^151^ developed a library of hybrid molecules by combining triazin-indole adduct with different active moieties and then screened them for anti-inflammatory activity using enzyme immunoassays and animal models. Among all, (4*Z*)-1-(3-chlorophenyl)-3-methyl-4-((1-(4,6-dimorpholino-1,3,5-triazin-2-yl)-1*H*-indol-3-yl)methylene)-1*H-*pyrazol-5(4*H*)-one (**IND21**) was found to be the most potent with an *IC*_*50*_ value 0.02 μM, which was 2-times higher than that of Celecoxib and was comparable to that of diclofenac. Good selectivity index for COX-II (*S.I.* = 150) along with better activity compared with Indomethacin and diclofenac was observed. The molecule also exhibited consistent anti-inflammatory activity both *in vivo* and *in vitro*. Mechanistic studies on Swiss albino mice revealed better selectivity for COX-II with minimum toxicity. Molecular docking and QSAR studies were found to be in good correlation with the results of solution-phase NMR experiments (Figure 27).

Recently, the overexpression of iNOS, COX-II, TNF-α, and IL-6 was observed in LPS-stimulated murine RAW264.7 macrophages, which was suppressed by indolyl-*N*-substituted benzyl/benzoyl derivatives. Hence, Ju et al.^152^ synthesized indole derivative and evaluated them *in vitro* for activity to inhibit COX-II with improved safety profile using an enzyme immunoassay (EIA). Among all, (*E*)-1-(4-chlorobenzyl)-*N*′-(4-cyanobenzylidene)-1*H*-indole-3-carbohydrazide (**IND22**) exhibited COX-II inhibitory activity (*IC*_*50*_ = 7.59 μM) close to that of Celecoxib (*IC*_*50*_ = 1.31 μM) and DUP-697 (*IC*_*50*_ = 0.02 μM), which led to the suppression of ROS. Further, molecular docking studies revealed that **IND22** had a lack of binding affinity along with the nonsignificant selectivity toward COX-II (Figure 27).

Aziz et al.^153^ synthesized trimellitimide derivatives using sulfamoyl, carboxylate carboxylic acid, and methyl carboxylate moieties and tested them *in vivo* using the rat carrageenan-induced foot paw edema model for anti-inflammatory effects. Most of the compounds with sulfonamide and carboxylic acid moieties exhibited selective COX inhibition with a selectivity index ranging from 265.7 to 490. The sulfonamide moieties had negligible ulcerogenic activity. In this case, the selectivity index (*S.I.* > 200–490) range was comparable to that of Celecoxib [COX-II (*S.I.*) > 416.7]. Among all, 1,3-dioxo-2-(4-sulfamoylphenethyl) isoindoline-5-carboxylic acid (**IND23**) was found to be extremely potent COX-II/CA inhibitors (*IC*_*50*_ = 0.10 μM, *K*_*i*_ = 348.3 nM and *S.I.* = 490) as compared with Celecoxib (*IC*_*50*_ = 0.12 μM, *S.I.* > 416). These compounds were found to be suitable candidates for preclinical evaluation in the treatment of various diseases, i.e., glaucoma and other eye diseases (Figure 27).

### 4.12 Quinoline and Isoquinoline Derivative as Anti-inflammatory Agents

Inflammation is the primary response of the immune system toward harmful stimuli such as infection and irritation. Moreover, inflammation leads to acute, chronic, and systemic inflammatory disorders such as cardiovascular disease, autoimmune disease, periodontal disease, and Alzheimer’^`^s disease along with asthma and diabetes. Quinoline and its derivatives attract huge attention from researchers as they can target several causes of inflammation. These compounds inhibit the action of cyclooxygenase-II (COX-II), phosphodiesterase 4 (PDE_4_), tumor necrosis factor (TNF)-α converting enzyme (TACE), as well as transient receptor potential and vaniloid 1 (TRPV 1) antagonists. Several physiological and biological activities are related to quinoline and its derivatives.^154−157^ Dual inhibition of COX/5-LOX had excellent anti-inflammatory activities with lower side effects.^158^ It was found that quinoline and its derivatives bearing substituted moiety resulted in increased activity and efficiency of synthesized compounds.

**Figure 28:**
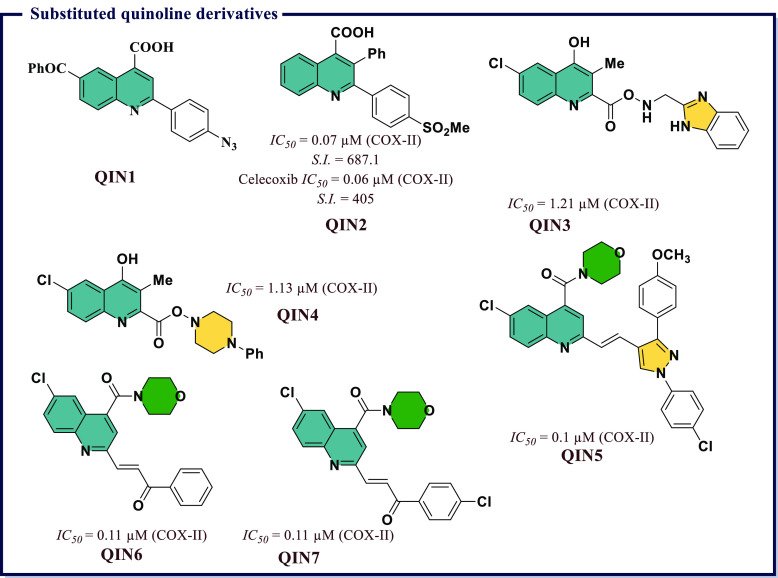
Substituted quinoline derivatives as potential COX-II inhibitors.

Zarghi et al.^159^ reported a novel series of ketoprofen and screened them for COX-II inhibitory activity. All the synthesized compounds had a significant activity and selectivity when evaluated *in vitro* against COX-II with an *IC*_*50*_ value of 0.057–0.085 μM. Among all, 2-(4-(azido)phenyl)-6-benzoyl-quinoline-4-carboxylic acid (**QIN1**) exhibited the highest selectivity and inhibitory activity against COX-II as compared to standard drug Celecoxib. *In silico* molecular docking studies revealed that **QIN1** fits snuggly into the secondary pocket of the active site of COX-II and interacts with Arg513 (Figure 28).

Further, in the same year, a series of novel 2,3-diarylquinolines substituted with methylsulfonyl were synthesized by Ghodsi et al.^160^ and evaluated for anti-inhibitory activity against COX-II. Among all the synthesized compounds, 2-(4-(methylsulfonyl) phenyl)-3-phenylquinoline-4-carboxylic acid (**QIN2**) with *IC*_*50*_ value of 0.07 μM, and selectivity index 687.1, exhibited high selectivity and potent inhibitory activity against COX-II. The inhibitory values of all the synthesized compounds were observed to be more than that of the reference drug Celecoxib (*IC*_*50*_ = 0.06 μM, *S.I.* = 405). With the help of molecular docking studies, it was found that the *p*-methylsulfonyl group on the phenyl ring at C-2 position and carboxylic group displayed better interaction with Ser530 positioned near the COX-II secondary pocket. Furthermore, the structure–activity data indicated that the C-4 quinoline substituted derivatives have better inhibition value (Figure 28).

Abdelrahman et al.^161^ synthesized a series of quinoline-2-carboxamides and evaluated them *in vitro* for COXs/LOX inhibiting activities. Among all, *N*-((1*H*-benzo[*d*]imidazol-2-yl)methyl)-6-chloro-4-hydroxy3-methylquinoline-2-carboxamide (**QIN3**) and (6-chloro-4-hydroxy-3-methylquinolin-2-yl) (4-phenylpiperazin-1-yl)methanone (**QIN4**) displayed excellent selectivity and inhibitory activities against COX-II with *IC*_*50*_ value of 1.21 μM and 1.13 μM as compared to reference drug Celecoxib (*IC*_*50*_ against COX-II = 0.88 μM), respectively. The anti-inflammatory activities of all the synthesized quinoline derivatives were evaluated *in vivo* using the standard carrageenan induced paw edema assay. Both active compounds, **QIN3** and **QIN4**, exhibited % inhibition of 59.38 and 65.03, respectively against COX-II, and also had good gastric safety profile in comparison to Indomethacin. Similar binding patterns as observed in case of cocrystallized ligand bromocelecoxib were observed when both the compounds were evaluated *in silico* using molecular docking studies. The above findings suggest that these derivatives act as a lead compound for further development of molecules with good anti-inflammatory activities and least ulcerogenic index (Figure 28).

A new series of quinolines comprising pyrazole ring with different amide linkages were synthesized by Chaaban et al.^162^ and evaluated for anti-inflammatory activity. Among all, 6-chloro-2-[2-(1-(4-chlorophenyl)-3-(4-methoxyphenyl)-1*H*-pyrazol-4-yl)ethenyl]-4-(morpholin-4-ylcarbonyl)quinoline (**QIN5**), 3-[6-chloro-4-(morpholin-4-ylcarbonyl) quinolin-2-yl]-1-phenylprop-2-en-1-one (**QIN6**), and 3-[6-chloro-4-(morpholin-4-ylcarbonyl)quinolin-2-yl]-1-(4-chlorophenyl)prop-2-en-1-one (**QIN7**) exhibited the most potent inhibition of COX-II with *IC*_*50*_ values 0.1, 0.11, and 0.11 μM, respectively. The ulcerogenic activity of the synthesized compounds was also evaluated, but none of them exhibited significant ulcerogenic activity in comparison to Celecoxib. In addition to COX-II inhibition, these compounds exhibited *in vitro* LOX inhibitory activity higher than that of zileuton. *In silico* molecular docking studies revealed good binding affinity of **QIN5**, **QIN6**, and **QIN7** toward COX-II and proved higher selectivity for COX-II over COX-I (Figure 28).

### 4.13 Tetrazole and Triazole Derivatives as Anti-inflammatory Agents

1,2,4-Triazoles and their heterocyclic derivatives had different biological properties such as antibacterial, antifungal, antitubercular, antiviral, analgesic, anti-inflammatory, anticonvulsant, antidepressant, anticancer, antihypertensive, hypoglycemic, insecticidal, and plant growth activities.^163^

**Figure 29:**
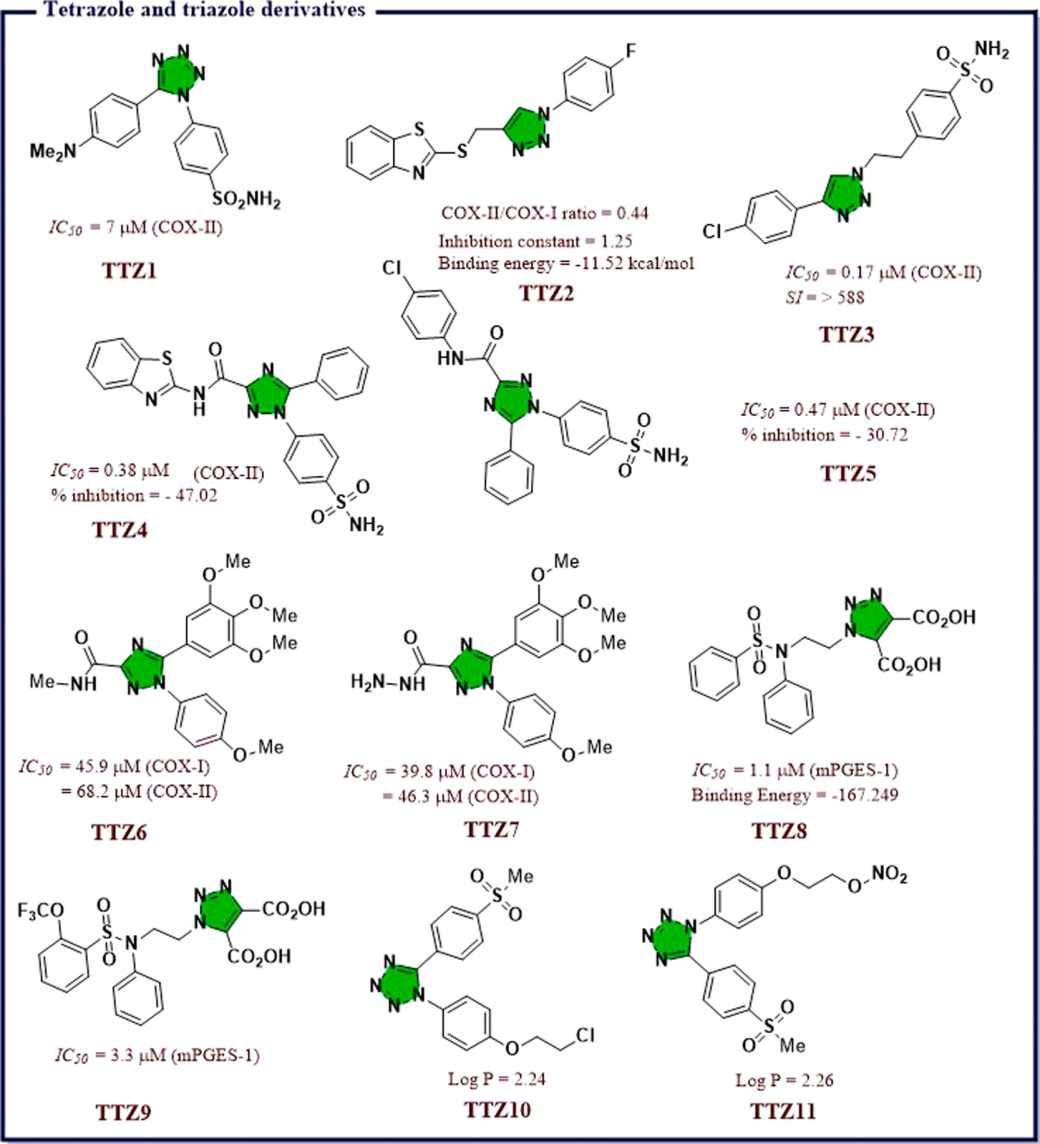
Tetrazole and triazole derivatives as potential COX-II inhibitors.

Hourani et al.^164^ synthesized a series of novel 5-substituted-1*H*-tetrazoles derivatives and evaluated them for *in vitro* COX (COX-I/COX-II) inhibition. Most of the synthesized compounds exhibited anti-inflammatory activities against COX-II. Various diaryl amides with tetrachlorosilane/sodium azide had good inhibitory activity. All the synthesized compounds had better selectivity for COX-II. All the synthesized compounds with methylsulfonyl or sulfonamide group acted as better COX-II pharmacophores and had low inhibitory potency. Among them, the most potent compound tetrazole derivative **TTZ1** exhibited good anti-inflammatory activity with an *IC*_*50*_ value of 7 μM for COX-II. Along with this, **TTZ1** had a better COX-I inhibition with an *IC*_*50*_ value greater than 100 μM (Figure 29).

Further, Shafi et al.^165^ synthesized a library of novel *bis*-heterocycles encompassing 2-mercapto benzothiazole and 1,2,3-triazole using a green approach. The anti-inflammatory activity was evaluated using both the biochemical cyclooxygenase (COX) activity assays and the carrageenan-induced hind paw edema model. Among the tested compounds, 2-((1-(4-fluorophenyl)-1*H*-1,2,3-triazol-4-yl)methylthio)benzo[*d*]thiazole (**TTZ2**) was the most potent and selective COX-II inhibitor with a selective ratio of 0.44. Many of the synthesized compounds exhibited comparable activity to that of standard Ibuprofen, and **TTZ2** was found to possess the most significant anti-inflammatory activity. Further, none of the tested compounds caused gastric ulceration (Figure 29).

A novel group of 1,4-diaryl-substituted triazoles were synthesized by Kaur et al.^166^ and evaluated in COX-II enzyme essay. Significant enzyme inhibition activity was observed increasing the size of an alkyl linker chain [(−CH_2_)_*n*_, where *n* = 0, 1, and 2], and the potency and selectivity of the compounds upon COX-I/COX-II was studied. Further, the tested compounds were screened for *in vitro* inhibition against COX-II isozyme (*IC*_*50*_ = 0.17–28.0 μM range) compared to COX-I isozyme (*IC*_*50*_ = 21.0 to >100 μM range). Among all, 4-{2-[4-(4-chloro-phenyl)-[1,2,3]triazol-1-yl]-ethyl}-benzenesulfonamide (**TTZ3**) displayed the highest COX-II inhibitory potency and selectivity (COX-I: *IC*_*50*_ > 100 μM, COX-II: *IC*_*50*_ = 0.17 μM, *S.I.* > 588). With the help of molecular docking studies, it was found that **TTZ3** had better interaction in the secondary pocket of COX-II active site with the nitrogen atom of the SO_2_NH_2_ (Figure 29)_._

Rahma et al.^167^ synthesized a novel series of 1,2,4-triazole derivatives and evaluated them for anti-inflammatory activity. These synthesized compounds had significant activity against COX-II as compared to Indomethacin and Celecoxib after 3 h. Some of the newly developed compounds exhibited excellent selectivity (ranging from 62.5 to 2127). Among all, *N*-(4-chlorophenyl)-1-[4-(aminosulfonyl)phenyl]-5-phenyl-1*H*-1,2,4-triazole-3-carboxamide (**TTZ4**) exhibited the highest inhibitory potency with *IC*_*50*_ of 0.38 μM and *N*-(benzothiazol-2-yl)-1-[4-(aminosulfonyl)phenyl]-5-phenyl-1*H*-1,2,4-triazole-3-carboxamide (**TTZ5**) exhibited an *IC*_*50*_ value of 0.47 μM with scoring value of 30.72. Further, the molecular docking analyses of the molecules revealed lower CDOCKER energies for the compounds that exhibited higher selectivity *in vitro*. Among them, most of the synthesized compounds had significant anti-inflammatory activity with lesser gastric ulceration as compared to Indomethacin and Celecoxib (Figure 29).

A series of novel 1-(4-methoxyphenyl)-5-(3,4,5-trimethoxyphenyl)-1*H*-1,2,4-triazole-3-carboxamides were synthesized by Aziz et al.^168^ and evaluated in anti-inflammatory activity. These synthesized compounds had excellent anti-inflammatory activity compared to traditional drugs such as Indomethacin and Celecoxib. All the synthesized compounds had lesser gastric ulceration compared to Indomethacin. Among all, 1-(4-methoxyphenyl)-5-(3,4,5-trimethoxyphenyl)-1*H*-[1,2,4] triazole-3-carboxylic acid methylamide (**TTZ6**) exhibited *IC*_*50*_ values of had 45.9 μM and 68.2 μM against COX-I and COX-II, respectively. Similarly, 1-(4-methoxyphenyl)-5-(3,4,5-trimethoxy-phenyl)-1*H*-[1,2,4]triazole-3-carboxylic acid hydrazide (**TTZ7**) exhibited *IC*_*50*_ values of 39.8 and 46.3 μM against COX-I and COX-II. The hydrazide derivative **TTZ7** had lesser gastric toxicity (*U.I.* = 2) compared to standard drugs. The binding mode of **TTZ7** within the active site of COX-II was obtained using molecular docking approach (Figure 29). After that, with the help of fragment-based virtual screening, a series of sulfonamido-1,2,3-triazole-4,5-dicarboxylic derivatives as a novel class of mPGES-1 inhibitors was identified by Lee et al.^169^ This combined computational and experimental studies have led to identification of novel mPGES-1 inhibitor with novel scaffold, 1-[2-(*N*-phenylbenzenesulfonamido)ethyl]-1*H*-1,2,3-triazole-4,5-dicarboxylic acid(**TTZ8**) which was active than MK886 with mPGES-1 selectivity over COX-1 with *IC*_50_ 1.1 μM. Also, the compound showed *IC*_50_ of 3.3 μM against mPGES-1. Thus, compound **TTZ8** would be regarded as a partial nuisance inhibitor of mPGES-1 with a novel scaffold for the optimal design of more potent mPGES-1 inhibitors (Figure 29).

In recent years, Hourania et al.^170^ synthesized the nitric oxide releasing tetrazole. Both tetrazoles, 1-[4-(2-chloroethoxy)phenyl)-5-(4-(methylsulfonyl)phenyl]-1*H*-tetrazole (**TTZ10**) and 2-[4-(5-(4-(methylsulfonyl)phenyl)-1*H*-tetrazol-1-yl)phenoxy]ethyl nitrate (**TTZ11**), displayed significant inhibitory activity. The intermolecular interactions between adjacent molecules had dominated by weak (2.3–2.7 Å) C–H···O and C–H···N contacts. With the help of molecular docking studies, it was revealed that **TTZ10** and **TTZ11** had excellent binding interaction within the active site of the cyclooxygenase-II enzyme. But no inhibition potency was observed in the *in vitro* experiment. The compounds **TTZ10** and **TTZ11** had potent Log P (Log P = 2.24, and Log P = 2.26, respectively) compared to Celecoxib (Log P = 3.01). With the nitric oxide releasing feature, **TTZ11** had better selectivity and inhibition potency toward both COX-I and COX-II isoforms (Figure 29).

### 4.14 Six Membered Excluded Pyridine

**Figure 30:**
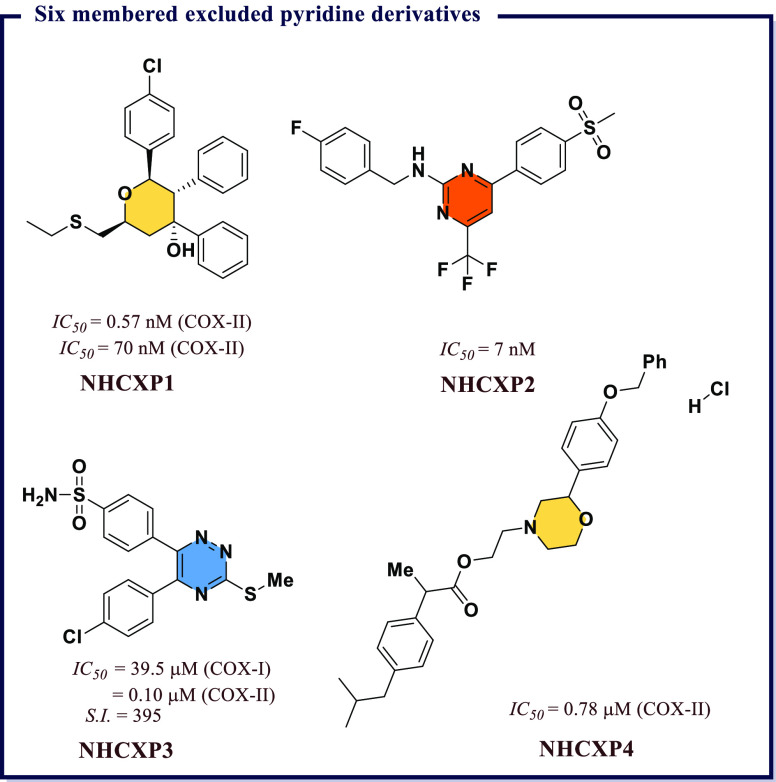
Six membered excluded pyridine derivatives as potential COX-II inhibitors.

The adverse effects of conventional NSAIDs (Rofecoxib and Valdecoxib) included the failure of heart action, *etc*. As a result of this, some fused heterocyclic ring containing compounds other than pyridine were also used in place of commonly used drugs. These compounds had relevant biological activity against COX-I/COX-II. Hence, Singh et al.^171^ synthesized *mono*-, *di*-, and *tri*-aryl substituted tetrahydropyrans by the allylation of beta-hydroxy ketone followed by iodocyclization. These compounds also had significant activity against tumor cells. Most of the synthesized compounds containing triaryl moiety exhibited the highest inhibition for COX-II with *IC*_*50*_ values ranging between 0.57 and 4.0 nM. These compounds also exhibited selectivity index in the range of 3200–44000 for COX-II over COX-I. Among all the derivatives, (2R*,3S*,4R*,6S*)-2-(4-chlorophenyl)-6-((ethylthio)methyl)-tetrahydro-3,4-diphenyl-2*H*-pyran-4-ol (**NHCXP1**) with *IC*_*50*_ value of 0.57 nM and selective index 18771 had activities against COX-II. **NHCXP1** also associated with a lesser amount of gastrointestinal effect (*GI*_*50*_ = 1.7). With the help of a docking study, it was found that **NHCXP1** had better binding interaction with the ligand. Hence, a new class of *tri*- and *di*-aryl-substituted THPs exhibited *GI*_50_ in the range 1.6–3.2 μM over all the human cancer cell lines (Figure 30).

Over the past decade, several COX-II inhibitors compounds were radiolabeled with ^11^C, ^18^F, ^99m^Tc, ^123^I, and ^125^I to evaluate COX-II expression *in vivo* using positron emission tomography (PET) and single-photon emission computed tomography (SPECT). But due to insufficient binding sites, these compounds did not gain much attention. After that, a series of trifluoromethyl-substituted pyrimidines were synthesized by Tietz et al.^172^ as a novel class of selective COX-II inhibitors and evaluated for better inhibitory potency or selectivity *in vitro* against cyclooxygenase (COX-I and COX-II). The biological structure–activity relationship data of three highly potent and selective fluorobenzyl-containing COX-II inhibitors provided theoretical support with the help of molecular docking studies. Radiotracers ^18^F were radiolabeled using 4-[^18^F]fluorobenzylamine ([^18^F]FBA) as a building block. These radiotracers undergo nucleophilic aromatic substitution with radiofluorination without adding fluoride carrier. Among all these radiotracer compounds, *N*-(4-fluorobenzyl)-4-[4-(methylsulfonyl)-phenyl]-6-(trifluoro-methyl)-pyrimidin-2-amine (**NHCXP2**) exhibited better activities against COX-II with an *IC*_*50*_ value of 7 nM (Figure 30).

Due to the hazardous effect of well-known nonsteroidal anti-inflammatory drugs (NSAIDs), a series of 5-aryl-6-(4-methylsulfonyl)-3-(metylthio)-1,2,4-triazine derivatives were synthesized by Irannejad et al.^173^ and evaluated *in vivo* against COX-I/COX-II. All the synthesized compounds had more significant inhibitory activity against COX enzyme with *IC*_*50*_ values in the range of 0.1–0.2 μM than Indomethacin at doses of 3 and 6 mg/kg. Among them, 5-(4-chlorophenyl)-6-(4-(methylsulfonyl) phenyl)-3-(methylthio)-1,2,4-triazine (**NHCXP3**) was the most potent and selective COX-II inhibitor with an *IC*_*50*_ value of 0.10 μM and better selectivity index (*S.I.* = 395) comparable to Celecoxib (*S.I.* = 405). With the help of molecular docking studies, it was found that **NHCXP3** had better binding interaction with COX-II. At last, it was concluded that new analogues of diaryltriazine act as COX-II inhibitors when evaluated under *in vitro* and *in viv*o conditions (Figure 30).

Dou et al.^174^ synthesized a series of novel 2-(2-arylmorpholino)ethyl esters and screened them for inhibitory activity. With the help of structure activity relationship (SAR), the dual COX-II and serotonin reuptake inhibitors of 2-(2-arylmorpholino)ethyl esters of ibuprofen hydrochlorides were determined. Most of the compounds possessed good COX-II selectivity with a better selective index (*S.I.* = 42.8–158.1). Among all, 2-[2-(4-benzyloxyphenyl)morpholino]ethyl-2-(4-iso-butylphenyl)-propanoate hydrochloride (**NHCXP4**) showed better COX-II inhibitory activity (*IC*_*50*_ = 0.78 μM) than Ibuprofen (*IC*_*50*_ = 7.6 μM) and possessed favorable serotonin reuptake inhibitor activity. At the same time, these compounds possessed favorable antidepressant activity compared with Fluoxetine. These compounds can be utilized as basic framework for further development of more effective drugs (Figure 30).

**Figure 31:**
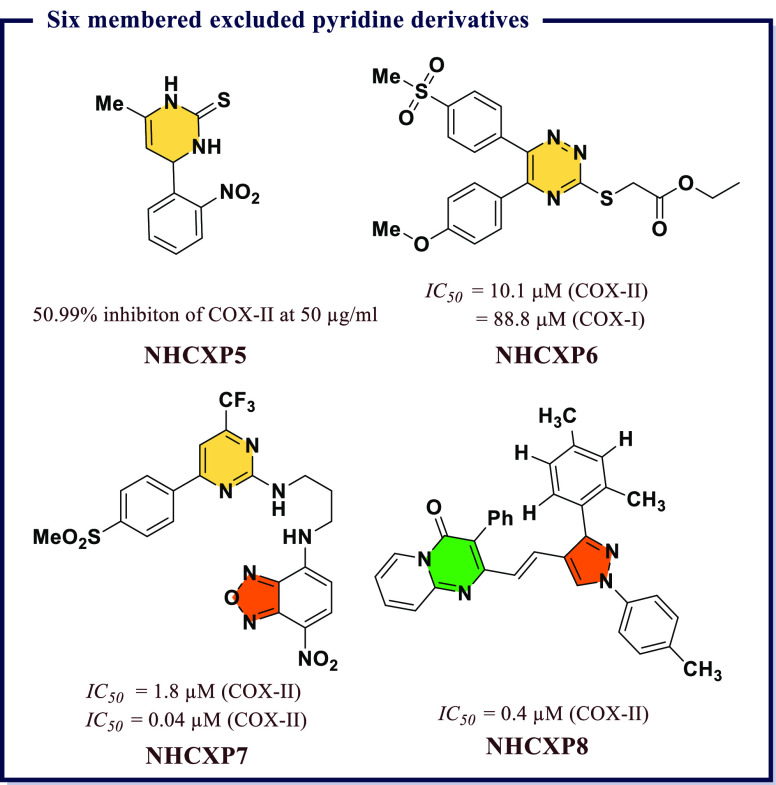
Six membered excluded pyridine derivatives as potential COX-II inhibitors.

A series of novel 4-phenylpyrimidine-2(1*H*)-thiones were reported by Seebacher et al.^175^ to inhibit COX enzyme (COX-I and COX-II) and COX-II expression in THP-1 cell. ADME properties revealed that the synthesized compounds behaved as drugs and further tested for COX-I/II inhibiting activity. However, most of the compounds did not exhibit the inhibitory activity. The synthesized compounds with 4-methoxy and 4-nitro groups acted as better COX-II inhibitors. Among all, (4*RS*)-(±)-3,4-dihydro-6-methyl-4-(2-nitrophenyl)pyrimidine-2(1*H*)-thione (**NHCXP5**) exhibited the most potent COX-II inhibitory activity with 50.99% inhibition at 50 μg/mL (Figure 31).

To find out the better COX-II inhibitors, 5-(4-chlorophenyl)-6-(4-(methylsulfonyl)phenyl)-3-(methylthio)-1,2,4-triazine was modified by adding an ethyl side chain. These compounds also inhibit COX-I expression via interacting with Arg120. Hence, Dadashpour et al.^176^ synthesized a series of ethyl 5,6-diaryl-1,2,4-triazine-3-yl-thioacetate derivatives to inhibit cyclooxygenase (COX-II). With the help of molecular docking studies, the synthesized compound had both the COX-I/COX-II active sites with different inhibitor values (*IC*_*50*_ = 10.1 μM (COX-II), *IC*_*50*_ = 88.8 μM (COX-I)). Among all, ethyl 2-(5-(4-methoxyphenyl)-6-(4-(methylsulfonyl)-phenyl)-1,2,4-triazin-3-ylthio)acetate (**NHCXP6**) was the most selective COX-II inhibitor, which inhibited 94% of the β-amyloid fibril formation after 48 h. At last, it was found that *in silico* assessment explained that the synthesized compounds were BBB permeable and CNS active agents. The results have shown that the synthesized compound **NHCXP6** is considered a potential agent in amyloid- related diseases like AD (Figure 31).

Further, based on previously designed compounds, i.e., pyrimidine and 7-nitrobenzofurazan fluorophore, a new class of novel fluorescent COX-II inhibitors compounds have been designed and synthesized by Tietz et al.^177^ All the compounds were found to be selective COX-II inhibitors. After that, all the compounds were evaluated *in vitro* for better inhibition and selectivity value. Among all, *N*-(2-((7-nitrobenzo[*c*][1,2,5]oxadiazol-4-yl)amino)propyl)-4-[4-(methylsulfonyl)phenyl]-6-(trifluoro-methyl)-pyrimidin-2-amine (**NHCXP7**) exhibited the most inhibition of COX-II with an *IC*_*50*_ value of 1.8 μM. Then the same compound was evaluated for fluorescent COX-II visualization in human colon cancer cells. It was found that **NHCXP7** was capable of labeling the COX-II enzyme in human colon cancer cells. In the future, the attachment of NBD fluorophore at the methyl sulfone/sulfonamide moiety of an “intact” pyricoxib scaffold further improved the activity and sensitivity profile (Figure 31).

A series of novel pyrido[1,2-*a*]pyrimidin-4-one derivatives were synthesized, characterized, and evaluated *in vivo* using the carrageenan-induced rat paw edema model by Jadhav et al.^178^ with Celecoxib as a control. Some of the synthesized compounds exhibited potent COX-II inhibitory activity with *IC*_*50*_ value in the range 0.4–0.67 μM. The results revealed that 2-{2-[3-(2,4-dimethyl-phenyl)-1-*p*-tolyl-1*H*-pyrazol-4-yl]-vinyl}-3-phenyl-pyrido[1,2-*a*]pyrimidin-4-one (**NHCXP8**) exhibited maximum % inhibition of edema (72% inhibition after 3 h) and ulcer index 0.38% as compared to Celecoxib. Among all, **NHCXP8** gained more attention for the future as an anti-inflammatory drug (Figure 31). However, there are several methods reported for the pharmacological evaluation of the antiulcer drugs that beside measuring the anti-inflammatory potential also take into account the ulcerogenic potential.^179^ During the *in vivo* based assays of NSAIDs, the stomach of animal is usually dissected and the gastric ulcer or lesions formed is observed by placing it on the plane board.^180^ Though there are many methods reported, ulcer index (*U.I.*) is one of the most used methods. In this method, the *U.I.* is calculated as relative area, which is defined as the ratio of the total surface area of the stomach and lesions formed due to gastric ulcer. The *U.I.* is measured as relative area/mm^2^, where relative area of 0 corresponds to the *U.I.* of 0, relative area of 91–100 means *U.I.* of 0.1, 81–90 signifies *U.I.* of 0.2, and finally 1–10 relative area is correlated with the *U.I.* of 1.0.^181^ Another relatively easier method calculates the *U.I.* as the sum of average of the number of ulcer per animal (UN), average of severity score (US), percentage of an animal with ulcer (UP) divided by 10:^182^
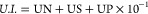


Here, the *U.I.* of 0.0 indicates normal stomach; 0.5 means redness due to inflammation of mucosa; 1.0 value confirms spot ulcers; score of 1.5 indicates hemorrhagic streaks; and *U.I.* of 2 or beyond indicates ulcers with high *U.I.* indicating high severity.

**Figure 32:**
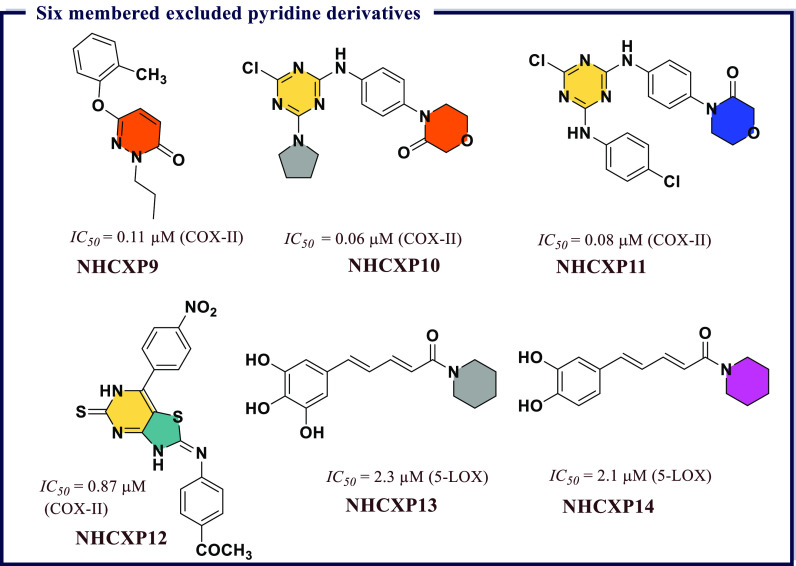
Six membered excluded pyridine derivatives as potential COX-II inhibitors.

Ibrahim et al.^183^ synthesized a novel series of 2,6-disubstituted pyridazine-3(2*H*)-one derivative and evaluated them for *in vitro* cyclooxygenase-II (COX-II) inhibitory activity. Most of the compounds exhibited activity against COX-II isozyme with an *IC*_*50*_ value in the range of 0.11–1.12 μM. Among all, 2-propyl-6-(*o*-tolyloxy) pyridazin-3(2*H*)-one (**NHCXP9**) was the most potent against COX-II with an *IC*_*50*_ value of 0.11 μM and selectivity index of 33.3. Further, **NHCXP9** was screened for *in vivo* anti-inflammatory activity using the carrageenan-induced rat paw edema method. The same compound exhibited potent anti-inflammatory activity with % inhibition of edema of 82.5% as compared to reference drug Indomethacin. With the help of structure–activity relationship (SAR), it was concluded that **NHCXP9** had good binding interaction within the active site of COX-II. These compounds exhibited better *GI* safety profile (Figure 32).

A series of compounds obtained by appending 4-aminophenylmorpholin-3-one and acyclic, cyclic, or heterocyclic moieties on 1,3,5-triazine were synthesized by Singh et al.^184^ Among all, 4 aminophenylmorpholin-3-one derivatives, i.e., **NHCXP10** and **NHCXP11**, were optimized for the best inhibition of COX-II with *IC*_*50*_ values of 0.06 and 0.08 μM, respectively, and selectivity over COX-I of 166 and >125, respectively. The *ED*_*50*_ values of **NHCXP10** and **NHCXP11** were found to be 2.2 and 1.9 mg kg^–1^, respectively, better than Indomethacin and Celecoxib. Out of these two compounds, **NHCXP10** did not exhibit toxicity even at a dose of 2000 mg kg^–1^. With the help of molecular docking studies, the better interaction of these compounds with R120, Y355 and W385 was observed. The process of electron transfer during the metabolic phase of the enzyme was due to the residues holding the substrate. As a result of this study, these compounds have interesting phenomena for the inhibition of COX enzyme (Figure 32).

Currently, a class of novel thiazolo[4,5-*d*]pyrimidines derivatives were synthesized and reported as an anti-inflammatory activity agent by Bakrset et al.^185^ Most of the synthesized compounds had moderate to high potent inhibitory action toward COX-II with a range of excellent *IC*_*50*_ values (*IC*_*50*_ = 0.87–3.78 μM). Among all, 1-(4-[7-(4-nitrophenyl)-5-thioxo-5,6-dihydro-3*H*-thiazolo[4,5-*d*]pyrimidin-2-ylideneamino]phenyl)ethanone (**NHCXP12**) with 57%, 88%, and 88% inhibition of inflammation after 1, 3, and 5 h was the most active derivative. After that, it was found that the **NHCXP12** also had the highest anti-inflammatory activity compared to Celecoxib with 43%, 43%, and 54% inhibition after 1, 3, and 5 h, sequentially. **NHCXP12** with an *IC*_*50*_ value of 0.87 μM exhibited higher selective COX-II inhibitory effect and *in vivo* ulceration effect with good ulcer index (*U.I.* = 12.25) compared to Celecoxib (*IC*_*50*_ = 1.11 μM). With the help of molecular docking studies, the excellent binding mode of most potent COX-II inhibitors (**NHCXP12**) was performed. Hence, the good anti-inflammatory activity with low ulcerogenic side effect was shown via the mixing of the thiazole scaffold with pyrimidine moiety in one hybrid structure (Figure 32).

A key enzyme involved in the biosynthesis of pro-inflammatory leukotrienes, leading to asthma, was 5-lipoxygenase (5-LOX). The highly active compounds with active functional group like multiple hydroxy and multiple methoxy groups against 5-LOX based on natural product coumarin were synthesized by Muthuaman et al.^186^ A catechol type dihydroxyl derivative (CP-209) and a vicinal trihydroxyl derivative (CP-262-F2) exhibited 82.7% and 82.5% inhibition against 5-LOX, respectively, at 20 μM. They also displayed *IC*_*50*_ values (2.1 ± 0.2 μM and 2.3 ± 0.2 μM), respectively, comparable to Zileuton (*IC*_*50*_ = 1.4 ± 0.2 μM). *In silico* ADME/TOX analysis revealed that the synthesized compounds (CP-155, 194, 209, and 262-F2) had good inhibition value and less toxic effect as compared to the existing drug. As a result of this, the most potent compounds CP-209 (**NHCXP13**) and CP-262-F2 (**NHCXP14**) exhibited good *IC*_*50*_ values of 2.1 and 2.3 μM, respectively, as compared to prominent 5-LOX inhibitor Zileuton (*IC*_*50*_ = 1.4 μM) (Figure 32).

**Figure 33:**
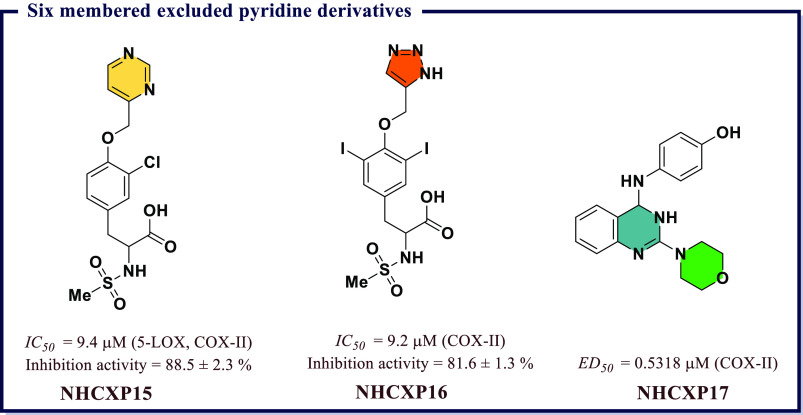
Six membered excluded pyridine derivatives as potential COX-II inhibitors.

Puratchikody et al.^187^ synthesized a series of novel tyrosine derivatives to favor the inhibition of 5-LOX and PGE_2_ production. Most of the synthesized derivatives could be further optimized and developed as drugs against inflammation and cancer. Among all the derivatives, 3-{3,5-di-iodo-4-[(1*H*-1,2,3-triazol-5-yl)methoxy]phenyl}-2-methanesulfonamidopropanoic acid (**NHCXP15**) had better inhibitory activity with percentage inhibition value (88.5 ± 2.3%) and *IC*_*50*_ value of 9.4 μM against 5-LOX comparable to that of the standard drug Zileuton at 50 μM (95.6 ± 0.7%, *IC*_*50*_ of 1–2 μM). **NHCXP15** also exhibited good inhibitory activity against the enzymes in the PG pathway (namely COX-II and mPGES_1_) inhibiting the production of PGE_2_. Another compound, 3-{3-chloro-4-[(pyrimidin-4-yl)methoxy]phenyl}-2-methane sulfonamidopropanoic acid (**NHCXP16**), also exhibited the highest inhibition (81.6 ± 1.3%, *IC*_*50*_ of 9.2 μM) of COX-II and mPGES_1_ compared to Licofelone. Hence, chlorine substituted **NHCXP16** received more attention as compared to other halogens in the inhibition of PGE_2_ production. It was found that these tyrosine derivatives could prove to be a promising scaffold against asthma, allergies, cancer, and other inflammatory diseases (Figure 33).

For the analgesic and anti-inflammatory activity, Dravyakar et al.^188^ synthesized a novel series of 2-(morpholin-4-yl)-*N*-phenylquinazolin-4-amine derivatives. All molecules were tested *in vitro* for selective analgesic and anti-inflammatory activity against COX-II using various pain models in rodents. Among all, 4-{[2-(morpholin-4-yl)-3,4-dihydroquinazolin-4-yl]amino}phenol (**NHCXP17**) was found to be significantly potent with good anti-inflammatory and analgesic activity compared to Indomethacin. With the help of molecular docking study, it was revealed that **NHCXP17** had better interaction within the active site of COX-II enzyme with a score of −1.6743. Along with 3D-QSAR study, the activity profile of **NHCXP17** suggested that it may have potential for further evaluation and development as a lead molecule for therapy in pain management (Figure 33).

### 4.15 Miscellaneous Five Membered

**Figure 34:**
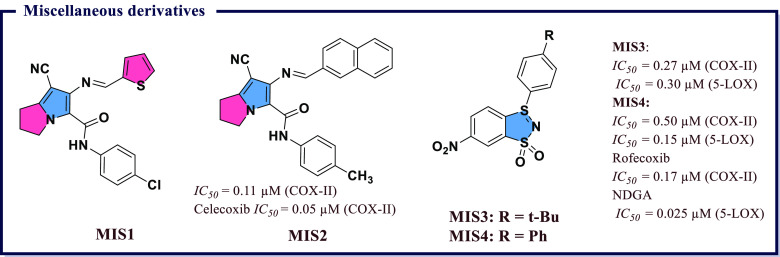
Miscellaneous derivatives as potential COX-II inhibitors.

Gouda et al.^189^ reported a set of novel pyrrolizine-5-carboxamides. The synthesized compounds were evaluated for anticancer activity against MCF-7, A549, and Hep3B cancer cell lines. These compounds also exhibited COX-I and COX-II inhibitory activity with *IC*_*50*_ values in the range 5.78–11.96 μM and 0.1–0.78 μM, respectively. Among all, (*E*)-7-cyano-6-((naphthalen-2-ylmethylene)amino)-*N*-(*p*-tolyl)-2,3-dihydro-1*H*-pyrrolizine-5-carboxamide (**MIS2**) had the most potent activity against A549 and Hep3B cell lines, and (*E*)*-N*-(4-chlorophenyl)-7-cyano-6-((thiophen-2-ylmethylene)amino)-2,3-dihydro-1*H*-pyrrolizine-5-carboxamide (**MIS1**) exhibited activity against MCF-7 cell line. **MIS1** and **MIS2** exhibited high selectivity for COX-II over COX-I. **MIS2** showed high COX-II inhibitory activity with *S.I.* greater than 100 and had the ability to induce apoptosis. The result concluded that these compounds can be good candidates for anticancer agents (Figure 34).

A series of novel benzo[1.3.2]dithiazolium ylide 1,1-dioxide derivatives were synthesized by Tan et al.^190^ and evaluated for anti-inhibitory action for COX-II and 5-LOX. Most of the compounds exhibited good safety profile against COX-II. SAR revealed that the 6-nitro group played a very vital role in anti-inhibitory action. Among all, 3-(4-(*tert*-butyl)phenyl)-6-nitro-2,3-dihydro-3-Δ^4^-benzo[*d*][1,3,2]dithiazole 1,1-dioxide (**MIS3**) and 3-([1,1′-biphenyl]-4-yl)-6-nitro-2,3-dihydro-3-Δ^4^-benzo[*d*][1,3,2]dithiazole 1,1-dioxide (**MIS4**) exhibited strong inhibitory activity against COX-II and 5-LOX. **MIS3** exhibited an *IC*_*50*_ value of 0.27 and 0.30 μM against COX-II and 5-LOX, respectively, and **MIS4** had an *IC*_*50*_ value of 0.50 and 0.15 μM against COX-II and 5-LOX, respectively (Figure 34).

### 4.16 Molecular Hybrids

**Figure 35:**
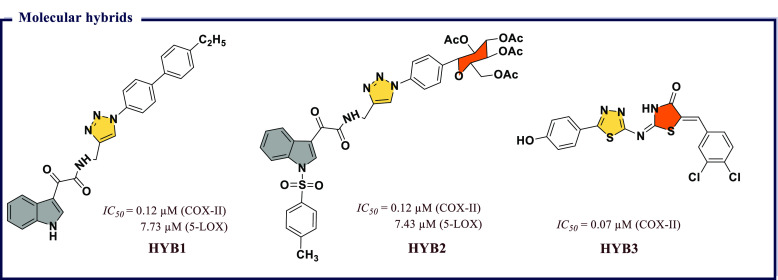
Molecular hybrids as potential COX-II inhibitors.

Naaz et al.^191^ synthesized a series of 1,2,3-tethered indole-3-glyoxamide derivatives and evaluated them for *in vivo* anti-inflammatory and *in vitro* antiproliferative activity. Among all, *N*-((1-(4′-ethyl-[1,1′-biphenyl]-4-yl)-1*H*-1,2,3-triazol-4-yl)methyl)-2-(1*H*-indol-3-yl)-2-oxoacetamide (**HYB1**) and (2*S*,4*S*,5*R*)-2-(acetoxymethyl)-6-(4-(4-((2-oxo-2-(1-tosyl-1*H*-indol-3-yl)acetamido)methyl)-1*H*-1,2,3-triazol-1-yl)phenyl)tetrahydro-2*H*-pyran-3,4,5-triyl triacetate (**HYB2**) exhibited good COX-II inhibition (*IC*_*50*_ = 0.12 μM) with excellent selectivity index over COX-I (0.058 and 0.046). **HYB1** and **HYB2** also displayed 5-LOX inhibitory activity (*IC*_*50*_ = 7.73 and 7.43 μM) compared to reference norhihydroguaiaretic acid (*IC*_*50*_ = 7.31 μM). **HYB1** also displayed excellent antiproliferative activity against DU145 prostate cancer lines. *In vitro* tubulin assay revealed that compound **HYB1** obstructed microtubulin dynamic and thus act as tubulin polymerization inhibitors. Molecular docking studies displayed good binding affinity of **HYB1** toward COX-II and 5-LOX. Both compounds inhibited the cholchicines binding site of tubulin polymer (Figure 35). A series of 4-thiazolidinone and 1,3,4-thiadiazole based molecular hybrids were developed by Omar et al.^192^ and evaluated for *in vitro* 5-LOX, COX-I, and COX-II inhibition. Most of the compounds exhibited great potency (*IC*_*50*_ = 70–100 nM) and selectivity index ranging between 220 and 55, and were also checked for *in vivo* anti-inflammatory activity. Among all, (*E*)-5-((*Z*)-3,4-dichlorobenzylidene)-2-((5-(4-hydroxyphenyl)-1,3,4-thiadiazol-2-yl)imino)thiazolidin-4-one (**HYB3**) showed excellent COX-II inhibition activity at a nanomolar concentration (*IC*_*50*_ = 70 nM and *S.I.* = 220) compared to reference drug Celecoxib (*IC*_*50*_ = 49 nM, *S.I.* = 308) and exhibited 5-LOX (*IC*_*50*_ = 11 μM) inhibiting activity compared to Zileuton (*IC*_*50*_ = 15 μM). *In silico* molecular docking studies revealed excellent binding affinity of **HYB3** on 5-LOX and COX-II (Figure 35).

**Figure 36:**
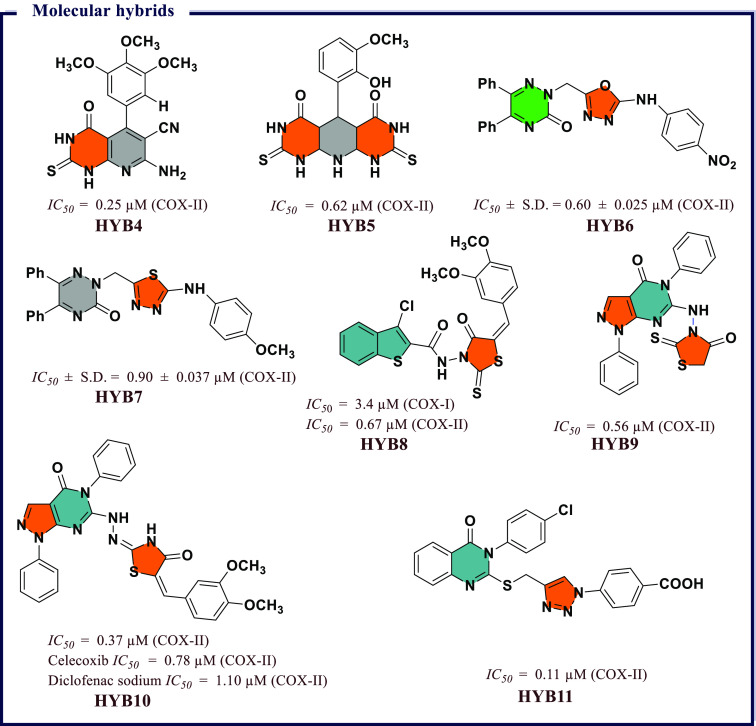
Molecular hybrids as potential COX-II inhibitors.

Abdelgawad et al.^193^ designed a sequence of novel pyrimidine-pyridine hybrids from 6-amino-2-thioxo-2,3-dihydro-1*H-*pyrimidin-4-one. Further, these novel compounds were screened for anti-inflammatory activity and ulcerogenic liability. Among all, 7-amino-5-(3,4,5-trimethoxyphenyl)-4-oxo-2-thioxo-1,2,3,4-terahydropyrido[2,3-*d*]pyrimidine-6-carbonitrile (**HYB4**) and 9-(2-hydroxy-3-methoxyphenyl)-1,3,6,8,9,10-hexahydro-2,7-dithioxopyrido[2,3-d:6,5d′]dipyrimidine-4,5-dione (**HYB5**) were found to be selective inhibitors of COX-II over COX-I compared to Celecoxib (*IC*_*50*_ = 1.11 μM). Further, ulcerogenic liability studies revealed that **HYB4** and **HYB5** were less ulcerogenic than Celecoxib and Indomethacin. *In silico* molecular docking studies showed that these compounds showed similar interactions and binding patterns as observed for cocrystallized ligand SC-558 (Figure 36).

Banerjee et al.^194^ synthesized a series of novel 5,6-diphenyl-1,2,4-triazin-3(2*H*)-ones containing 1,2,4-triazole and 1,3,4-oxadiazole/thiadiazole. Among all, 2-((5-((4-nitrophenyl)amino)-1,3,4-oxadiazol-2-yl)methyl)-5,6-diphenyl-1,2,4-triazin-3(2*H*)-one (**HYB6**) and 2-((5-((4-methoxyphenyl)amino)-1,3,4-thiadiazol-2-yl)methyl)-5,6-diphenyl-1,2,4-triazin-3(2*H*)-one (**HYB7**) did not induce any gastric or renal toxicity in rats. Compounds **HYB6** and **HYB7** reduced MDA content on gastric mucosa and also were found to be good COX-II inhibitors (*IC*_*50*_ = 0.60 and 0.90 μM) and selective (*S.I.* = 104.07 and 66.73) over COX-I as well. Molecular docking and dynamics studies also showed a well-defined binding interaction of **HYB6** and **HYB7** within the active site of COX-II (Figure 36).

Miligy et al.^195^ synthesized new hybrid molecules containing benzothiophene/benzofuran with rhodamine and screened for *in vitro* COX/LOX inhibitory activity. Among all, (*E*)-3-chloro-*N*-(5-(3,4-dimethoxybenzylidene)-4-oxo-2-thioxothiazolidin-3-yl)benzo[*b*]thiophene-2-carboxamide (**HYB8**) had COX-II inhibitory activity higher than reference drug Celecoxib and also exhibited excellent selectivity index (*S.I.* = 5.1) near to that of Celecoxib (*S.I.* = 6.7). Interestingly, this compound exhibited LOX inhibition also twice than that of meclofenamate sodium. Further, **HYB8** was tested for *in vivo* anti-inflammatory activity by using formalin-induced paw edema and gastric ulcerogenic activity tests. It was found that **HYB8** decreased formalin-induced paw edema volume. *In silico* molecular docking studies revealed the presence of good binding interaction within the active site of COX-II and 5-LOX, suggesting this series to be a good candidate for anti-inflammatory activities (Figure 36).

Tageldin et al.^196^ developed two novel sequence of [3,4-*d*]pyrimidine containing thiazolidinone moieties and screened them for *in vitro* COX-I and COX-II inhibitory activities. Most of the compounds exhibited COX-II selectivity and inhibitory activity. The compounds with promising inhibition were evaluated *in vivo* for anti-inflammatory activity using formalin-induced paw edema method and cotton pellet induced granuloma by considering Celecoxib and Diclofenac sodium as standard drugs. Among all, 3-((4-oxo-1,5-diphenyl-4,5-dihydro-1*H*-pyrazolo[3,4-*d*]pyrimidin-6-yl)amino)-2-thioxothiazolidin-4-one (**HYB9**) and (*E*)-5-((*E*)-3,4-dimethoxybenzylidene)-2-(2-(4-oxo-1,5-diphenyl-4,5-dihydro-1*H*-pyrazolo[3,4-*d*]pyrimidin-6-yl)hydrazono)thiazolidin-4-one (**HYB10**) displayed good anti-inflammatory activity higher than that of Celecoxib and Diclofenac sodium. Moreover, **HYB9** and **HYB10** exhibited a good gastrointestinal safety profile. Interestingly, these compounds can be promising candidates in managing acute and chronic inflammation (Figure 36).

Moussa et al.^197^ manufactured a new sequence of thioquinazolinone derivatives that consist of propargyl moiety, 1,2,3-triazolyl, and isoxazolyl rings using click chemistry. These compounds were further screened for *in vitro* inhibitory activity against 5-LOX and COX-I/II. The results showed that 4-(4-(((3-(4-chlorophenyl)-4-oxo-3,4-dihydroquinazolin-2-yl)thio)methyl)-1*H*-1,2,3-triazol-1-yl)benzoic acid (**HYB11**) exhibited COX-II inhibitory activity with an *IC*_*50*_ value of 0.11 μM, respectively, as compared to reference drugs Celecoxib, Diclofenac, and INM (*IC*_*50*_ value 0.05, 0.8, and 0.49 μM respectively). In addition to this, **HYB11** exhibited 5-LOX inhibitory activity with an *IC*_*50*_ value of 7.62 μM in comparison to Zileuton (*IC*_*50*_ = 2.41 μM) and Meclofenamate sodium (*IC*_*50*_ = 5.64 μM). **HYB11** displayed *in vivo* anti-inflammatory activity using formalin-induced rat paw edema test (Figure 36).

**Figure 37:**
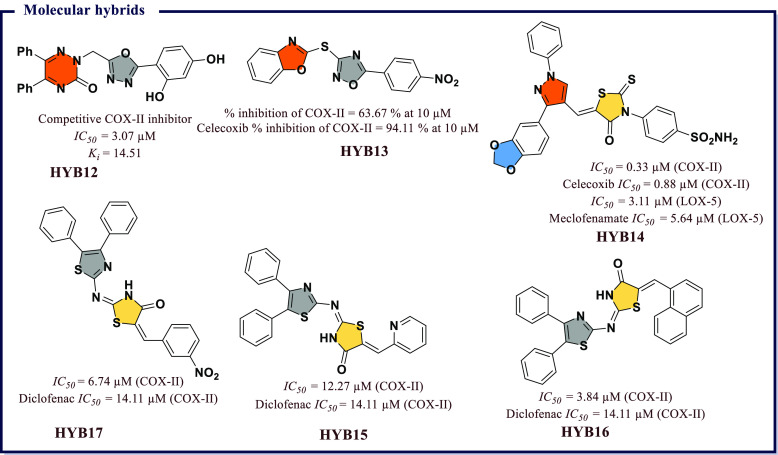
Molecular hybrids as potential COX-II inhibitors.

Banerjee et al.^198^ reported a sequence of triazin-3(2*H*)-one derivatives containing 1,3,4-oxadiazole and evaluated them for *in vitro* anti-inflammatory and antianalgesic activities using an albumin denaturation assay. Most of the compounds exhibited significant anti-inflammatory activity with lesser ulcerogenic liabilities as compared to standard drug INM. Among all, 2-((5-(2,4-dihydroxyphenyl)-1,3,4-oxadiazol-2-yl)methyl)-5,6-diphenyl-1,2,4-triazin-3(2*H*)-one (**HYB12**) was found to be most potent against COX-II enzyme with an *IC*_*50*_ value of 3.07 μM (Figure 37).

Razik et al.^199^ synthesized two series of benzodioxole-pyrazole derivatives and evaluated them for COX-I/II and 5-LOX inhibitory activity. The synthesized compounds were screened for *in vivo* analgesic and anti-inflammatory activity using diclofenac sodium as standard drug. The results exposed that some of them had shown effective COX-II inhibitory activity in the range of 0.33–3.56 μM. Among all, (*Z*)-4-(5-((3-(benzo[*d*][1,3]dioxol-5-yl)-1-phenyl-1*H*-pyrazol-4-yl)methylene)-4-oxo-2-thioxothiazolidin-3-yl)benzenesulfonamide (**HYB14**) was most potent with an excellent *IC*_*50*_ value (0.33 μM). In addition, most of the compounds exhibited 5-LOX inhibitory activity with *IC*_*50*_ values in the range of 3.11–9.94 μM. The same compound exhibited the most potent 5-LOX inhibitory activity (*IC*_*50*_ = 3.11 μM). *In silico* molecular docking studies revealed that **HYB14** had nearly same binding patterns with COX-II and 5-LOX as that of Celecoxib and Meclofenamic acid, respectively (Figure 37).

A sequence of diphenylthiazole-thiazolidinone derivatives were developed by Abdelzeem et al.^200^ and further screened for *in vitro* COX-I/II and *in vivo* anti-inflammatory activity using diclofenac as positive control. Among all, 2-((4,5-diphenylthiazol-2-yl)imino)-5-(pyridin-3-ylmethylene)thiazolidin-4-one (**HYB15**), 2-((4,5-diphenylthiazol-2-yl)imino)-5-(naphthalen-1-ylmethylene)thiazolidin-4-one (**HYB16**), and 2-((4,5-diphenylthiazol-2-yl)imino)-5-(3-nitrobenzylidene)-thiazolidin-4-one (**HYB17**) exhibited potent inhibitory activity against COX-II with good *IC*_*50*_ values in the range of 2.03–12.27 μM with different selectivity index. Then docking studies revealed good binding affinity for **HYB15**, **HYB16**, and **HYB17** in the active site of both COX enzymes (Figure 37).

**Figure 38:**
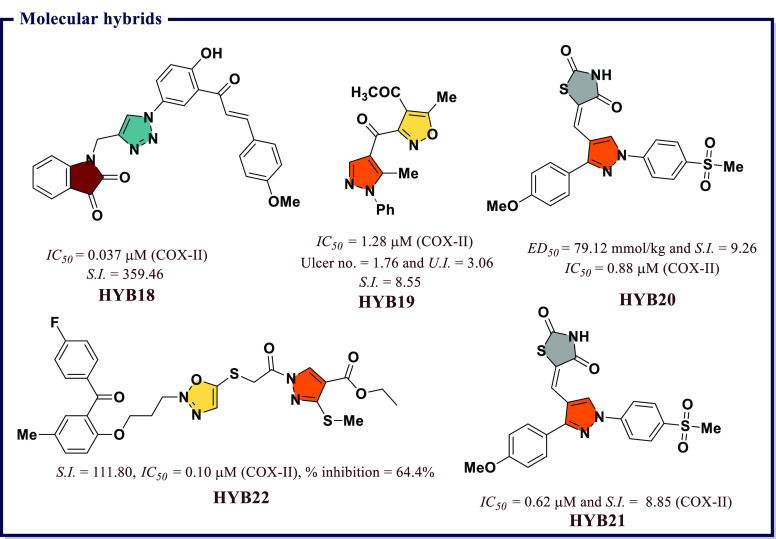
Molecular hybrids as potential COX-II inhibitors.

Boshra et al.^201^ synthesized chalcone phenyltriazole derivatives using click reaction between azido chalcone derivatives and phenyl acetylene. The synthesized compounds were evaluated *in vitro* and *in vivo* for anti-inflammatory activities. Most of the compounds were selective and dual inhibitors of COX-II (*IC*_*50*_ = 0.037–0.041 μM) and 5-LOX (*IC*_*50*_ = 1.41–1.80 μM) with excellent selective index (*S.I.*) in the range of 32.17–360.53 compared to Celecoxib and Indomethacin (INM). Hence, it was seen that isatin series displayed higher potency than Zileuton. Among all, (*E*)-1-((1-(4-hydroxy-3-(3-(4-methoxyphenyl)acryloyl)phenyl)-1*H*-1,2,3-triazol-4-yl)methyl)indoline-2,3-dione (**HYB18**) displayed the highest inhibitory activity against COX-II with an *IC*_*50*_ values of 0.037 μM and good selective index (*S.I.* = 359.46). Docking studies revealed selectively and good binding affinity of **HYB18** with COX-II and 5-LOX. **HYB18** fit within the active site of COX-II and 5-LOX better than the chloro-substituted one, which failed to interact with Arg513 residue in the side pocket (Figure 38).

Abdelall et al.^202^ synthesized isoxazole derivatives and evaluated them *in vivo* for anti-inflammatory activity. Many derivatives showed nearly equal inhibitory potency to that of Celecoxib. Along with this, the molecules exhibited better selectivity than Celecoxib. Among all, 1-(5-methyl-3-(5-methyl-1-phenyl-1*H*-pyrazole-4-carbonyl)isoxazol-4-yl)ethan-1-one (**HYB19**) had (*U.I.* = 3.06, *IC*_*50*_ = 1.28) excellent activity compared to Celecoxib (*IC*_*50*_ = 6.70 μM). **HYB19** exhibited greater safety profile and selectivity (*S.I.* = 8.55) compared to Indomethacin and Celecoxib, respectively. Molecular docking studies revealed good binding affinity and selectivity of **HYB19** over than Celecoxib (Figure 38).

Abdellatif et al.^203^ synthesized two series containing pyrazole ring with vicinal diaryl rings as selective COX-II moiety and thiazolidindione or thiazolidinone derivatives and evaluated them for their COX inhibition. The synthesized compounds possessed weak inhibitory activities against COX-I (*IC*_*50*_ = 3.55–10.87 μM range) but showed high COX-II inhibitory activities (*IC*_*50*_ = 0.48–1.92 μM range) with good selective index in the range of 5.66–9.26 compared to reference drug Celecoxib (COX-I *IC*_*50*_ = 7.23 μM, COX-II *IC*_*50*_ = 0.84 μM, and *S.I.* = 8.60). Among all, (*E*)-5-((3-(4-methoxyphenyl)-1-(4-(methylsulfonyl)phenyl)-1*H*-pyrazol-4-yl)methylene)thiazolidine-2,4-dione (**HYB20**) and (*E*)-5-((3-(4-methoxyphenyl)-1-(4-(methylsulfonyl)phenyl)-1*H*-pyrazol-4-yl)methylene)thiazolidine-2,4-dione (**HYB21**) exhibited excellent inhibitory activity against COX-II. Both compounds have good selective index values (*S.I.* = 9.26 and 8.85, respectively) and low ulcerogenic effects with significant ulcer index (3.97 and 3.12, respectively). **HYB20** was slightly more potent with good *ED_50_* value (79.12 μmol/kg)) than Celecoxib (*ED50* = 82.2 μmol/kg). Along with this, **HYB21** displayed good selective index against COX-II with excellent *IC*_*50*_ (*IC*_*50*_ = 0.62 μM and *S.I.* = 8.85). Docking studies revealed that **HYB20** and **HYB21** exhibited good binding mode and selectivity in the most active site of COX-II (Figure 38).

A series of novel benzophenones conjugated with oxadiazole sulfur bridge pyrazole moiety were synthesized by Zabiullaa et al.^204^ and evaluated for anti-inflammatory and analgesic properties. The activity data revealed that most of the compounds with electron withdrawing halo groups showed potent anti-inflammatory activity. Among all, **HYB22** exhibited effective COX-II inhibitory activity (*IC*_*50*_ = 0.10 μM) compared to standard drug. *In silico* docking studies revealed that the binding energy of **HYB22** was found in the range of −4.62 to −6.62 kcal/mol, respectively (Figure 38).

**Figure 39:**
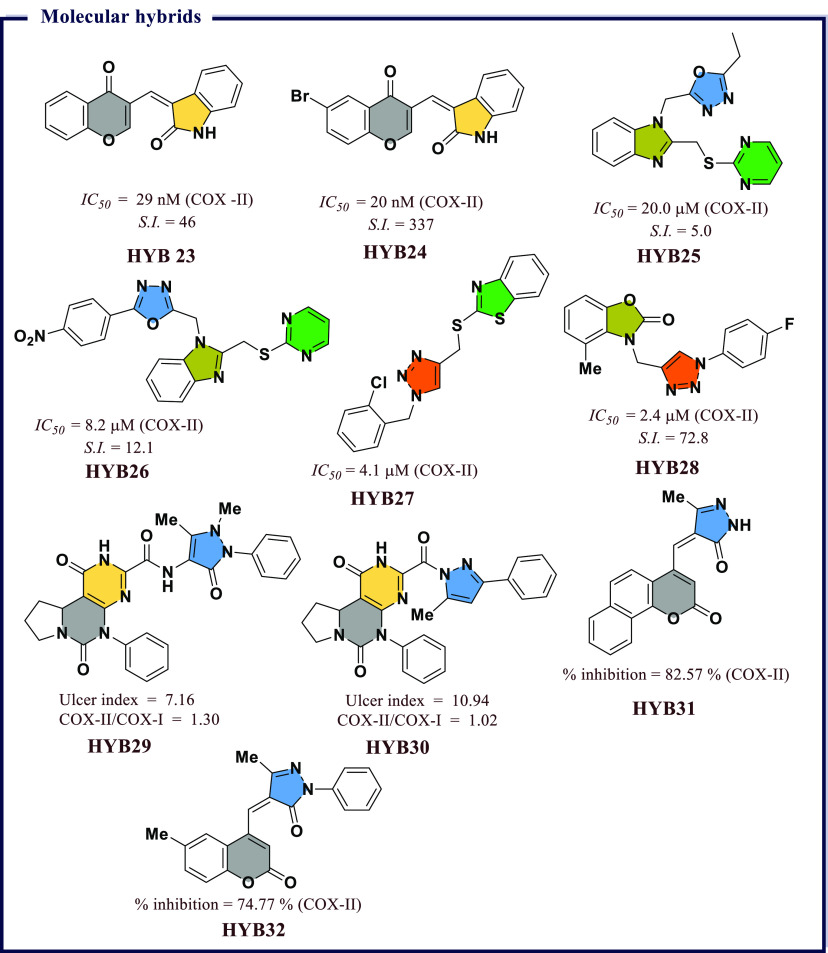
Molecular hybrids as potential COX-II inhibitors.

A sequence of chromone-indole and chromone-pyrazole derivatives were synthesized by Shaveta et al.^205^ In comparison to chromone and indole based drugs, combination of chromone and oxindole derivatives resulted in considerable inhibition and selectivity for COX-II over COX-I. Among all, 3-(4-oxo-4*H*-chromen-3-yl-methylene)-1,3-dihydroindol-2-one (**HYB23**) and 3-(6-bromo-4-oxo-4*H*-chromen-3-yl-methylene)-1,3-dihydroindol-2-one (**HYB24**) were identified as preferred inhibitors of COX-II over COX-I and 5-LOX. **HYB23** and **HYB24** exhibited excellent COX-II inhibition activity with good *IC*_*50*_ values (29 nM and 20 nM, respectively) and selectivity indices (*S.I.* = 46 and 337) for COX-II over COX-I. Molecular docking studies revealed the preferential interactions of **HYB23** and **HYB24** with COX-II enzyme. **HYB23** displayed good analgesic potency as compared to diclofenac. In addition to the biological profile, the desirable physio-chemical properties of these compounds make them promising leads for anti-inflammatory drugs (Figure 39).

Rathore et al.^206^ synthesized new benzimidazoles endowed with oxadiazole and screened them for *in vitro* inhibitory activity against COXs enzyme. The synthesized compounds exhibited moderate inhibitory activity with *IC*_*50*_ values in the range of 11.6–56.1 μM. **HYB26**, 1-((5-(4-nitrophenyl)-1,3,4-oxadiazol-2-yl)methyl)-2-((pyrimidin-2-ylthio)methyl)-1*H*-benzo[*d*]imidazole, had significant COX-II inhibition with an *IC*_*50*_ value of 8.2 μM with 68.4% inhibition. Along with this, 1-((5-ethyl-1,3,4-oxadiazol-2-yl)methyl)-2-((pyrimidin-2-ylthio)methyl)-1*H*-benzo[*d*]imidazole (**HYB25**) displayed moderate cytotoxicity toward the UO-31 cell line of renal cancer. Docking studies revealed excellent binding affinity into the binding sites of the COX enzymes. Thus, **HYB26** could serve as a lead compound for developing new COX-II inhibitors (Figure 39).

A series of 2-mercapto benzothiazole linked with triazoles were synthesized by Yatam et al.^207^ Further, the synthesized compounds were evaluated as COX-II inhibitors. The molecular level interactions of the designed library indicated that the aryl ring united with triazole occupying the mefenamic acid in the COX-II active site. Among all, 2-(((1-(2-chlorobenzyl)-1*H*-1,2,3-triazol-4-yl) methyl)thio)benzo[*d*]thiazole (**HYB27**) displayed the most significant COX-II inhibitory activity with an *IC*_*50*_ of 4.1, 4.3, and 5.4 μM, respectively. The time-dependent increase in inhibition of inflammation *in vivo* anti-inflammatory evaluation was noticed. The biological potential of benzothizoles as COX-II inhibitors resulted in potent anti-inflammatory agents with admirable efficacy (Figure 39).

Haider et al.^208^ synthesized a series of novel *bis*-heterocycles containing benzoxazolinone based 1,2,3-triazoles and tested their anti-inflammatory activity using the carrageenan induced hind paw edema method. One of the lead molecules exhibited potent, selective inhibition of COX-II (59.48%), while Celecoxib achieved 66.36% inhibition. The molecular docking studies revealed that some compounds exhibited strong inhibitory effect due to the extra stability of the complexes driven by additional ligand COX-II interactions. The histopathology report showed that none of the compounds caused gastric ulceration. Among all, 3-((1-(4-fluorophenyl)-1*H*-1,2,3-triazol-4-yl)methyl)-4-methylbenzo[*d*]oxazol-2(3*H*)-one (**HYB28**, in Figure 38) (COX-I *IC*_*50*_ = 174.72 μM; COX-II *IC*_*50*_ = 2.4 μM; *S.I.* = 72.8) exhibited potent selective COX-II inhibition compared to Celecoxib (COX-I *IC*_*50*_ = 25.74 μM; COX-II *IC*_*50*_ = 0.32 μM; *S.I.* = 80.43).

A new series of pyrimido[5,4-*e*]pyrrolo[1,2-*c*]pyrimidines were reported by Hanna et al.^209^ Most of the synthesized compounds exhibited less ulcerogenic effect than the reference drugs Indomethacin and Celecoxib. For example, *N*-(1,5-dimethyl-3-oxo-2-phenyl-2,3-dihydro-1*H*-pyrazol-4-yl)-1,6-dioxo-5-phenyl-1,2,5,6,8,9,10,10*a*-octahydropyrimido[5,4-*e*]pyrrolo[1,2-*c*]pyrimidine-3-carboxamide (**HYB29**, in Figure 38) showed an *IC*_*50*_ of 6.00 mmol/kg and low ulcerogenic index. **HYB29** and 3-(5-methyl-3-phenyl-1*H*-pyrazole-1-carbonyl)-5-phenyl-8,9,10,10*a*,-tetrahydropyrimido[5,4-*e*]pyrrolo[1,2-*c*]pyrimidine-1,6(2*H*,5*H*)-dione (**HYB30**) showed almost equal inhibitory effect on both isoenzymes.

Kulkarni et al.^210^ reported a sequence of novel coumarin-pyrazolone hybrids. These synthesized compounds were further evaluated *in vitro* for anti-inflammatory and anticancer activity. Most of them exhibited potent anti-inflammatory activity against COX-II enzyme. (*Z*)-5-Methyl-4-((2-oxo-2*H*-benzo[*h*]chromen-4-yl)methylene)-2,4-dihydro-3*H*-pyrazol-3-one (**HYB31**) and (*Z*)-5-methyl-4-((6-methyl-2-oxo-2*H*-chromen-4-yl)methylene)-2-phenyl-2,4-dihydro-3*H*-pyrazol-3-one (**HYB32**) exhibited potent activity with good % of inhibition of egg albumin at 100 μg/mL (82.52 and 74.77%, respectively) (Figure 39).

**Figure 40:**
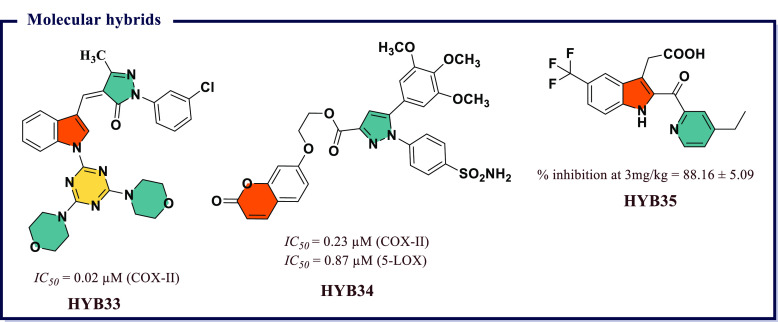
Molecular hybrids as potential COX-II inhibitors.

A library of hybrid molecules formed by the combination of triazin-indole adducts with morpholine/piperidine/pyrrolidine and pyrazole/oxindole/pyrimidine moieties was reported by Kaur et al.^211^ Further, the synthesized compounds were screened for *in vitro* COX inhibitory activity. All the compounds were tested in triplicate at different concentrations, and an *IC*_*50*_ value of each compound was calculated. Among all, (*Z*)-2-(3-chlorophenyl)-4-((1-(4,6-dimorpholino-1,3,5-triazin-2-yl)-1*H*-indol-3-yl)methylene)-5-methyl-2,4-dihydro-3*H*-pyrazol-3-one (**HYB33**) was found to be the most potent with an *IC*_*50*_ value of 0.02 μM. **HYB33** had good selectivity index for COX-II (*S.I.* = 150) as compared to Indomethacin and Diclofenac. *In vivo* anti-inflammatory studies were also consistent with *in vitro* studies. Mechanistic studies on Swiss albino mice revealed the selectivity for COX-II and minimum toxicity. Molecular docking studies and QSAR analyses predicted the binding modes of these compounds, which were in good agreement with the solution phase NMR experimental data (Figure 40).

Shen et al.^212^ had reported a new sequence of pyrazole and coumarin. The synthesized compounds were evaluated for COX-II and 5-LOX inhibitory activity. All of the tested compounds exhibited COX-II/5-LOX inhibitory activity. Among all, 2-((2-oxo-2*H*-chromen-7-yl)oxy)ethyl-1-(4-sulfamoylphenyl)-5-(3,4,5-trimethoxyphenyl)-1*H*-pyrazole-3-carboxylate (**HYB34**) having three methoxy groups on the phenyl ring exhibited excellent activity against COX-II. **HYB34** exhibited COX-II inhibitory activity with a good *IC*_*50*_ value (0.23 μM) compared with the reference drug Celecoxib (*IC*_*50*_ = 0.41 μM). Along with this, **HYB34** exhibited potent 5-LOX (*IC*_*50*_ = 0.87 μM) inhibitory activity as compared to Zileuton (*IC*_*50*_ = 1.35 μM). Furthermore, the compound resulted in human nonsmall cell lung cancer A549 cell apoptosis and blocked the cell cycle at G2 phase in a dose dependent manner (Figure 40).

A series of [2-{[(4-substituted)-pyridin-2-yl]carbonyl}-(6-or 5-substituted)-1*H*-indol-3-yl]acetic acids were synthesized by Hayashi et al.^213^ and evaluated for anti-inflammatory and antipyritic activities. All the synthesized compounds exhibited good COX-II inhibitory activity. Among them, {2-[(4-ethylpyridin-2-yl)carbonyl]-5-(trifluoromethyl)-1*H*-indol-3-yl}acetic acid (**HYB35**) was found to be most potent with anti-inflammatory activity. It exhibited *in vivo* antipyritic effect and anti-inflammatory activities against peripheral edema-formation model by carrageenan in the SD rats with suppression of the production of PGE_2_ in the edema site (Figure 40).

**Figure 41:**
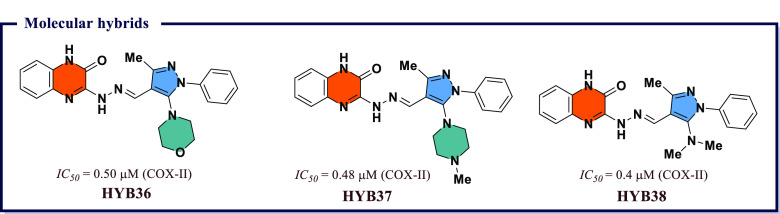
Molecular hybrids as potential COX-II inhibitors.

Galal et al.^214^ synthesized new quinoxalin-2(1*H*)-ones from 3-(2-((5-chloro-3-methyl-1-phenyl-1*H*-pyrazol-4-yl)methylene)hydrazinyl)quinoxalin-2(1*H*)-one and evaluated them against MCF-7. The selectivity of these compounds was also evaluated against human TRK compared to cisplatin. A molecular docking study was also performed to gain comprehensive understanding of the plausible binding modes and to conclude the structure activity relationships of the synthesized compounds. All the synthesized compounds exhibited *in vitro* COX-II inhibitory activity. Among them, 3-(2-((3-methyl-5-morpholino-1-phenyl-*1H*-pyrazol-4-yl)methylene)hydrazinyl)quinoxalin-2(1*H*)-one (**HYB36**), 3-(2-((3-methyl-5-(4-methylpiperazin-1-yl)-1-phenyl-1*H*-pyrazol-4-yl)methylene)hydrazinyl)quinoxalin-2(1*H*)-one (**HYB37**), and 3-(2-((5-(dimethylamino)-3-methyl-1-phenyl-1*H*-pyrazol-4-yl)methylene)hydrazinyl)quinoxalin-2(1*H*)-one (**HYB38**) exhibited excellent *IC*_*50*_ values (*IC*_*50*_ = 0.50, 0.48, and 0.4 μM, respectively) compared to Celecoxib. These compounds showed potent inhibition activity against COX-II (*IC*_*50*_: 0.40–8.50 μM) compared to COX-I (*IC*_*50*_ > 50 μM) (Figure 41).

### 4.17 Other Fused Inhibitors

**Figure 42:**
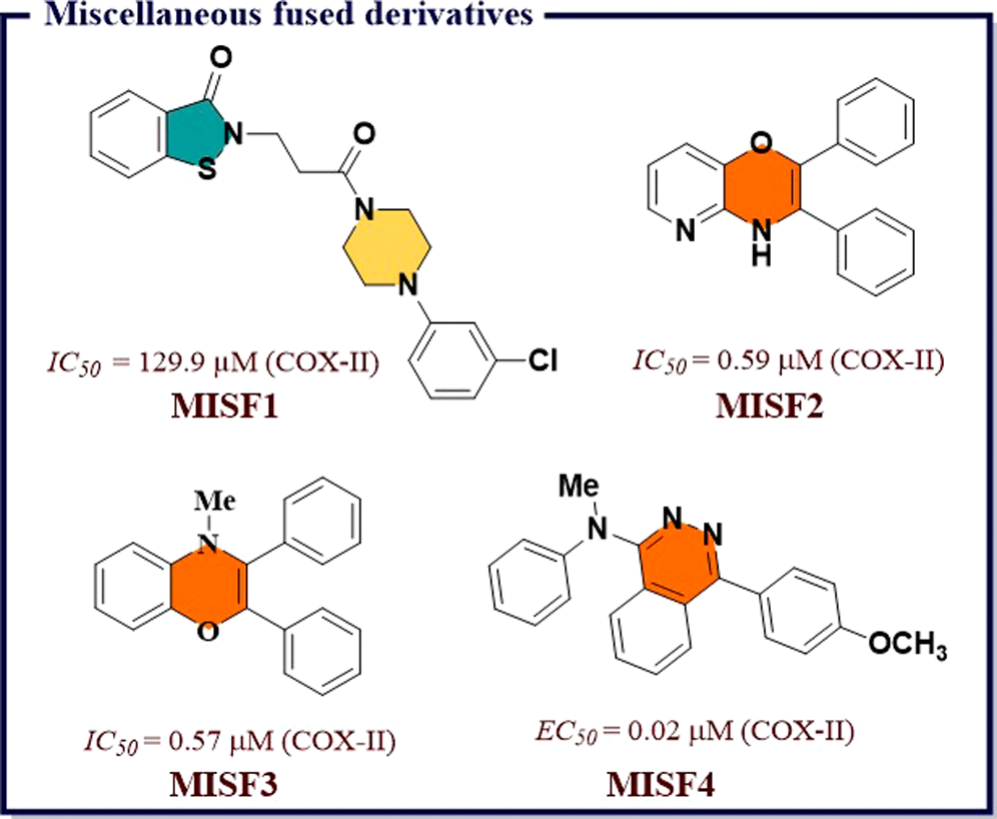
Miscellaneous fused derivatives as potential COX-II inhibitors.

Swiatek et al.^215^ reported a series of isothiazolopyridine/benzisothiazole derivatives and evaluated them for COX-I and COX-II inhibitory activities. All the synthesized compounds were found to possess good anti-inflammatory activity. Among all, 2-{3-*oxo*-3-[4-(3-chlorophenyl)piperazin-1-yl]propyl}-1,2-benzisothiazol-3(2*H*)-one (**MISF1**) exhibited the highest inhibition against COX-II with an *IC*_*50*_ value of 129.9 μM compared with piroxicam (*IC*_*50*_ = 102.8 μM). The low energy binding mode of **MISF1** was explored with the help of molecular docking studies (Figure 42).

Shaikh et al.^216^ synthesized novel 1,4-benzoxazine derivatives and screened them for anti-inflammatory activity. Optimal COX-II inhibition with *IC*_*50*_ values of 0.57–0.72 μM was observed from all the compounds with a selectivity index (*S.I.*) ranging between 186.8 and 242.4 as compared to standard drug Celecoxib (*IC*_*50*_ = 0.30 μM; COX-II *S.I.*: 4303). Excellent potency was observed in case of 2,3-diphenyl-4*H*-pyrido[3,2-*b*][1,4]oxazine (**MISF2**) and 4-methyl-2,3-diphenyl-4*H*-benzo[*b*][1,4]oxazine (**MISF3**) with an *IC*_*50*_ value of 0.59 and 0.57 μM, respectively, as compared to Celecoxib (*IC*_*50*_ = 0.30 μM). This study identified potential lead compounds for further development of novel anti-inflammatory agents (Figure 42).

Medaa et al.^217^ developed a series of aminophthalazines and evaluated their inhibitory activity against COX-II in a cell free assay. High reduction of PGE_2_ levels in between 97.2 and 98.9% with good *EC*_*50*_ between 0.038 and 0.02 μM, respectively, was observed. Among all, 4-(4-methoxyphenyl)-*N*-methyl-*N*-phenylphthalazin-1-amine (**MISF4**) exhibited most potent activity in cells (*EC*_*50*_ = 0.02 μM) with 3% inhibition of COX-II activity at 5 μM. Furthermore, antitumor activity of the synthesized analogs were analyzed in xenograft mouse models with good anticancer activity (Figure 42).

**Figure 43:**
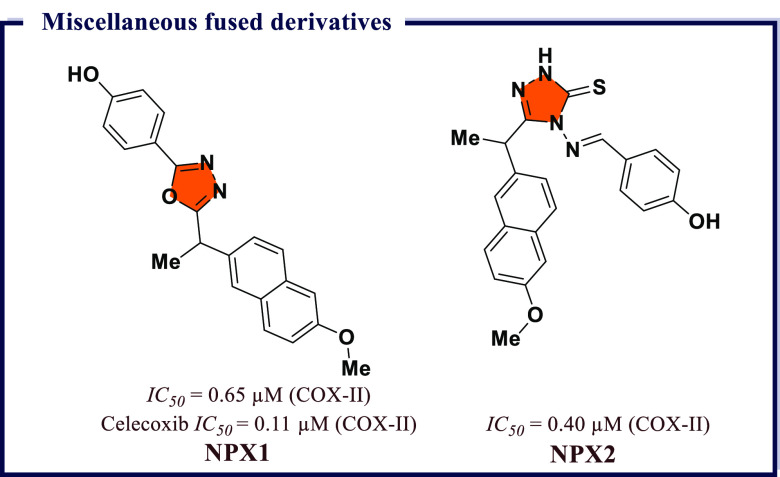
Miscellaneous fused derivatives as potential COX-II inhibitors.

Husseiny et al.^218^ synthesized a series of noncarboxylic naproxen analogues containing oxadiazoles, cycloalkanes, cyclic imides, and triazoles. The molecules were tested *in vitro* for antitumor activity using MTT assay against five cancer cell lines, i.e., MCF-7, MDA-231 HeLa, HCT-116, and Caco-2. All the compounds exhibited weak to moderate antitumor activity. Among them, 4-(5-(1-(6-methoxynaphthalen-2-yl)ethyl)-1,3,4-oxadiazol-2-yl)phenol (**NPX1**) and 4-((4-hydroxybenzylidene)amino)-3-(1-(6-methoxynaphthalen-2-yl)ethyl)-1*H*-1,2,4-triazole-5(4*H*)-thione (**NPX2**) exhibited most potent antitumor activity against all cancer cell lines compared with standard drugs Celecoxib, Afatinib, and Doxorubicin. **NPX1** and **NPX2** showed the most potent inhibitory activity against COX-II with *IC*_*50*_ values of 0.65 and 0.40 μM, respectively. These compounds were further docked within the active site of COX-II and revealed similar binding modes as observed in SC-558 (Figure 43).

The most commonly used anticancer drugs are platinum-based drugs, which are costly and toxic in nature. In view of the toxic side effects and issue of drug resistance, nonplatinum drugs, such as ruthenium-based complexes, have gained considerable interest in cancer therapy. These compounds are more effective than cisplatin and NSAIDs.

**Figure 44:**
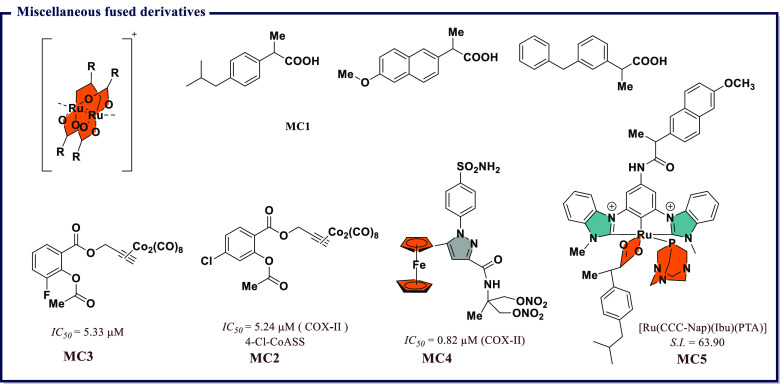
Miscellaneous fused derivatives as potential COX-II inhibitors.

Santos et al.^219^ synthesized a new diruthenium (II, III) complex [Ru_2_Cl(ket)_4_] called ruket, which contains the nonsteroidal anti-inflammatory drug ketoprofen. The synthesized compounds were characterized by electrospray ionization mass spectrometry (ESI-MS), UV–vis-IR, and Raman vibrational spectroscopies. These characterization techniques revealed the presence of a mixed-valent diruthenium (II, III) (**MC1**) multiple bonded core with four Ketoprofen ligands. Initially, a class of Ruket and its analogues with mild antiproliferative activity were tested on the colorectal cancer cells HT-29 and Caco-2. Ruket and its analogues containing ibuprofen, ruibp, runpx, and naproxen-derivatives had a significant role in COX-II inhibition. It must be noted that the diruthenium–NSAID derivatives have some effects on the production/activity of **MMP-2** and **MMP-9** in HT-29 cells due to high levels of COX-II expression. It was suggested that COX-II inhibition by these derivatives was partially involved in various pharmacological effects (Figure 44).

Literature studies revealed that organometallic derivatives had a better inhibition effect on the growth of various tumor cell lines and COX isoenzymes. With this objective, Obermoser et al.^220^ synthesized [(prop-2-ynyl)-2-acetoxybenzoate]dicobalthexacarbonyl (**Co-ASS**). The selectivity of these derivatives against COX-II was enhanced by introducing a chlorine substituent at the third, fourth, fifth, or sixth position of ASS moiety, respectively. After chlorination, it was observed that the most active compound inhibits COX-I up to 25% at 10 μM, while the inhibition rate of COX-II was 65% at 10 μM. Further, chlorination at different active position of ASS moiety had no effects on the HT-29 cell (*IC*_*50*_ = 1.5–2.7 μM), but a slight decrease was observed against MDA-MB-231 cells with Co-ASS (*IC*_*50*_ = 10.1 μM) < 3–Cl-Co-ASS (*IC*_*50*_ = 8.04 μM) ≈ 5–Cl-Co-ASS (*IC*_*50*_ = 7.85 μM) < 4–Cl-Co-ASS (*IC*_*50*_ = 5.24 μM) (**MC2**). Compared to this, the 6-chloro derivative was considerably less potent with an *IC*_*50*_ value of 22 μM on both cell lines. With the exception of 6-Cl derivatives, in cellular systems, all compounds showed notable antitumor activity in COX-I/II against tumor cells HT-29 (*IC*_*50*_ = 1.5–2.7 μM), MDA-MB-231 (*IC*_*50*_ = 5.2–8.0 μM), but less active against MCF-7 breast cancer cell line (*IC*_*50*_ = 15.2–22.9 μM). Hence, these results demonstrated that the interference with the COX-I/II cascade contributes to the anticancer effects of the cobalt alkyne complexes (Figure 44). Currently, the chemistry of cobalt alkyne complexes has gained a significant interest due to their anticancer properties and ability to target both cyclooxygenases (COX-I and COX-II). The synthesis of cobalt alkyne complex like [(prop-2-ynyl)-2-acetoxybenzoate]dicobalthexacarbonyl (Co-ASS) was achieved by Baecker et al.^221^ In MCF-7 cells, the metabolic activity was even unaffected up to a concentration of 40 μM. Among all these derivatives, 6F–Co-ASS complexes strongly reduced the PGE_2_ synthesis in HT-29 cells and inhibited COX-II more effectively than COX-I. The higher quantification of fluorine by HR CS MAS makes this method about 5-fold more sensitive than HR CS AAS measuring cobalt. The Co-ASS reduced the cell mass of HT-29 (*IC*_*50*_ = 1.15 μM) more effectively than cisplatin (*IC*_*50*_ = 3.52 μM). Against the breast cancer cell lines, Co-ASS exhibited minor activity (MDA-MB-231: *IC*_*50*_ = 10.1 μM; MCF-7: *IC*_*50*_ = 16.4 μM). Fluorine substituents at third (*IC*_*50*_ = 2.78 μM), fourth (*IC*_*50*_ = 3.33 μM), or fifth (*IC*_*50*_ = 1.73 μM) position of the ASS moiety of Co-ASS marginally influenced the effects at the HT-29 cell line, while 6F–Co-ASS (**MC3**) with an excellent *IC*_*50*_ (20.2 μM) was 10-fold less active than its isomer (Figure 44).

Furthermore, to remove the side effect of NSAIDs, classes of ferrocene-pyrazole sulfonamide derivatives have been synthesized to remove the overexpression of COX-II which was associated with carcinogenesis in different types of cancer. This suggests the potential link between inflammation and cancer. Thus, Ren et al.^222^ synthesized a series of novel ferrocene-pyrazolo sulfonamide derivatives comprising NO as potential inhibitors of COX-II. Most of the derivatives exhibited potent biological activities. For instance, CCDC (**MC4**) displayed potent COX-II inhibition (*IC*_*50*_ = 0.82 μM) and antiproliferative activities in Hela cells (*IC*_*50*_ = 0.34 μM) when compared to reference drug Celecoxib (*IC*_*50*_ = 0.38 and *IC*_*50*_ = 7.91 μM). Furthermore, most of the compounds were tested *in vitro* based on the release of a moderate amount of NO, which was associated with antiproliferative activities. **MC4** has shown antitumor activity in Hela cell xenograft mouse model when evaluated *in vivo*. Hence, these derivatives were very helpful in the future for the development of novel antitumors compounds (Figure 44).

Now, in recent days researchers are focused on developing novel metal-based compounds with better biological activities. Tabrizi et al.^223^ synthesized new cyclometalated ruthenium(II) complex [Ru(CCC-Nap)(Ibu)(PTA)] using various compounds like Ibuprofen (Ibu), 1,3,5-triaza-7-phosphaadamantane (PTA), and CCC-pincer containing naproxen moiety (CCC-Nap) as a ligand. The synthesized compounds have less cytotoxicity effects. Especially, the ruthenium complex (**MC5**) containing naproxen moiety of CCC-Nap and Ibuprofen was found to be twice as active as cisplatin (*IC*_*50*_ (0.9–1.32 μM) with a selective index of 63.90, which is better than the *S.I.* of corresponding free ligands Ibuprofen (*S.I.* 2.93) and CCC-Pincer (*S.I.* 34.17). Inhibition studies revealed that the Ru(II) complex has about 16- and 5-times stronger interactions with COX-II than free Ibu and CCC-Nap ligands, respectively. It has been reported that the Ru (II) complex increased the production of reactive oxygen species (ROS) by 10.7-fold compared to the control H_2_O_2_ (H_2_O_2_ as a positive control) in MCF-7 cells. With the help of molecular docking studies, these compounds were shown to make significant interactions with COX-II through van der Waals and electrostatic forces of attractions, and hydrogen bonding. The results suggest that a ruthenium-based complex is a promising strategy for designing novel compounds using NSAIDs against COX enzyme (Figure 44).

### 4.18 Natural Product as Anti-inflammatory Agent

The need of natural products as anti-inflammatory agents is increasing day by day to provide safe and significant biologically activity. For example, the concentrated and viscous aqueous extract of ripe carob was used as folk medicine for treating mouth inflammations in Arab countries. The extracts of natural products open a new area for the development of novel inhibitors. Moreover, natural products were also applied for safe and effective treatment of chronic inflammation.^224,225^ In the present manuscript, we incorporate different compounds of natural origin with potent anti-inflammatory activity.

**Figure 45:**
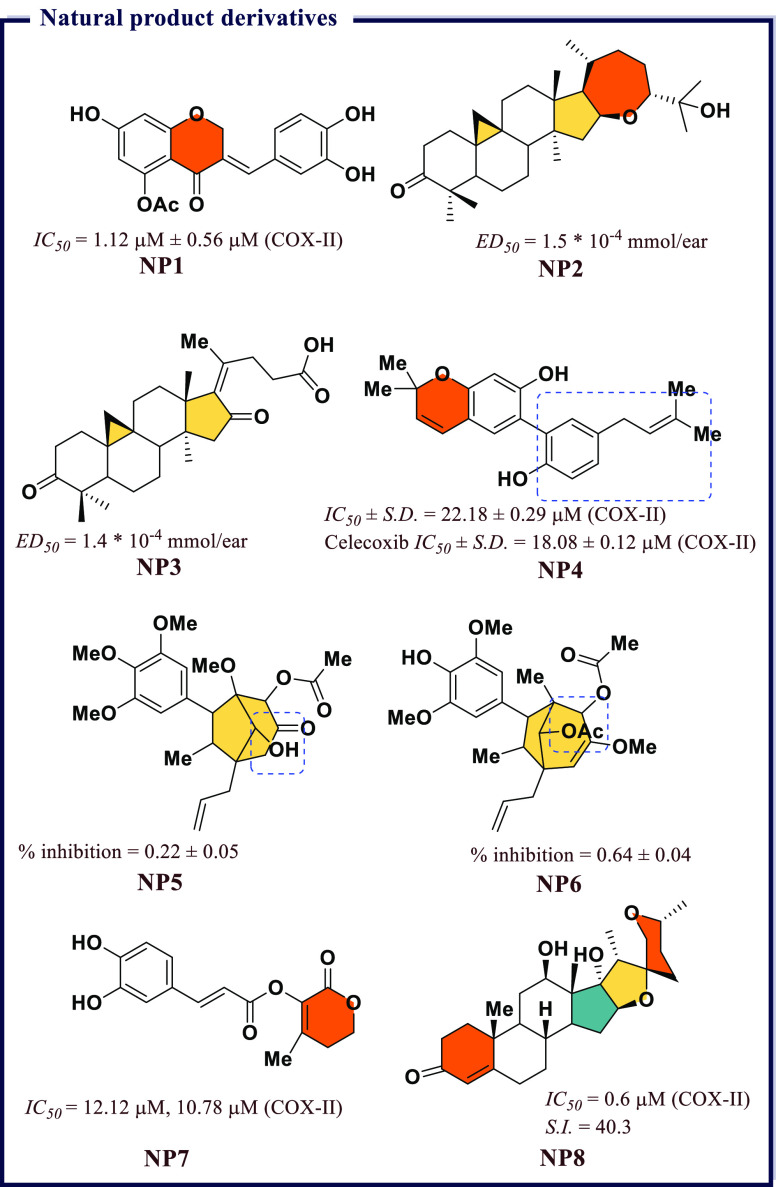
Natural product derivatives as potential COX-II inhibitors.

In various pathophysiological processes like inflammation and carcinogenesis, isoforms of COX-II and nitric oxide synthase were induced due to prostaglandins (PGs) and nitric oxide (NO). COX-II is not present in normal tissue but produced by pro-inflammatory cytokines and growth factor. Oxidation of a terminal guanidine nitrogen atom via NOS results in the production of NO. The major reason for the side effect was overexpression of NO. For the selective inhibition of COX-II and iNOS, methanolic extracts of natural product were screened to identify a new lead compound for the inhibition of these enzymes in LPS-stimulated RAW 264.7 cell, a murine macrophage cell line. Continuing after working in this direction, Raju Gautam et al.^226^ have extracted *n*-hexane and ethyl acetate from the plant *Dysophylla stellate*. It was found that these extracts inhibited edema at a dose of 0.5 mg/ear in TPA-induced ear edema assay in mice. A significant inhibitory activity was observed at 50 μg/mL against ABTS (COX-I = 85.42%, COX-II = 71.79%) and DPPH radical scavenging assay (COX-I = 71.79%, COX-II = 89.27%) due to the presence of flavonoids. But, in the case of *n*-hexane extract, no further activities were revealed (Figure 45).

Katsukawa et al.^227^ identified a chemical component from lemongrass oil (citral). It was found that citral was a suppressor of COX-II and activator of PPAR α and γ. However, the isomers of citral, geranial, and neral were not able to work as a pharmacological agent because of the low activity for both COX-II suppression and activation of PPAR α and γ. Here, citral has been reported to suppress both LPS induced COX-II mRNA and protein expression in human macrophages like U937 cells. It was also found that citral activated PPAR α and γ. Further, the study displayed that it also regulated the expression of COX-II and had a significant effect *in vivo* using PPARα knockout mice. The result indicated that the lemongrass oil had good anti-inflammatory activities.

Then, a new compound called imperatorin was isolated from the roots of glehnialittoralis by Huang et al.^228^*In vitro* and *in vivo* studies of this compound have shown it to possess concentration dependent inhibitory effect on NO production. Western blotting confirmed that the protein expression of COX-II and iNOS was blocked by the isolated compound. Further, imperatorin also increased the activities of other enzymes, namely catalases, superoxide dismutases, and glutathione peroxidases in paw edema. After that, the compound with anti-inflammatory activity was found to be decreased in the volume of paw edema after 4 and 5 h. Imperatorin, similar to Indomethacin, was also known to reduce neutrophil infiltration into sites of inflammation. It also has a significant role in the prevention of free radical formation, which leads to many diseases.

The compounds isolated from bulbs of *L. ovatifolia* were found to have toxic effects. Further investigation resulted in a new series of compounds isolated from *Ledebouria socialis* (Hyacinthaceae) by Waller et al.^229^ The most potent compound, which inhibits selectively COX-I (*IC*_*50*_ = 2.56 μM ± 1.2) and COX-II (*IC*_*50*_ of 1.12 μM ± 0.56 μM), was (*E*)-3-(3^`^,4^`^-dihydroxybenzylidene)-5-acetoxy-7-hydroxychroman-4-one(ovatifolionone acetate) (**NP1**). The structure of **NP1** was further evaluated using various spectroscopic techniques like NMR, IR, and UV. Some compounds, like ovatifolionone, a 3-benzylidene-4-chromanone homoisoflavanone, were isolated as a yellow powder from the EtOAc extract of *L. ovatifolia*. The compound was acetylated to aid purification and formed a monoacetate (5Ac). The molecular ion was not observed in the HRESIMS, but a peak at 299.0553 corresponding to a [M-OAc]^+^ ion was observed. The IR absorption bands at 3415, 1745, and 1638 cm^–1^ indicated hydroxyl, acetate, and ketone carbonyl stretches, respectively. These compounds have clinically relevant properties against COX-II (Figure 45).

Romero et al.^230^ determined the anti-inflammatory potential of cycloartenol-type triterpenes present in *Parthenium argentatum*. Good anti-inflammatory activity was observed from 2-*O*-tetradecanoylphorbol-3-acetate (TPA) when *in vivo* experiments were performed by induced edema model in mice. The compounds, *viz* argentatin B and argentatin A, were found showing a significant anti-inflammatory potential. They screened 13 derivatives of argentatins A and B for anti-inflammatory activity in the TPA-induced edema model in mice. The most active compounds obtained from Argentatin were 25-nor-cycloart-3 (**NP2**) and 16-dione-17-en-24-oic acid (**NP3**) with a significant *ED*_*50*_ values of 1.5 × 10^–4^ and 1.4 × 10^–4^ mmol/ear, respectively.

Modern clinical research indicates that *O. javanica* has effective therapeutic effects and relevant biological activities, i.e., hypertension, cerebrovascular diseases, antidiabetic, antihepatitis-B virus, and antifatigue. For example, Ma et al.^231^ isolated four novel biphenyl derivatives along with six known biphenyl derivatives from the aerial parts of *Oenanthe javanica* and showed that a few of them exhibited COX-II inhibition activity in the range from 22.18 ± 0.29 μM to 108.54 ± 0.42 μM. Especially, 1-(6′-hydroxy-3′-prenyl-phenyl)-10,11-dimethyl-2*H*-chromen-2-ol (**NP4**) exhibited the highest inhibition value against COX-II with a significant *IC*_*50*_ value of 22.18 ± 0.29 μM compared with the standard drug Celecoxib (*IC*_*50*_ = 18.08 ± 0.12 μM) (Figure 45).

Two new bicyclic octane neolignans obtained from *Aniba firmula* (Santos et al.)^232^ were also found revealing s significant anti-inflammatory potential. It was found that **NP5** and **NP6** have significant inhibitory activities determined using croton oil-induced ear edema as compared to reference drug dexamethasone. Moreover, **NP5** inhibits both neutrophil and edema generation in MPO assay and the level of PGE_2_ was one of the inflammatory mediators produced by COX enzyme. It is noteworthy to mention here that no inhibitory effect was observed in case of LOX pathway due to **NP5**. Further, the potent inhibition of **NP5** (0.22 ± 0.05) was observed using MPO as reference drug dexamethasone. But, **NP6** was not able to inhibit the inflammation cell recruitment (absorbance values: 0.64 ± 0.04) with the same assay. Thus, **NP5** has shown only antiedematogenic activity like NSAIDs. It was associated with higher efficiency and lesser side effect as compared to commonly used anti-inflammatory drugs (Figure 45).

Kang et al.^233^ isolated caffeoyloxy-5,6-dihydro-4-methyl-(2*H*)-pyran-2-one (CDMP), olinioside, caffeic acid, and 3-hydroxylup-12-en-28-oic acid from the leaves of *Oliniausambarensis*. The isolates that inhibited the LPS-triggered NO and PGE_2_ production in RAW 264.7 macrophages were assessed. It was found that a compound named CDMP (**NP7**) exhibited the most potent activity at micromolar range. CDMP also suppressed LPS-induced nuclear factor κB (NF-κB) by minimizing the p65 nuclear translocation through the phosphorylation and degradation of the inhibitory κBα (IκBα). Finally, down-regulation of several pro-inflammatory related genes was also proposed (Figure 45). Recently, the biological activity of triterpenes has also become an attraction. For example, Karim et al. isolated triterpenes from *Asparagus racemosus* and demonstrated their anti-inflammatory potential. Herein, a triterpenes derivative, *Asparacosin A* (**NP8**) (Figure 44), revealed significant COX-II inhibitory potential.

## 5 COX-II Selective Molecule Design

Computer-aided drug designing (CADD) is a valuable tool to design new drug molecules. By CADD, protein–ligand binding affinity and selectivity can be easily explored, and therefore, these studies are very useful to develop highly selective drug molecules. As COX-II selective inhibitors are particularly useful and under such a scenario, the role of CADD is quite admirable. Many of the CADD based methods are particularly useful in imparting COX-II selectivity in the molecule design. Both ligand-based and structure-based techniques help impart COX-II selectivity by using QM, MM, or a QM/MM hybrid approach.^234^ Quantitative structural activity relationship (QSAR) study is a ligand-based approach that can facilitate researchers in COX-II selective inhibitor design. The QSAR attempts to utilize the structural feature and activity profile of the existing COX-II selective ligands and create a statistical model of the same. Such QSAR models are quite helpful in predicting the COX-II selectivity of the designed molecules. Herein, several reports show the importance of QSAR in COX-II selective drug designing. In a report by Chaturvedi et al., QSAR for meclofenamic acid derivatives was explored, and flexibility of the molecule was found necessary toward COX-II selectivity.^235^ In a study by A. Jouyban et al., a QSAR model for the COX inhibitory potential of trans-stilbenoid diaryl compounds was achieved. The developed models efficiently predicted the COX-I/COX-II inhibitory potential and proficiently indicated the ligand’s selectivity toward COX-II protein.^236^ A study by Chaturvedi et al. revealed the essential structural features of 2,3-diaryl benzopyrans/pyrans for COX-II selective inhibitory potential. According to the current QSAR model, COX-II inhibitory activity was highly correlated with the lowest unoccupied molecular orbital (*E*_LUMO_), electronic descriptors, Dipole-Z, and hydrophobicity aspect of the molecule. The direct correlation of hydrophobicity/electron-withdrawing substituents at third aromatic with the activity was concluded from the current study. Moreover, the Z component was inversely correlated with its binding potential against the COX-II protein.^237^ Other reports show the role of QSAR/ligand-based drug designing in developing COX-II selective drug molecules.

Similarly, the structure-based approach can also be used to facilitate the COX-II selective inhibitor design. Herein, molecular docking simulation has provided us with a way to explore the binding affinity of the ligand against the drug-binding cavity of COX-I and COX-II protein. The difference in the binding cavities of both proteins can be further realized by carefully investigating the crystal structure of both proteins. Considering outcomes from the above steps, we can construct an ideal COX-II selective design. Similar strategies exist, and several successful studies are available in the literature. One such report is the research of Alban Arrault et al., whereby the research group has utilized molecular docking simulation and pharmacophore analysis studies and successfully explored the COX-II selective inhibitor molecules.^238^ Similarly, another study by Md. Jashim Uddin et al. also demonstrated the successful use of molecular docking simulation studies for COX-II selective inhibitor design. According to the report, the Celecoxib analogue with the meta-sulfonylazido group has COX-II selective tendencies because of its unique pattern of interaction with the receptor protein. The report has further demonstrated the critical binding role of the sulfonylazido (SO_2_N_3_) group within the cavity of the COX-II protein. Herein, SO_2_ oxygen showed H-binding interaction and the azide part responsible for electrostatic ion–ion interaction. In this case, the guanidino NH_2_ of Arg 531 was found accountable for the critical above-listed interactions.^239^

Molecular dynamics (MD) simulation studies have also imparted a significant role in designing and developing COX-II selective inhibitor molecules. MD simulation can include the protein’s flexibility aspect, lacking in the most common docking protocols. We can further realize the stability of the docked ligand–receptor complexes as a function of time. These studies are beneficial for discovering the individual interactions and microscopic events during the relative motion of ligand and protein when present in the bounded form. Precise understanding of the dynamic behavior of the ligand within the protein’s binding cavity is crucial for receptor-selective ligand design, say COX-II in our case. Therefore, MD simulation can play a vital role in designing and developing COX-II selective inhibitor molecules.^240^ Therefore, using MD-simulation protocols, best-docked molecules obtained from molecular docking studies can be further examined for their receptor binding potential.^241^ MDs results will provide us with the dynamic behavior of the ligand–receptor complex in the form of several representative frames. In this case, the MMGBSA binding energy calculations are convenient as we can use them for calculating the binding energy for the individual frame. Therefore, comparative MMGBSA binding energy studies with COX-I and COX-II are beneficial to studies in determining the COX-II selective inhibitor molecules.^242^

## 6 Chemical Spacing

Since several chemical scaffolds of COX inhibitors with wide range of activity profiles have been reported in the literature, we extended our review toward identifying the possibly best fit molecules out of molecules available in the literature. To achieve this, we performed chemical spacing analyses of the FDA-approved COX-inhibitors in the years 2015–2020.^243^ Chemical space is one of the vital concepts in drug discovery that correlates chemical pharmacophores with their drug-likeness involving key molecular descriptors.^244^ In this work, we performed our chemical spacing analyses based on a select set of descriptors including molecular weight (MW), topological polar surface area (TPSA), number of rotational bonds (nROTB), hydrogen bond donors (nHBDon), acceptors (nHBAcc), and implication of partition coefficient (AlogP). For each of these descriptors, a cutoff value was assigned; and, if a ligand falls within the preset cutoff values, then those compounds can be considered as having maximal drug-like attributes. For instance, a cutoff value of 500 g/mol was assigned for the MW descriptor.^245,246^ This is per the Lipinski rule and is applicable for small molecules only. Other descriptors are according to the Veber’s rule that defines TPSA and nROTB for a druggable candidate and is concerned with probability of an orally active or inactive candidate. The TPSA, that considers permeability across the biological membrane, should be less or equal to 140 Å^2^. The lesser the TPSA, the easier a molecule can cross the bio membrane. nROTB deals with flexibility of chemical moieties in the biological system, and the greater the nROTB, the greater will be the chances of off-target interaction of a drug candidate with a receptor. The acceptable cutoff for this parameter is the presence of 10 or less rotatable bonds to attain a good oral bioavailability and attain lesser off target interactions.^247^ Further, the nHBDon and nHBAcc in a drug candidate should be less than 5 and 10, respectively. These descriptors play a vital role for nHBAcc and play a vital role in drug binding and their electronic interactions with the receptor. The last descriptor, AlogP, defines the partition of drug candidate between the organic and aqueous phase. The range of −0.4 to +5.6 is acceptable for AlogP.^248^

The chemical space descriptors for FDA approved drugs from 2015–2020 are presented in Table 1.

**Table 1:** Key Descriptors and Their Numerical Values (in Comparison to Recommended Cutoffs) for the USFDA Approved Drugs from 2015–2020 under Various Categories

Category	Mean MW (Year 2015–20)	Mean TPSA (Year 2015–20)	Mean nROTB (Year 2015–20)	Mean nHBDon (Year 2015–20)	Mean nHBAcc (Year 2015–20)	Mean AlogP (Year 2015–20)
Anticancer drugs	503.7	103.9	7.3	2.4	8.1	–0.665
Neurological drugs	347.3	66.2	5.6	1.2	4.5	–0.009
Drugs for metabolic disorders	365.8	87.3	4.7	2.6	5.9	–0.057
Drugs for respiratory diseases	597.3	108.2	13	2	9	4.902
Anti-infective drugs	478.1	131.9	7.2	2.4	8.2	–0.373
Drugs for autoimmune disorders	433.1	93.8	6.8	2.2	5.6	–0.477
Cardiovascular drugs	484.8	140.1	12.7	3.6	8.8	–0.586
Average	458.6	104.5	8.2	2.3	7.2	0.390
Recommended cutoffs	<500 g/mol	≤140 Å^2^	≤10	≤5	≤10	–0.4 to +5.6

To find the corroboration and get an estimation of drug likeness of the reported COX inhibitors in literature, we analyzed the above-discussed descriptors using PUMA (Platform for Unified Molecular Analysis) online server version 1.^249^ The analysis presented us with the ranking of reported compounds (see SI, Table S1) on the basis of their drug likeliness. The top 5 compounds identified from the literature are presented in Table 2.

**Table 2:** Top 5 Best Fit Molecules Available in the Literature (During the Period of Analysis) Having Maximal Drug Likeliness Properties

Rank	DB	ID	MW	TPSA	nRotB	nHBDon	nHBAcc	ALogP
1	1,2,4-Trisubstitued pyrazole/pyrazoline	PYZ18	361.08	73.71	4	1	3	3.59
2	1,2,4-Trisubstitued pyrazole/pyrazoline	PYZ19	375.07	68.86	5	0	5	1.02
3	1,2,4-Trisubstitued pyrazole/pyrazoline	PYZ20	435.12	102.6	4	1	7	0.61
4	1,2,4-Trisubstitued pyrazole/pyrazoline	PYZ21	498.15	96.61	5	1	7	1.15
5	1,3,4- and 1,3,4,5-Substituted pyrazole	PYZ41	490.14	41.9	8	0	4	4.11

**Figure 46:**
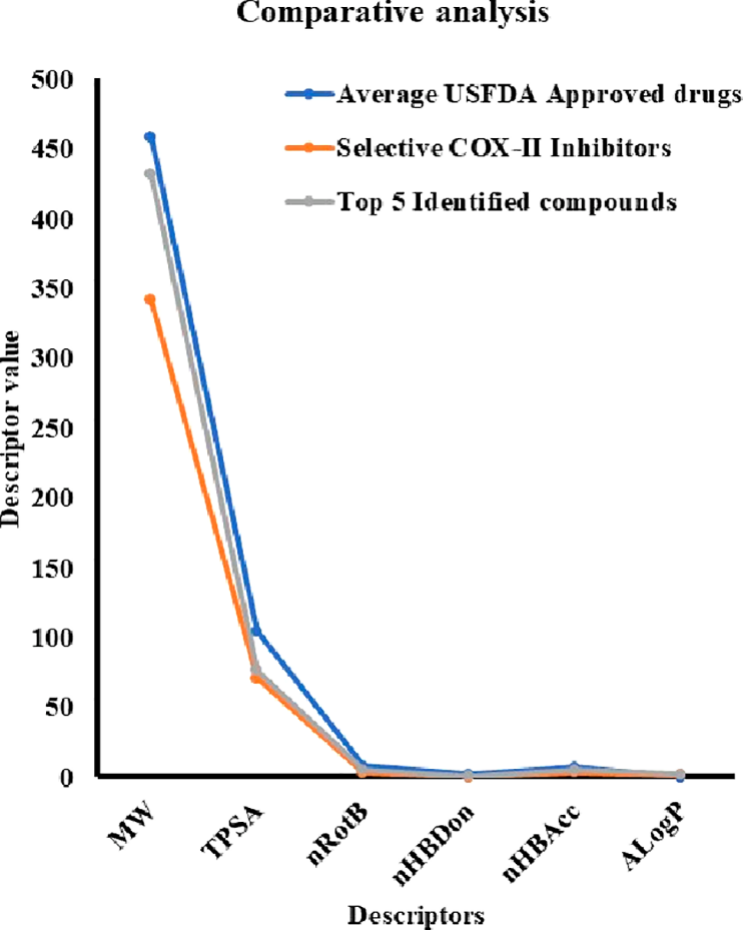
Plot suggesting a high correlation between USFDA approved drugs and reported synthetics as COX-II inhibitors.

We also performed a similar methodology for approved selective COX-II inhibitors, and the results are portrayed in Table 3. The profile compares (Table 4 and Figure 46) USFDA approved drugs, selective COX-II inhibitors, and top identified compounds (reported in literature). The analysis revealed (Figure 46) that the reported compounds as COX inhibitors have high similarity with USFDA approved drugs, thus presenting a strong plausibility to identify a hit lead for translational research leading to a clinical candidate in the future.

**Table 3:** Selective COX-II Inhibitors and Their Drug Likeness Index as Per the Descriptors Used

ID	MW	TPSA	nRotB	nHBDon	nHBAcc	ALogP
Celecoxib	381.07	84.14	4	1	5	2.15
Rofecoxib	314.06	68.82	3	0	4	1.64
Etoricoxib	358.05	67.24	3	0	4	1.76
Valdecoxib	313.07	64.11	3	0	2	1.65

**Table 4:** Comparative Analysis of Chemical Space Descriptors for USFDA Approved Drugs, Selective COX-II Inhibitors and Reported Molecules

Parameters	MW	TPSA	nRotB	nHBDon	nHBAcc	ALogP
Average USFDA approved drugs	458.59	104.49	8.20	2.33	7.18	0.39
Selective COX-II inhibitors	341.57	71.08	3.25	0.25	3.75	1.80
Top 5 identified compounds	431.92	76.74	5.2	0.6	5.2	2.10

Further we have also summarized the broad category of the pharmacophores reported in this work for their chemical spacing parameters. The analysis is summarized in Table 5.

**Table 5:** Summary of the Broad Category of the Pharmacophores Reported in This Work for Their Chemical Spacing Parameters

Broad category	*n*	MW	TPSA	nRotB	nHBDon	nHBAcc	ALogP
1,2,4-Trisubstitued pyrazole/pyrazoline	8	498.02	107.14	7.50	1.38	7.25	1.14
1,3,4- and 1,3,4,5-Substituted pyrazole	3	416.47	45.42	6.33	0.33	4.67	2.75
1,3,4-Substituted pyrazole	6	374.73	100.74	5.83	1.33	5.83	2.27
1,3,4,5-Substituted pyrazole	6	435.27	71.85	4.50	0.50	5.00	2.88
1,3,5-Susbtituted pyrazole	5	465.77	80.10	8.40	1.00	6.20	2.10
2,3-Substituted pyrazole	3	456.13	74.44	6.33	1.00	6.33	1.36
2,3,4,5-susbtituted thiazole and 2,5-substituted thiadiazole	2	511.55	153.28	5.00	2.00	7.50	2.10
3,4- and 3,4,5- Susbtituted isoxazole	3	347.44	78.14	6.00	0.00	3.67	0.67
Cinnoline linked pyrazole	2	355.06	62.01	2.50	0.00	5.50	1.15
Coumarin derivatives	11	370.66	95.36	5.73	1.36	6.18	0.40
COX/LOX inhibitors	3	293.09	47.98	3.67	0.67	4.00	1.38
Hybridized molecules	39	440.009	112.31	5.33	1.23	7.31	1.28
Isatin derivatives	3	400.74	97.80	4.67	1.33	6.67	0.16
Miscellaneous molecules	12	409.64	68.45	5.00	0.75	4.42	1.93
Molecules derived from natural products	8	402.21	81.38	4.13	1.63	5.50	0.71
Non heterocyclic compounds	8	307.51	48.83	6.63	0.75	2.75	2.73
Pyrazole/pyrazoline linked hydrazone, benzoimidazole, and benzothiazole	20	411.96	103.08	5.50	1.45	7.10	0.59
Selective COX-II inhibitors	8	300.57	62.67	3.63	0.75	3.63	1.59
Six membered exclude pyridine	17	402.63	95.57	5.76	1.47	5.65	1.04
Substituted pyrole and pyrrolidine	8	403.70	114.88	5.88	0.88	6.00	0.57
Substituted benzoxazoles derivatives	11	392.46	62.77	4.45	0.45	5.00	2.19
Substituted indole derivatives	17	501.62	76.40	6.94	1.06	5.88	1.33
Substituted non Heterocyclic compounds	15	344.19	68.22	5.60	1.47	4.53	1.55
Substituted phenyl linked thiazolidine	3	388.71	120.72	4.67	2.00	6.00	0.81
Tetrazole and triazole derivatives	11	395.36	117.43	6.27	1.00	7.91	0.38
Triazole linked pyrazole	2	395.61	99.09	4.00	0.50	7.50	0.45
Substituted quinoline derivatives	6	435.43	72.92	5.00	0.83	5.17	1.88

## 7 Conclusion

COX-II is an important target against inflammatory diseases. In this review, various scaffolds including pyrazole, isoxazole, oxadiazole, pyrrole, pyrrolidine, thiazole, thiazolidine, benzoxazole, isatin, coumarin, indole, quinolone, tetrazole, and triazole have been explored with COX-II inhibitory potential. Chemical spacing was performed to investigate the best lead compound having maximum drug likeness properties. **PYZ18** was observed as the best lead compound as a COX-II inhibitor. **PYZ18** can be a lead candidate for future drug design as a COX-II inhibitor.

## Supporting Information Available

Physiochemical parameters of COX inhibitors The Supporting Information is available free of charge at https://pubs.acs.org/doi/10.1021/acsomega.3c00692.

Physiochemical parameters of COX inhibitors (PDF)

## Abbreviations

INM: Indomethacin
COX-II: Cyclooxygenase-II
*U.I.*: Ulcer Index
*S.I.*: Selectivity Index
SAR: Structure–activity relationship
MVD: Molegro Virtual Docker
EWG: Electron withdrawing group
MTT: 3-(45-Dimethylthiazol-2-yl)-2,5-diphenyl tetrazolium bromide
EGFR: Epidermal growth factor receptor.
EDG: Electron donating group
NSAIDs: Nonsteroidal anti-inflammatory drugs
*IC*_*50*_: Inhibitory concentration
PGE_2_: Prostaglandin E_2_
2-AG: 2-Arachidonoylglycerol
AEA: Arachidonoylethanolamide
PGH_2_: Prostaglandin-H_2_
PGH_2_-G: Prostaglandin-glycerylesters
PGH_2_-EA: Prostaglandin-ethanolamides
5-LOX: 5-Arachidonate 5-lipoxygenase
*ED*_*50*_: Median effective dose
SD.: Standard Deviation

## References

[ref1] Greten F. R.; Grivennikov S. I. Inflammation and cancer: Triggers, mechanisms and consequences. Immunity 2019, 51 (1), 27–41. 10.1016/j.immuni.2019.06.025. 31315034 PMC6831096

[ref2] Bullock J.; Rizvi S. A. A.; Saleh A. M.; et al. Rheumatoid arthritis: A brief overview of the treatment. Med. Princ Pract. 2019, 27 (6), 501–507. 10.1159/000493390. PMC6422329 30173215

[ref3] Duan L.; Rao X.; Sigdel K. R. Regulation of inflammation in autoimmune Disease. J. Immunol. Res. 2019, 2019, 7403796 10.1155/2019/7403796. 30944837 PMC6421792

[ref4] Willerson J. T.; Ridker P. M. Inflammation as a cardiovascular risk factor. Circulation 2004, 109 (21_suppl_1), II-2–II-10. 10.1161/01.CIR.0000129535.04194.38. 15173056

[ref5] Razavi S. M.; Khayatan D.; Arab Z. N.; Momtaz S.; Zare K.; Jafari R. M.; Dehpour A. R.; Abdolghaffari A. H. Licofelone, a potent COX/5-LOX inhibitor and a novel option for treatment of neurological disorders. Prostaglandins Other Lipid Mediat 2021, 157, 106587 10.1016/j.prostaglandins.2021.106587. 34517113

[ref6] Chandrasekharan N. V.; Dai H.; Roos K. L. T.; Evanson N. K.; Tomsik J.; Elton T. S.; Simmons D. L. COX-3, a cyclooxygenase-1 variant inhibited by acetaminophen and other analgesic/antipyretic drugs: cloning, structure, and expression. Proc. Natl. Acad. Sci. U. S. A. 2002, 99 (21), 13926–13931. 10.1073/pnas.162468699. 12242329 PMC129799

[ref7] Molina-Sánchez P.; Del Campo L.; Esteban V.; Rius C.; Chèvre R.; Fuster J. J.; Ferrer M.; Redondo J. M.; Andrés V. Defective p27 phosphorylation at serine 10 affects vascular reactivity and increases abdominal aortic aneurysm development via Cox-2 activation. J. Mol. Cell. Cardiol. 2018, 116, 5–15. 10.1016/j.yjmcc.2018.01.010. 29408196

[ref8] Litalien C.; Jacqz-Aigrain E. Risks and benefits of nonsteroidal anti-inflammatory drugs in children. Paediatr. Drugs 2001, 3 (11), 817–858. 10.2165/00128072-200103110-00004. 11735667

[ref9] McEvoy L.; Carr D. F.; Pirmohamed M. Pharmacogenomics of NSAID-induced upper gastrointestinal toxicity. Frontiers in Pharmacology 2021, 12, 684162 10.3389/fphar.2021.684162. 34234675 PMC8256335

[ref10] Celotti F.; Laufer S. Anti-inflammatory drugs: new multitarget compounds to face an old problem. The dual inhibition concept. Pharmacol. Res. 2001, 43 (5), 429–436. 10.1006/phrs.2000.0784. 11394934

[ref11] Green G. A. Understanding NSAIDs: From aspirin to COX-II. Clin. Cornerstone 2001, 3 (5), 50–59. 10.1016/S1098-3597(01)90069-9. 11464731

[ref12] Mahboubi Rabbani S. M. I.; Zarghi A. Selective COX-II inhibitors as anticancer agents: A patent review (2014–2018). Expert Opin. Ther. Pat. 2019, 29 (6), 407–427. 10.1080/13543776.2019.1623880. 31132889

[ref13] Abraham N. S.; EL-Serag H. B.; Hartman C.; RIichardson P.; Deswal A. Cyclooxygenase-II selectivity of non-steroidal anti-inflammatory drugs and the risk of myocardial infarction and cerebrovascular accident. Alimentary Pharmacology & Therapeutics 2007, 25 (8), 913–924. 10.1111/j.1365-2036.2007.03292.x. 17402995

[ref14] Ulbrich H.; Fiebich B.; Dannhardt G. Cyclooxygenase-1/2 (COX-I/COX-II) and 5-lipoxygenase (5-LOX) inhibitors of the 6,7-diaryl-2,3–1*H*-dihydropyrrolizine type. Eur. J. Med. Chem. 2002, 37 (12), 953–959. 10.1016/S0223-5234(02)01418-6. 12660020

[ref15] Laube M.; Tondera C.; Sharma S. K.; Bechmann N.; Pietzsch F.-J.; Pigorsch A.; Köckerling M.; Wuest F.; Pietzsch J.; Kniess T. 2,3-Diaryl-substituted indole based COX-II inhibitors as leads for imaging tracer development. RSC Adv. 2014, 4 (73), 38726–38742. 10.1039/C4RA05650G.

[ref16] Tewari A. K.; Singh V. P.; Yadav P.; Gupta G.; Singh A.; Goel R. K.; Shinde P.; Mohan C. G. Synthesis, biological evaluation and molecular modeling study of pyrazole derivatives as selective COX-II inhibitors and anti-inflammatory agents. Bioorg. Chem. 2014, 56, 8–15. 10.1016/j.bioorg.2014.05.004. 24893208

[ref17] Hinz B.; Brune K. Cyclooxygenase-II 10 years later. Journal of pharmacology and experimental therapeutics 2002, 300 (2), 367–375. 10.1124/jpet.300.2.367. 11805193

[ref18] Xie W.; Chipman J. G.; Robertson D. L.; Erikson R.; Simmons D. L. Expression of a mitogen-responsive gene encoding prostaglandin synthase is regulated by mRNA splicing. Proc. Natl. Acad. Sci. U. S. A. 1991, 88 (7), 2692–2696. 10.1073/pnas.88.7.2692. 1849272 PMC51304

[ref19] Picot D.; Loll P. J.; Garavito R. M. The X-ray crystal structure of the membrane protein prostaglandin H2 synthase-1. Nature 1994, 367 (6460), 243–249. 10.1038/367243a0. 8121489

[ref20] Miciaccia M.; Belviso B. D.; Iaselli M.; Cingolani G.; Ferorelli S.; Cappellari M.; Loguercio Polosa P.; Perrone M. G.; Caliandro R.; Scilimati A. Three-dimensional structure of human cyclooxygenase (hCOX)-I. Sci. Rep. 2021, 11 (1), 4312 10.1038/s41598-021-83438-z. 33619313 PMC7900114

[ref21] Orlando B. J.; Malkowski M. G. Crystal structure of Rofecoxib bound to human cyclooxygenase-II. Acta Crystallographica Section F 2016, 72 (10), 772–776. 10.1107/S2053230X16014230. PMC5053162 27710942

[ref22] Garavito R. M.; Malkowski M. G.; DeWitt D. L. The structures of prostaglandin endoperoxide H synthases-1 and-2. Prostaglandins & other lipid mediators 2002, 68, 129–152. 10.1016/S0090-6980(02)00026-6. 12432914

[ref23] Marnett L. J.; Rowlinson S. W.; Goodwin D. C.; Kalgutkar A. S.; Lanzo C. A. Arachidonic acid oxygenation by COX-I and COX-II Mechanisms of catalysis and inhibition. J. Biol. Chem. 1999, 274 (33), 22903–22906. 10.1074/jbc.274.33.22903. 10438452

[ref24] Smith W. L.; Murphy R. C. The eicosanoids: cyclooxygenase, lipoxygenase and epoxygenase pathways. In Biochemistry of lipids, lipoproteins and membranes; Elsevier, 2016; pp 259–296.

[ref25] Turini M. E.; DuBois R. N. Cyclooxygenase-II: A therapeutic target. Annual review of medicine 2002, 53 (1), 35–57. 10.1146/annurev.med.53.082901.103952. 11818462

[ref26] Blobaum A. L.; Marnett L. J. Structural and functional basis of cyclooxygenase inhibition. J. Med. Chem. 2007, 50 (7), 1425–1441. 10.1021/jm0613166. 17341061

[ref27] Rouzer C. A.; Marnett L. J. Cyclooxygenases: Structural and functional insights. Journal of lipid research 2009, 50 (Suppl), S29–34. 10.1194/jlr.R800042-JLR200. 18952571 PMC2674713

[ref28] Abdel-Aziz A. A.-M.; El-Azab A. S.; AlSaif N. A.; Alanazi M. M.; El-Gendy M. A.; Obaidullah A. J.; Alkahtani H. M.; Almehizia A. A.; Al-Suwaidan I. A. Synthesis, anti-inflammatory, cytotoxic, and COX-I/II inhibitory activities of cyclic imides bearing 3-benzenesulfonamide, oxime, and β-phenylalanine scaffolds: a molecular docking study. Journal of Enzyme Inhibition and Medicinal Chemistry 2020, 35 (1), 610–621. 10.1080/14756366.2020.1722120. 32013633 PMC7034070

[ref29] Sobolewski C.; Cerella C.; Dicato M.; Ghibelli L.; Diederich M. The role of cyclooxygenase-II in cell proliferation and cell death in human malignancies. International journal of cell biology 2010, 2010, 168–184. 10.1155/2010/215158. PMC2841246 20339581

[ref30] Vane J. R.; Bakhle Y. S.; Botting R. M. Cyclooxygenases I and II. Annual review of pharmacology and toxicology 1998, 38, 97–120. 10.1146/annurev.pharmtox.38.1.97. 9597150

[ref31] Rouzer C. A.; Marnett L. J. Cyclooxygenases: structural and functional insights. Journal of lipid research 2009, 50, S29–S34. 10.1194/jlr.R800042-JLR200. 18952571 PMC2674713

[ref32] Rouzer C. A.; Marnett L. J. Structural and chemical biology of the interaction of cyclooxygenase with substrates and non-steroidal anti-inflammatory drugs. Chem. Rev. 2020, 120 (15), 7592–7641. 10.1021/acs.chemrev.0c00215. 32609495 PMC8253488

[ref33] Garavito R. M.; Mulichak A. M. The structure of mammalian cyclooxygenases. Annual review of biophysics and biomolecular structure 2003, 32, 183–206. 10.1146/annurev.biophys.32.110601.141906. 12574066

[ref34] Smith W. L.; Dewitt D. L. Prostaglandin endoperoxide H synthases-1 and −2. Advances in immunology 1996, 62, 167–215. 10.1016/S0065-2776(08)60430-7. 8781269

[ref35] Bekhit A. A.; Abdel-Aziem T. Design, synthesis and biological evaluation of some pyrazole derivatives as anti-inflammatory-antimicrobial agents. Bioorg. Med. Chem. 2004, 12 (8), 1935–1945. 10.1016/j.bmc.2004.01.037. 15051061

[ref36] Bekhit A. A.; Fahmy H. T. Y.; Rostom S. A. F.; Bekhit A. E.-D. A. Synthesis and biological evaluation of some thiazolylpyrazole derivatives as dual anti-inflammatory antimicrobial agents. Eur. J. Med. Chem. 2010, 45 (12), 6027–6038. 10.1016/j.ejmech.2010.10.001. 20970223

[ref37] Eren G.; Ünlü S.; Nuñez M.-T.; Labeaga L.; Ledo F.; Entrena A.; Banoglu E.; Costantino G.; Sahin M. F. Synthesis, biological evaluation, and docking studies of novel heterocyclic diaryl compounds as selective COX-2 inhibitors. Bioorg. Med. Chem. 2010, 18 (17), 6367–6376. 10.1016/j.bmc.2010.07.009. 20692174

[ref38] Sharma P. K.; Kumar S.; Kumar P.; Kaushik P.; Kaushik D.; Dhingra Y.; Aneja K. R. Synthesis and biological evaluation of some pyrazolylpyrazolines as anti-inflammatory-antimicrobial agents. Eur. J. Med. Chem. 2010, 45 (6), 2650–5. 10.1016/j.ejmech.2010.01.059. 20171763

[ref39] Abdelgawad M. A.; Bakr R. B.; Omar H. A. Design, synthesis and biological evaluation of some novel benzothiazole/benzoxazole and/or benzimidazole derivatives incorporating a pyrazole scaffold as antiproliferative agents. Bioorg. Chem. 2017, 74, 82–90. 10.1016/j.bioorg.2017.07.007. 28772160

[ref40] Abdelgawad M. A.; Labib M. B.; Ali W. A. M.; Kamel G.; Azouz A. A.; El-Nahass E. L. S. Design, synthesis, analgesic, anti-inflammatory activity of novel pyrazolones possessing aminosulfonyl pharmacophore as inhibitors of COX-II/5-LOX enzymes: Histopathological and docking studies. Bioorg. Chem. 2018, 78, 103–114. 10.1016/j.bioorg.2018.03.011. 29550530

[ref41] Moneer A. A.; Mohammed K. O.; El-Nassan H. B. Synthesis of novel substituted thiourea and benzimidazole derivatives containing a pyrazolone ring as anti-Inflammatory agents. Chem. Biol. Drug Des. 2016, 87 (5), 784–793. 10.1111/cbdd.12712. 26684979

[ref42] Abdellatif K. R. A.; Abdelgawad M. A.; Labib M. B.; Zidan T. H. Synthesis, cyclooxygenase inhibition, anti-inflammatory evaluation and ulcerogenic liability of novel triarylpyrazoline derivatives as selective COX-II inhibitors. Bioorg. Med. Chem. Lett. 2015, 25 (24), 5787–5791. 10.1016/j.bmcl.2015.10.047. 26546221

[ref43] Abdellatif K. R. A.; Fadaly W. A. A.; Elshaier Y. A. M. M.; Ali W. A. M.; Kamel G. M. Non-acidic 1,3,4-*tri*substituted-pyrazole derivatives as lonazolac analogs with promising COX-II selectivity, anti-inflammatory activity and gastric safety profile. Bioorg. Chem. 2018, 77, 568–578. 10.1016/j.bioorg.2018.02.018. 29475165

[ref44] Abdellatif K. R. A.; Abdelall E. K. A.; Labib M. B.; Fadaly W. A. A.; Zidan T. H. Design, synthesis of Celecoxib-tolmetin drug hybrids as selective and potent COX-II inhibitors. Bioorg. Chem. 2019, 90, 103029 10.1016/j.bioorg.2019.103029. 31212177

[ref45] Pavase L. S.; Mane D. V.; Baheti K. G. Anti-inflammatory exploration of sulfonamide containing diaryl pyrazoles with promising COX-II Selectivity and enhanced Gastric Safety Profile. J. Heterocycl. Chem. 2018, 55 (4), 913–922. 10.1002/jhet.3118.

[ref46] Hwang S. H.; Wagner K. M.; Morisseau C.; Liu J.-Y.; Dong H.; Wecksler A. T.; Hammock B. D. Synthesis and structure-activity relationship studies of urea-containing pyrazoles as dual inhibitors of cyclooxygenase-II and soluble epoxide hydrolase. J. Med. Chem. 2011, 54 (8), 3037–3050. 10.1021/jm2001376. 21434686 PMC3281519

[ref47] Chandna N.; Kumar S.; Kaushik P.; Kaushik D.; Roy S. K.; Gupta G. K.; Jachak S. M.; Kapoor J. K.; Sharma P. K. Synthesis of novel Celecoxib analogues by bioisosteric replacement of sulfonamide as potent anti-inflammatory agents and cyclooxygenase inhibitors. Bioorg. Med. Chem. 2013, 21 (15), 4581–4590. 10.1016/j.bmc.2013.05.029. 23769654

[ref48] Chandna N.; Kapoor J. K.; Grover J.; Bairwa K.; Goyal V.; Jachak S. M. Pyrazolylbenzyltriazoles as cyclooxygenase inhibitors: synthesis and biological evaluation as dual anti-inflammatory and antimicrobial agents. New J. Chem. 2014, 38 (8), 3662–3672. 10.1039/C4NJ00226A.

[ref49] Chen Z.; Wang Z.-C.; Yan X.-Q.; Wang P.-F.; Lu X.-Y.; Chen L.-W.; Zhu H.-L.; Zhang H.-W. Design, synthesis, biological evaluation and molecular modeling of dihydropyrazole sulfonamide derivatives as potential COX-I/COX-II inhibitors. Bioorg. Med. Chem. Lett. 2015, 25 (9), 1947–1951. 10.1016/j.bmcl.2015.03.022. 25866240

[ref50] Qiu H.-Y.; Wang P.-F.; Li Z.; Ma J.-T.; Wang X.-M.; Yang Y.-H.; Zhu H.-L. Synthesis of dihydropyrazole sulphonamide derivatives that act as anti-cancer agents through COX-II inhibition. Pharmacol. Res. 2016, 104, 86–96. 10.1016/j.phrs.2015.12.025. 26723906

[ref51] Gurram B.; Zhang S.; Li M.; Li H.; Xie Y.; Cui H.; Du J.; Fan J.; Wang J.; Peng X. Celecoxib conjugated fluorescent probe for identification and discrimination of cyclooxygenase-II enzyme in cancer cells. Anal. Chem. 2018, 90 (8), 5187–5193. 10.1021/acs.analchem.7b05337. 29587478

[ref52] Taher E. S.; Ibrahim T. S.; Fares M.; Al-Mahmoudy A. M. M.; Radwan A. F.; Orabi K. Y.; El-Sabbagh O. I. Novel benzenesulfonamide and 1,2-benzisothiazol-3(2*H*)-one-1,1-dioxide derivatives as potential selective COX-II inhibitors. Eur. J. Med. Chem. 2019, 171, 372–382. 10.1016/j.ejmech.2019.03.042. 30928709

[ref53] Faidallah H. M.; Rostom S. A. F. Synthesis, anti-Inflammatory activity, and Cox-I/II inhibition profile of some novel non-acidic polysubstituted pyrazoles and pyrano[2,3-c]pyrazoles. Arch. Pharm. 2017, 350 (5), 1700025 10.1002/ardp.201700025. 28370254

[ref54] El-Sayed M. A. A.; Abdel-Aziz N. I.; Abdel-Aziz A. A. M.; El-Azab A. S.; Asiri Y. A.; ElTahir K. E. H. Design, synthesis and biological evaluation of substituted hydrazone and pyrazole derivatives as selective COX-II inhibitors: Molecular docking study. Bioorg. Med. Chem. 2011, 19 (11), 3416–3424. 10.1016/j.bmc.2011.04.027. 21570309

[ref55] El-Sayed M. A.; Abdel-Aziz N. I.; Abdel-Aziz A. A.; El-Azab A. S.; ElTahir K. E. Synthesis, biological evaluation and molecular modeling study of pyrazole and pyrazoline derivatives as selective COX-II inhibitors and anti-inflammatory agents. Part 2. Bioorg. Med. Chem. 2012, 20 (10), 3306–16. 10.1016/j.bmc.2012.03.044. 22516672

[ref56] Murahari M.; Mahajan V.; Neeladri S.; Kumar M. S.; Mayur Y. C. Ligand based design and synthesis of pyrazole based derivatives as selective COX-II inhibitors. Bioorg. Chem. 2019, 86, 583–597. 10.1016/j.bioorg.2019.02.031. 30782576

[ref57] Chavan H. V.; Bandgar B. P.; Adsul L. K.; Dhakane V. D.; Bhale P. S.; Thakare V. N.; Masand V. Design, synthesis, characterization and anti-inflammatory evaluation of novel pyrazole amalgamated flavones. Bioorg. Med. Chem. Lett. 2013, 23 (5), 1315–1321. 10.1016/j.bmcl.2012.12.094. 23357629

[ref58] Hassan G. S.; Abdel Rahman D. E.; Abdelmajeed E. A.; Refaey R. H.; Alaraby Salem M.; Nissan Y. M. New pyrazole derivatives: Synthesis, anti-inflammatory activity, cycloxygenase inhibition assay and evaluation of mPGES. Eur. J. Med. Chem. 2019, 171, 332–342. 10.1016/j.ejmech.2019.03.052. 30928706

[ref59] Qiu K.-M.; Yan R.; Xing M.; Wang H.-H.; Cui H.-E.; Gong H.-B.; Zhu H.-L. Synthesis, biological evaluation and molecular modeling of dihydro-pyrazolyl-thiazolinone derivatives as potential COX-II inhibitors. Bioorg. Med. Chem. 2012, 20 (22), 6648–6654. 10.1016/j.bmc.2012.09.021. 23062711

[ref60] Li Z.; Wang Z.-C.; Li X.; Abbas M.; Wu S.-Y.; Ren S.-Z.; Liu Q.-X.; Liu Y.; Chen P.-W.; Duan Y.-T.; Lv P.-C.; Zhu H.-L. Design, synthesis and evaluation of novel diaryl-1,5-diazoles derivatives bearing morpholine as potent dual COX-II/5-LOX inhibitors and antitumor agents. Eur. J. Med. Chem. 2019, 169, 168–184. 10.1016/j.ejmech.2019.03.008. 30877972

[ref61] Ren S.-Z.; Wang Z.-C.; Zhu X.-H.; Zhu D.; Li Z.; Shen F.-Q.; Duan Y.-T.; Cao H.; Zhao J.; Zhu H.-L. Design and biological evaluation of novel hybrids of 1, 5-diarylpyrazole and Chrysin for selective COX-II inhibition. Bioorg. Med. Chem. 2018, 26 (14), 4264–4275. 10.1016/j.bmc.2018.07.022. 30031652

[ref62] Yan X.-Q.; Wang Z.-C.; Zhang B.; Qi P.-F.; Li G.-G.; Zhu H.-L. Dihydropyrazole derivatives containing benzo oxygen heterocycle and sulfonamide moieties selectively and potently inhibit COX-II: design, synthesis, and anti-colon cancer activity evaluation. Molecules 2019, 24 (9), 1685 10.3390/molecules24091685. 31052167 PMC6539903

[ref63] Dube P. N.; Bule S. S.; Mokale S. N.; Kumbhare M. R.; Dighe P. R.; Ushir Y. V. Synthesis and biologic evaluation of substituted 5-methyl-2-phenyl-1H-pyrazol-3(2*H*)-one derivatives as Selective COX-II Inhibitors: Molecular Docking Study. Chem. Biol. Drug Des. 2014, 84 (4), 409–419. 10.1111/cbdd.12324. 24636540

[ref64] Alegaon S. G.; Hirpara M. B.; Alagawadi K. R.; Hullatti K. K.; Kashniyal K. Synthesis of novel pyrazole-thiadiazole hybrid as potential potent and selective cyclooxygenase-II (COX-II) inhibitors. Bioorg. Med. Chem. Lett. 2014, 24 (22), 5324–5329. 10.1016/j.bmcl.2014.08.062. 25444084

[ref65] Alegaon S. G.; Alagawadi K. R.; Garg M. K.; Dushyant K.; Vinod D. 1,3,4-*Tri*substituted pyrazole analogues as promising anti-inflammatory agents. Bioorg. Chem. 2014, 54, 51–59. 10.1016/j.bioorg.2014.04.001. 24793214

[ref66] Harras M. F.; Sabour R.; Alkamali O. M. Discovery of new non-acidic lonazolac analogues with COX-II selectivity as potent anti-inflammatory agents. MedChemComm 2019, 10 (10), 1775–1788. 10.1039/C9MD00228F. 31803395 PMC6844278

[ref67] Manvar D.; Pelliccia S.; La Regina G.; Famiglini V.; Coluccia A.; Ruggieri A.; Anticoli S.; Lee J.-C.; Basu A.; Cevik O.; Nencioni L.; Palamara A. T.; Zamperini C.; Botta M.; Neyts J.; Leyssen P.; Kaushik-Basu N.; Silvestri R. New 1-phenyl-5-(1*H*-pyrrol-1-yl)-1*H*-pyrazole-3-carboxamides inhibit hepatitis C virus replication *via* suppression of cyclooxygenase-II. Eur. J. Med. Chem. 2015, 90, 497–506. 10.1016/j.ejmech.2014.11.042. 25483263

[ref68] Ragab F. A.; Abdel Gawad N. M.; Georgey H. H.; Said M. F. Synthesis of novel 1,3,4-*tri*substituted pyrazoles as anti-inflammatory and analgesic agents. Eur. J. Med. Chem. 2013, 63, 645–654. 10.1016/j.ejmech.2013.03.005. 23567953

[ref69] Bandgar B. P.; Chavan H. V.; Adsul L. K.; Thakare V. N.; Shringare S. N.; Shaikh R.; Gacche R. N. Design, synthesis, characterization and biological evaluation of novel pyrazole integrated benzophenones. Bioorg. Med. Chem. Lett. 2013, 23 (3), 912–916. 10.1016/j.bmcl.2012.10.031. 23290048

[ref70] Tonk K. R.; Bawa S.; Kumar D. Therapeutic potential of cinnoline core: a comprehensive review. Mini Rev. Med. Chem. 2020, 20 (3), 196–218. 10.2174/1389557519666191011095858. 31660825

[ref71] Bhardwaj A.; Kaur J.; Wuest M.; Wuest F. *In situ* click chemistry generation of cyclooxygenase-II inhibitors. Nat. Commun. 2017, 8 (1), 1–1. 10.1038/s41467-016-0009-6. 28232747 PMC5431875

[ref72] Zimecki M.; Bachor U.; Maczyński M. Isoxazole derivatives as regulators of immune functions. Molecules 2018, 23 (10), 2724 10.3390/molecules23102724. 30360408 PMC6222914

[ref73] Joy M.; Elrashedy A. A.; Mathew B.; Pillay A. S.; Mathews A.; Dev S.; Soliman M. E. S.; Sudarsanakumar C. Discovery of new class of methoxy carrying isoxazole derivatives as COX-II inhibitors: Investigation of a detailed molecular dynamics study. J. Mol. Struct. 2018, 1157, 19–28. 10.1016/j.molstruc.2017.11.109.

[ref74] Perrone m. g.; Vitale P.; Panella A.; Ferorelli S.; Contino M.; Lavecchia A.; Scilimati A. Isoxazole-based-scaffold inhibitors targeting cyclooxygenases (COXs). ChemMedChem. 2016, 11, 1172–1187. 10.1002/cmdc.201500439. 27136372

[ref75] El-Sayed N. A.; Nour M. S.; Salem M. A.; Arafa R. K. New oxadiazoles with selective-COX-II and EGFR dual inhibitory activity: Design, synthesis, cytotoxicity evaluation and in silico studies. Eur. J. Med. Chem. 2019, 183, 111693 10.1016/j.ejmech.2019.111693. 31539778

[ref76] Akhter M.; Akhter N.; Alam M. M.; Zaman M. S.; Saha R.; Kumar A. Synthesis and biological evaluation of 2,5-disubstituted 1,3,4-oxadiazole derivatives with both COX and LOX inhibitory activity. J. Enzyme Inhib. Med. Chem. 2011, 26 (6), 767–776. 10.3109/14756366.2010.550890. 21314246

[ref77] Palkar M. B.; Singhai A. S.; Ronad P. M.; Vishwanathswamy A. H. M.; Boreddy T. S.; Veerapur V. P.; Shaikh M. S.; Rane R. A.; Karpoormath R. Synthesis, pharmacological screening and *in silico* studies of new class of Diclofenac analogues as a promising anti-inflammatory agents. Bioorg. Med. Chem. 2014, 22 (10), 2855–2866. 10.1016/j.bmc.2014.03.043. 24751552

[ref78] Mishra A. P.; Bajpai A.; Chandra S. A comprehensive review on the screening models for the pharmacological assessment of antiulcer drugs. Current clinical pharmacology 2019, 14, 175–196. 10.2174/1574884714666190312143846. 30864527

[ref79] Beiranvand M. A review of the most common in vivo models of stomach ulcers and natural and synthetic anti-ulcer compounds: A comparative systematic study. Phytomedicine Plus 2022, 2, 100264 10.1016/j.phyplu.2022.100264.

[ref80] Ganguly A. K. A method for quantitative assessment of experimentally produced ulcers in the stomach of albino rats. Experientia 1969, 25, 1224–1224. 10.1007/BF01900290. 5357845

[ref81] Adinortey M. B.; Ansah C.; Galyuon I.; Nyarko A. In vivo models used for evaluation of potential antigastroduodenal ulcer agents. Ulcers 2013, 2013, 2013 10.1155/2013/796405.

[ref82] Grover J.; Bhatt N.; Kumar V.; Patel N.; Gondaliya J.; Sobhia M.; Bhutani K.; Jachak S. 2,5-Diaryl-1,3,4-Oxadiazoles as Selective COX-2 Inhibitors and Anti-Inflammatory Agents. RSC Adv. 2015, 5, 45535 10.1039/C5RA01428J.

[ref83] Abd-Ellah H.; Abdel-Aziz M.; Shoman M.; Beshr E.; Kaoud T.; Ahmed A.-S. New 1,3,4-Oxadiazole/Oxime hybrids: Design, Synthesis, anti-inflammatory, COX inhibitory activities and Ulcerogenic liability. Bioorg. Chem. 2017, 74, 15–29. 10.1016/j.bioorg.2017.06.003. 28738249

[ref84] Puttaswamy N.; Malojiao V. H.; Mohammed Y. H. E.; Sherapura A.; Prabhakar B. T.; Khanum S. A. Synthesis and amelioration of inflammatory paw edema by novel benzophenone appended oxadiazole derivatives by exhibiting cyclooxygenase-II antagonist activity. Biomed. Pharmacother. 2018, 103, 1446–1455. 10.1016/j.biopha.2018.04.167. 29864929

[ref85] Said Fatahala S.; Hasabelnaby S.; Goudah A.; Mahmoud G. I.; Helmy Abd-El Hameed R. Pyrrole and fused pyrrole compounds with bioactivity against inflammatory mediators. Molecules 2017, 22 (3), 461 10.3390/molecules22030461. 28304349 PMC6155178

[ref86] Dannhardt G.; Kiefer W.; Kraemer G.; Maehrlein S.; Nowe U.; Fiebich B. The Pyrrole Moiety as a Template for COX-1/COX-2 Inhibitors. Eur. J. Med. Chem. 2000, 35, 499–510. 10.1016/S0223-5234(00)00150-1. 10889329

[ref87] Biava M.; Porretta G. C.; Poce G.; Battilocchio C.; Alfonso S.; Rovini M.; Valenti S.; Giorgi G.; Calderone V.; Martelli A.; Testai L.; Sautebin L.; Rossi A.; Papa G.; Ghelardini C.; Di Cesare Mannelli L.; Giordani A.; Anzellotti P.; Bruno A.; Patrignani P.; Anzini M. Novel analgesic/anti-inflammatory agents: diarylpyrrole acetic esters endowed with nitric oxide releasing properties. J. Med. Chem. 2011, 54 (22), 7759–7771. 10.1021/jm200715n. 21992176

[ref88] Reale A.; Brogi S.; Chelini A.; Paolino M.; Di Capua A.; Giuliani G.; Cappelli A.; Giorgi G.; Chemi G.; Grillo A.; Valoti M.; Sautebin L.; Rossi A.; Pace S.; La Motta C.; Di Cesare Mannelli L.; Lucarini E.; Ghelardini C.; Anzini M. Synthesis, biological evaluation and molecular modeling of novel selective COX-II inhibitors: sulfide, sulfoxide, and sulfone derivatives of 1,5-diarylpyrrol-3-substituted scaffold. Bioorg. Med. Chem. 2019, 27 (19), 115045 10.1016/j.bmc.2019.115045. 31427145

[ref89] Firke S. D.; Bari S. B. Synthesis, biological evaluation and docking study of maleimide derivatives bearing benzenesulfonamide as selective COX-II inhibitors and anti-inflammatory agents. Bioorg. Med. Chem. 2015, 23 (17), 5273–5281. 10.1016/j.bmc.2015.07.070. 26277757

[ref90] Jan M. S.; Ahmad S.; Hussain F.; Ahmad A.; Mahmood F.; Rashid U.; Abid O.-u.-R.; Ullah F.; Ayaz M.; Sadiq A. Design, synthesis, *in-vitro*, *in-vivo* and *in-silico* studies of pyrrolidine-2,5-dione derivatives as multitarget anti-inflammatory agents. Eur. J. Med. Chem. 2020, 186, 111863 10.1016/j.ejmech.2019.111863. 31740050

[ref91] Kim K. J.; Choi M. J.; Shin J.-S.; Kim M.; Choi H.-E.; Kang S. M.; Jin J. H.; Lee K.-T.; Lee J. Y. Synthesis, biological evaluation, and docking analysis of a novel family of 1-methyl-1H-pyrrole-2,5-diones as highly potent and selective cyclooxygenase-II (COX-II) inhibitors. Bioorg. Med. Chem. Lett. 2014, 24 (8), 1958–1962. 10.1016/j.bmcl.2014.02.074. 24656662

[ref92] Al-Suwaidan I. A.; Alanazi A. M.; El-Azab A. S.; Al-Obaid A. M.; ElTahir K. E. H.; Maarouf A. R.; Abu El-Enin M. A.; Abdel-Aziz A. A. M. Molecular design, synthesis and biological evaluation of cyclic imides bearing benzenesulfonamide fragment as potential COX-II inhibitors. Part 2. Bioorg. Med. Chem. Lett. 2013, 23 (9), 2601–2605. 10.1016/j.bmcl.2013.02.107. 23528298

[ref93] Dawood K. M.; Eldebss T. M.; El-Zahabi H. S.; Yousef M. H.; Metz P. Synthesis of some new pyrazole-based 1,3-thiazoles and 1,3,4-thiadiazoles as anticancer agents. Eur. J. Med. Chem. 2013, 70, 740–9. 10.1016/j.ejmech.2013.10.042. 24231309

[ref94] Abdelazeem A. H.; Gouda A. M.; Omar H. A.; Tolba M. F. Design, synthesis and biological evaluation of novel diphenylthiazole-based cyclooxygenase inhibitors as potential anticancer agents. Bioorg Chem. 2014, 57, 132–141. 10.1016/j.bioorg.2014.10.001. 25462989

[ref95] Abdu-Allah H. H. M.; Abdelmoez A. A. B.; Tarazi H.; El-Shorbagi A. A.; El-Awady R. Conjugation of 4-aminosalicylate with thiazolinones afforded non-cytotoxic potent *in vitro* and *in vivo* anti-inflammatory hybrids. Bioorg Chem. 2020, 94, 103378 10.1016/j.bioorg.2019.103378. 31677858

[ref96] Abdellatif K. R.; Abdelgawad M. A.; Elshemy H. A.; Alsayed S. S. Design, synthesis and biological screening of new 4-thiazolidinone derivatives with promising COX-II selectivity, anti-inflammatory activity and gastric safety profile. Bioorg Chem. 2016, 64, 1–12. 10.1016/j.bioorg.2015.11.001. 26561742

[ref97] Ma L.; Xie C.; Ma Y.; Liu J.; Xiang M.; Ye X.; Zheng H.; Chen Z.; Xu Q.; Chen T.; Chen J.; Yang J.; Qiu N.; Wang G.; Liang X.; Peng A.; Yang S.; Wei Y.; Chen L. Synthesis and biological evaluation of novel 5-benzylidenethiazolidine-2,4-dione derivatives for the treatment of inflammatory diseases. J. Med. Chem. 2011, 54 (7), 2060–8. 10.1021/jm1011534. 21381754

[ref98] El-Gamal M. I.; Bayomi S. M.; El-Ashry S. M.; Said S. A.; Abdel-Aziz A. A.; Abdel-Aziz N. I. Synthesis and anti-inflammatory activity of novel (substituted)benzylidene acetone oxime ether derivatives: molecular modeling study. Eur. J. Med. Chem. 2010, 45 (4), 1403–14. 10.1016/j.ejmech.2009.12.041. 20079558

[ref99] Arfaie S.; Zarghi A. Design, synthesis and biological evaluation of new (E)- and (Z)-1,2,3-triaryl-2-propen-1-ones as selective COX-II inhibitors. Eur. J. Med. Chem. 2010, 45 (9), 4013–7. 10.1016/j.ejmech.2010.05.058. 20691338

[ref100] Genovese S.; Curini M.; Gresele P.; Corazzi T.; Epifano F. Inhibition of COX-I activity and COX-II expression by 3-(4’-geranyloxy-3′-methoxyphenyl)-2-trans propenoic acid and its semi-synthetic derivatives. Bioorg. Med. Chem. Lett. 2011, 21 (19), 5995–8. 10.1016/j.bmcl.2011.07.040. 21843935

[ref101] Lee B.; Kwak J. H.; Huang S. W.; Jang J. Y.; Lim S.; Kwak Y. S.; Lee K.; Kim H. S.; Han S. B.; Hong J. T.; Lee H.; Song S.; Seo S. Y.; Jung J. K. Design and synthesis of 4-O-methylhonokiol analogs as inhibitors of cyclooxygenase-II (COX-II) and PGF(1) production. Bioorg. Med. Chem. 2012, 20 (9), 2860–8. 10.1016/j.bmc.2012.03.028. 22494844

[ref102] Windsor M. A.; Hermanson D. J.; Kingsley P. J.; Xu S.; Crews B. C.; Ho W.; Keenan C. M.; Banerjee S.; Sharkey K. A.; Marnett L. J. Substrate-selective inhibition of cyclooxygenase-II: development and evaluation of achiral Profen probes. ACS Med. Chem. Lett. 2012, 3 (9), 759–763. 10.1021/ml3001616. 22984634 PMC3441040

[ref103] Bano S.; Javed K.; Ahmad S.; Rathish I. G.; Singh S.; Chaitanya M.; Arunasree K. M.; Alam M. S. Synthesis of some novel chalcones, flavanones and flavones and evaluation of their anti-inflammatory activity. Eur. J. Med. Chem. 2013, 65, 51–9. 10.1016/j.ejmech.2013.04.056. 23693150

[ref104] Ghatak S.; Vyas A.; Misra S.; O’Brien P.; Zambre A.; Fresco V. M.; Markwald R. R.; Swamy K. V.; Afrasiabi Z.; Choudhury A.; Khetmalas M.; Padhye S. Novel di-tertiary-butyl phenylhydrazones as dual cyclooxygenase-2/5-lipoxygenase inhibitors: synthesis, COX/LOX inhibition, molecular modeling, and insights into their cytotoxicities. Bioorg. Med. Chem. Lett. 2014, 24 (1), 317–24. 10.1016/j.bmcl.2013.11.015. 24295787

[ref105] Grover J.; Kumar V.; Singh V.; Bairwa K.; Sobhia M. E.; Jachak S. M. Synthesis, biological evaluation, molecular docking and theoretical evaluation of ADMET properties of nepodin and chrysophanol derivatives as potential cyclooxygenase (COX-I, COX-II) inhibitors. Eur. J. Med. Chem. 2014, 80, 47–56. 10.1016/j.ejmech.2014.04.033. 24763362

[ref106] Kang S. M.; Lee J.; Jin J. H.; Kim M.; Lee S.; Lee H. H.; Shin J. S.; Lee K. T.; Lee J. Y. Synthesis and PGE(2) production inhibition of s-triazine derivatives as a novel scaffold in RAW 264.7 macrophage cells. Bioorg. Med. Chem. Lett. 2014, 24 (23), 5418–22. 10.1016/j.bmcl.2014.10.031. 25453800

[ref107] Yang Y. X.; Zheng L. T.; Shi J. J.; Gao B.; Chen Y. K.; Yang H. C.; Chen H. L.; Li Y. C.; Zhen X. C. Synthesis of 5-alpha-cholestan-6-one derivatives and their inhibitory activities of NO production in activated microglia: discovery of a novel neuroinflammation inhibitor. Bioorg. Med. Chem. Lett. 2014, 24 (4), 1222–7. 10.1016/j.bmcl.2013.12.055. 24456901

[ref108] Silva T.; Borges F.; Edraki N.; Alizadeh M.; Miri R.; Saso L.; Firuzi O. Hydroxycinnamic acid as a novel scaffold for the development of cyclooxygenase-II inhibitors. RSC Adv. 2015, 5 (72), 58902–58911. 10.1039/C5RA08692B.

[ref109] Gorantla V.; Gundla R.; Jadav S. S.; Anugu S. R.; Chimakurthy J.; Nidasanametla S. K.; Korupolu R. Molecular hybrid design, synthesis and biological evaluation of N-phenyl sulfonamide linked N-acyl hydrazone derivatives functioning as COX-II inhibitors: new anti-inflammatory, anti-oxidant and anti-bacterial agents. New J. Chem. 2017, 41 (22), 13516–13532. 10.1039/C7NJ03332J.

[ref110] El-Nagar M. K. S.; Abdu-Allah H. H. M.; Salem O. I. A.; Kafafy A. N.; Farghaly H. S. M. Novel N-substituted 5-aminosalicylamides as dual inhibitors of cyclooxygenase and 5-lipoxygenase enzymes: Synthesis, biological evaluation and docking study. Bioorg Chem. 2018, 78, 80–93. 10.1016/j.bioorg.2018.02.023. 29550533

[ref111] Navarro L.; Rosell G.; Sanchez S.; Boixareu N.; Pors K.; Pouplana R.; Campanera J. M.; Pujol M. D. Synthesis and biological properties of aryl methyl sulfones. Bioorg. Med. Chem. 2018, 26 (14), 4113–4126. 10.1016/j.bmc.2018.06.038. 29980364

[ref112] Kar S.; Ramamoorthy G.; Sinha S.; Ramanan M.; Pola J. K.; Golakoti N. R.; Nanubolu J. B.; Sahoo S. K.; Dandamudi R. B.; Doble M. Synthesis of diarylidenecyclohexanone derivatives as potential anti-inflammatory leads against COX-II/mPGES1 and 5-LOX. New J. Chem. 2019, 43 (23), 9012–9020. 10.1039/C9NJ00726A.

[ref113] Ribeiro D.; Proenca C.; Varela C.; Janela J.; Tavares da Silva E. J.; Fernandes E.; Roleira F. M. F. New phenolic cinnamic acid derivatives as selective COX-II inhibitors. Design, synthesis, biological activity and structure-activity relationships. Bioorg Chem. 2019, 91, 103179 10.1016/j.bioorg.2019.103179. 31404794

[ref114] Asghar A.; Yousuf M.; Mubeen H.; Nazir R.; Haruna K.; Onawole A. T.; Rasheed L. Synthesis, spectroscopic characterization, molecular docking and theoretical studies (DFT) of *N*-(4-aminophenylsulfonyl)-2-(4-isobutylphenyl) propanamide having potential enzyme inhibition applications. Bioorg. Med. Chem. 2019, 27 (12), 2397–2404. 10.1016/j.bmc.2019.01.012. 30683553

[ref115] Elhenawy A. A.; Al-Harbi L. M.; El-Gazzar M. A.; Khowdiary M. M.; Ouidate A.; Alosaimi A. M.; Elhamid Salim A. Naproxenylamino acid derivatives: Design, synthesis, docking, QSAR and anti-inflammatory and analgesic activity. Biomed Pharmacother 2019, 116, 109024 10.1016/j.biopha.2019.109024. 31150990

[ref116] Senkardes S.; Han M. I.; Kulabas N.; Abbak M.; Cevik O.; Kucukguzel I.; Kucukguzel S. G. Synthesis, molecular docking and evaluation of novel sulfonyl hydrazones as anticancer agents and COX-II inhibitors. Mol. Divers 2020, 24 (3), 673–689. 10.1007/s11030-019-09974-z. 31302853

[ref117] Ampati S.; Jukanti R.; Sagar V.; Ganta R.; Manda S. Synthesis and *in vivo* anti-inflammatory activity of a novel series of benzoxazole derivatives. Der Chemica Sinica 2010, 1 (3), 157–168.

[ref118] Seth K.; Garg S. K.; Kumar R.; Purohit P.; Meena V. S.; Goyal R.; Banerjee U. C.; Chakraborti A. K. 2-(2-Arylphenyl)benzoxazole as a novel anti-Inflammatory scaffold: synthesis and biological evaluation. ACS Med. Chem. Lett. 2014, 5 (5), 512–6. 10.1021/ml400500e. 24900871 PMC4027739

[ref119] Mayank; Kaur N.; Singh N. Structural insights and influence of V599 mutations on the overall dynamics of BRAF protein against its kinase domains. Integr Biol. (Camb) 2018, 10 (10), 646–657. 10.1039/C8IB00095F. 30229251

[ref120] Lamie P. F.; Philoppes J. N.; Rarova L. Design, synthesis, and biological evaluation of novel 1,2-diaryl-4-substituted-benzylidene-5(4*H*)-imidazolone derivatives as cytotoxic agents and COX-II/LOX inhibitors. Arch Pharm. (Weinheim) 2018, 351 (3–4), e1700311 10.1002/ardp.201700311. 29400411

[ref121] Kaur A.; Pathak D. P.; Sharma V.; Wakode S. Synthesis, molecular docking, and pharmacological evaluation of *N*-(2-(3,5-dimethoxyphenyl)benzoxazole-5-yl)benzamide derivatives as selective COX-II inhibitors and anti-inflammatory agents. Arch Pharm. (Weinheim) 2018, 351 (6), e1800008 10.1002/ardp.201800008. 29741797

[ref122] Yatam S.; Jadav S. S.; Gundla R.; Gundla K. P.; Reddy G. M.; Ahsan M. J.; Chimakurthy J. Design, synthesis and biological evaluation of 2 (((5-aryl-1,2,4-oxadiazol-3-yl)methyl)thio)benzo[*d*]oxazoles: New anti-inflammatory and antioxidant agents. ChemistrySelect 2018, 3 (37), 10305–10310. 10.1002/slct.201801558.

[ref123] Lahari K.; Sundararajan R. Design and synthesis of novel isatin derivatives as potent analgesic, anti-inflammatory and antimicrobial agents. J. Chem. Sci. 2020, 132 (1), 94 10.1007/s12039-020-01795-0.

[ref124] Abo-Ashour M. F.; Eldehna W. M.; Nocentini A.; Bonardi A.; Bua S.; Ibrahim H. S.; Elaasser M. M.; Krystof V.; Jorda R.; Gratteri P.; Abou-Seri S. M.; Supuran C. T. 3-Hydrazinoisatin-based benzenesulfonamides as novel carbonic anhydrase inhibitors endowed with anticancer activity: Synthesis, *in vitro* biological evaluation and *in silico* insights. Eur. J. Med. Chem. 2019, 184, 111768 10.1016/j.ejmech.2019.111768. 31629164

[ref125] Al-Qaisi J. A.; Alhussainy T. M.; Qinna N. A.; Matalka K. Z.; Al-Kaissi E. N.; Muhi-Eldeen Z. A. Synthesis and pharmacological evaluation of aminoacetylenic isoindoline-1,3-dione derivatives as anti-inflammatory agents. Arab J. Chem. 2014, 7 (6), 1024–1030. 10.1016/j.arabjc.2010.12.030.

[ref126] Revankar H. M.; Bukhari S. N.; Kumar G. B.; Qin H. L. Coumarins scaffolds as COX inhibitors. Bioorg Chem. 2017, 71, 146–159. 10.1016/j.bioorg.2017.02.001. 28222891

[ref127] Silvan A. M.; Abad M. J.; Bermejo P.; Sollhuber M.; Villar A. Anti-inflammatory activity of coumarins from Santolina oblongifolia. J. Nat. Prod 1996, 59 (12), 1183–5. 10.1021/np960422f. 8988605

[ref128] Melagraki G.; Afantitis A.; Igglessi-Markopoulou O.; Detsi A.; Koufaki M.; Kontogiorgis C.; Hadjipavlou-Litina D. J. Synthesis and evaluation of the antioxidant and anti-inflammatory activity of novel coumarin-3-aminoamides and their alpha-lipoic acid adducts. Eur. J. Med. Chem. 2009, 44 (7), 3020–6. 10.1016/j.ejmech.2008.12.027. 19232783

[ref129] Vasconcelos J. F.; Teixeira M. M.; Barbosa-Filho J. M.; Agra M. F.; Nunes X. P.; Giulietti A. M.; Ribeiro-Dos-Santos R.; Soares M. B. Effects of umbelliferone in a murine model of allergic airway inflammation. Eur. J. Pharmacol. 2009, 609 (1–3), 126–31. 10.1016/j.ejphar.2009.03.027. 19289114

[ref130] Sandhya B.; Giles D.; Mathew V.; Basavarajaswamy G.; Abraham R. Synthesis, pharmacological evaluation and docking studies of coumarin derivatives. Eur. J. Med. Chem. 2011, 46 (9), 4696–701. 10.1016/j.ejmech.2011.07.013. 21816516

[ref131] Dawood D. H.; Batran R. Z.; Farghaly T. A.; Khedr M. A.; Abdulla M. M. New coumarin derivatives as potent selective COX-II inhibitors: Synthesis, anti-Inflammatory, QSAR, and molecular modeling studies. Arch Pharm. (Weinheim) 2015, 348 (12), 875–88. 10.1002/ardp.201500274. 26462142

[ref132] Rayar A. M.; Lagarde N.; Martin F.; Blanchard F.; Liagre B.; Ferroud C.; Zagury J. F.; Montes M.; Sylla-Iyarreta Veitia M. New selective cyclooxygenase-II inhibitors from cyclocoumarol: Synthesis, characterization, biological evaluation and molecular modeling. Eur. J. Med. Chem. 2018, 146, 577–587. 10.1016/j.ejmech.2018.01.054. 29407982

[ref133] Kulkarni R. C.; Madar J. M.; Shastri S. L.; Shaikh F.; Naik N. S.; Chougale R. B.; Shastri L. A.; Joshi S. D.; Dixit S. R.; Sunagar V. A. Green synthesis of coumarin-pyrazolone hybrids: *In vitro* anticancer and anti-inflammatory activities and their computational study on COX-II enzyme. Chem. Data Coll. 2018, 17–18, 497–506. 10.1016/j.cdc.2018.11.004.

[ref134] Radwan M. A.; Ragab E. A.; Sabry N. M.; El-Shenawy S. M. Synthesis and biological evaluation of new 3-substituted indole derivatives as potential anti-inflammatory and analgesic agents. Bioorg. Med. Chem. 2007, 15 (11), 3832–41. 10.1016/j.bmc.2007.03.024. 17395469

[ref135] Kalgutkar A. S.; Marnett A. B.; Crews B. C.; Remmel R. P.; Marnett L. J. Ester and amide derivatives of the nonsteroidal antiinflammatory drug, Indomethacin, as selective cyclooxygenase-II inhibitors. J. Med. Chem. 2000, 43 (15), 2860–70. 10.1021/jm000004e. 10956194

[ref136] Lai Y.; Ma L.; Huang W.; Yu X.; Zhang Y.; Ji H.; Tian J. Synthesis and biological evaluation of 3-[4-(amino/methylsulfonyl)phenyl]methylene-indolin-2-one derivatives as novel COX-I/II and 5-LOX inhibitors. Bioorg. Med. Chem. Lett. 2010, 20 (24), 7349–53. 10.1016/j.bmcl.2010.10.056. 21055929

[ref137] Harrak Y.; Casula G.; Basset J.; Rosell G.; Plescia S.; Raffa D.; Cusimano M. G.; Pouplana R.; Pujol M. D. Synthesis, anti-inflammatory activity, and *in vitro* antitumor effect of a novel class of cyclooxygenase inhibitors: 4-(aryloyl)phenyl methyl sulfones. J. Med. Chem. 2010, 53 (18), 6560–71. 10.1021/jm100398z. 20804197

[ref138] Huang Y.; Zhang B.; Li J.; Liu H.; Zhang Y.; Yang Z.; Liu W. Design, synthesis, biological evaluation and docking study of novel indole-2-amide as anti-inflammatory agents with dual inhibition of COX and 5-LOX. Eur. J. Med. Chem. 2019, 180, 41–50. 10.1016/j.ejmech.2019.07.004. 31299586

[ref139] Hayashi S.; Sumi Y.; Ueno N.; Murase A.; Takada J. Discovery of a novel COX-II inhibitor as an orally potent anti-pyretic and anti-inflammatory drug: design, synthesis, and structure-activity relationship. Biochem. Pharmacol. 2011, 82 (7), 755–68. 10.1016/j.bcp.2011.06.036. 21741371

[ref140] Estevao M. S.; Carvalho L. C.; Freitas M.; Gomes A.; Viegas A.; Manso J.; Erhardt S.; Fernandes E.; Cabrita E. J.; Marques M. M. Indole based cyclooxygenase inhibitors: synthesis, biological evaluation, docking and NMR screening. Eur. J. Med. Chem. 2012, 54, 823–33. 10.1016/j.ejmech.2012.06.040. 22796043

[ref141] Kaur J.; Bhardwaj A.; Huang Z.; Knaus E. E. N-1 and C-3 substituted indole Schiff bases as selective COX-II inhibitors: synthesis and biological evaluation. Bioorg. Med. Chem. Lett. 2012, 22 (6), 2154–9. 10.1016/j.bmcl.2012.01.130. 22361134

[ref142] Kadirvel M.; Abudalal A. S.; Rajendran R.; Gbaj A.; Demonacos C.; Freeman S. Myo-inositol esters of Indomethacin as COX-II inhibitors. Carbohydr. Res. 2012, 355, 13–8. 10.1016/j.carres.2012.04.008. 22578527

[ref143] Gerbino P. P. Emerging evidence in nsaid pharmacology: Important considerations for product selection. Am. J. Manag Care 2015, 21, S139–S147. 26168321

[ref144] Chennamaneni S.; Zhong B.; Lama R.; Su B. COX inhibitors Indomethacin and Sulindac derivatives as antiproliferative agents: synthesis, biological evaluation, and mechanism investigation. Eur. J. Med. Chem. 2012, 56, 17–29. 10.1016/j.ejmech.2012.08.005. 22940705

[ref145] Uddin M. J.; Crews B. C.; Ghebreselasie K.; Marnett L. J. Design, synthesis, and structure-activity relationship studies of fluorescent inhibitors of cycloxygenase-II as targeted optical imaging agents. Bioconjug Chem. 2013, 24 (4), 712–23. 10.1021/bc300693w. 23488616 PMC3630741

[ref146] Laube M.; Gassner C.; Sharma S. K.; Gunther R.; Pigorsch A.; Konig J.; Kockerling M.; Wuest F.; Pietzsch J.; Kniess T. Diaryl-substituted (dihydro)pyrrolo[3,2,1-*hi*]indoles, a class of potent COX-II Inhibitors with tricyclic Core Structure. J. Org. Chem. 2015, 80 (11), 5611–24. 10.1021/acs.joc.5b00537. 25909690

[ref147] Laube M.; Gassner C.; Kniess T.; Pietzsch J. Synthesis and cyclooxygenase inhibition of sulfonamide-substituted (dihydro)pyrrolo[3,2,1-*hi*]indoles and their potential prodrugs. Molecules 2019, 24 (20), 3807 10.3390/molecules24203807. 31652609 PMC6832141

[ref148] Wang B.; Fan J.; Wang X.; Zhu H.; Wang J.; Mu H.; Peng X. A Nile blue based infrared fluorescent probe: Imaging tumors that over-express cyclooxygenase-II. Chem. Commun. (Camb) 2015, 51 (4), 792–5. 10.1039/C4CC08915D. 25424129

[ref149] Sever B.; Altintop M. D.; Kus G.; Ozkurt M.; Ozdemir A.; Kaplancikli Z. A. Indomethacin based new triazolothiadiazine derivatives: Synthesis, evaluation of their anticancer effects on T98 human glioma cell line related to COX-II inhibition and docking studies. Eur. J. Med. Chem. 2016, 113, 179–86. 10.1016/j.ejmech.2016.02.036. 26927686

[ref150] Naaz F.; Preeti Pallavi M. C.; Shafi S.; Mulakayala N.; Shahar Yar M.; Sampath Kumar H. M. 1,2,3-triazole tethered Indole-3-glyoxamide derivatives as multiple inhibitors of 5-LOX, COX-II & tubulin: Their anti-proliferative & anti-inflammatory activity. Bioorg Chem. 2018, 81, 1–20. 10.1016/j.bioorg.2018.07.029. 30081353

[ref151] Kaur S.; Kumari P.; Singh G.; Bhatti R.; Singh P. Design and synthesis of aza-/oxa heterocycle-based conjugates as novel anti-inflammatory agents targeting cyclooxygenase-II. ACS Omega 2018, 3 (5), 5825–5845. 10.1021/acsomega.8b00445. 30023927 PMC6044720

[ref152] Ju Z.; Su M.; Hong J.; La Kim E.; Moon H. R.; Chung H. Y.; Kim S.; Jung J. H. Design of balanced COX inhibitors based on anti-inflammatory and/or COX-II inhibitory ascidian metabolites. Eur. J. Med. Chem. 2019, 180, 86–98. 10.1016/j.ejmech.2019.07.016. 31301566

[ref153] Abdel-Aziz A. A.; Angeli A.; El-Azab A. S.; Hammouda M. E. A.; El-Sherbeny M. A.; Supuran C. T. Synthesis and anti-inflammatory activity of sulfonamides and carboxylates incorporating trimellitimides: Dual cyclooxygenase/carbonic anhydrase inhibitory actions. Bioorg Chem. 2019, 84, 260–268. 10.1016/j.bioorg.2018.11.033. 30508771

[ref154] Anderson G. J. Quinolone Antimicrobial Agents, 3rd Edition. Emerg. Infect. Dis. 2004, 10 (6), 1177a–1177. 10.3201/eid1006.040025.

[ref155] Solomon V. R.; Lee H. Quinoline as a privileged scaffold in cancer drug discovery. Curr. Med. Chem. 2011, 18 (10), 1488–508. 10.2174/092986711795328382. 21428893

[ref156] Kaur K.; Jain M.; Reddy R. P.; Jain R. Quinolines and structurally related heterocycles as antimalarials. Eur. J. Med. Chem. 2010, 45 (8), 3245–64. 10.1016/j.ejmech.2010.04.011. 20466465

[ref157] Tiglani D.; Salahuddin S.; Mazumder A.; Kumar R.; Mishra S. Synthetic approaches and biological activities of quinoline derivatives: A Review. Universal Journal of Pharmaceutical Research 2021, 27, 4605–4613. 10.31838/ijpr/2021.13.01.582.

[ref158] Chaaban I.; Rizk O. H.; Ibrahim T. M.; Henen S. S.; El-Khawass E. M.; Bayad A. E.; El-Ashmawy I. M.; Nematalla H. A. Synthesis, anti-inflammatory screening, molecular docking, and COX-I,II/-5-LOX inhibition profile of some novel quinoline derivatives. Bioorg Chem. 2018, 78, 220–235. 10.1016/j.bioorg.2018.03.023. 29602046

[ref159] Zarghi A.; Ghodsi R. Design, synthesis and biological evaluation of ketoprofen analogs as potent cyclooxygenase-II inhibitors. Bioorg. Med. Chem. 2010, 18 (16), 5855–60. 10.1016/j.bmc.2010.06.094. 20650641

[ref160] Ghodsi R.; Zarghi A.; Daraei B.; Hedayati M. Design, synthesis and biological evaluation of new 2,3-diarylquinoline derivatives as selective cyclooxygenase-II inhibitors. Bioorg. Med. Chem. 2010, 18 (3), 1029–33. 10.1016/j.bmc.2009.12.060. 20061161

[ref161] Abdelrahman M. H.; Youssif B. G. M.; Abdelgawad M. A.; Abdelazeem A. H.; Ibrahim H. M.; Moustafa A.; Treamblu L.; Bukhari S. N. A. Synthesis, biological evaluation, docking study and ulcerogenicity profiling of some novel quinoline-2-carboxamides as dual COXs/LOX inhibitors endowed with anti-inflammatory activity. Eur. J. Med. Chem. 2017, 127, 972–985. 10.1016/j.ejmech.2016.11.006. 27837994

[ref162] Chaaban I.; Rizk O. H.; Ibrahim T. M.; Henen S. S.; El-Khawass E. M.; Bayad A. E.; El-Ashmawy I. M.; Nematalla H. A. Synthesis, anti-inflammatory screening, molecular docking, and COX-I,II/-5-LOX inhibition profile of some novel quinoline derivatives. Bioorg. Chem. 2018, 78, 220–235. 10.1016/j.bioorg.2018.03.023. 29602046

[ref163] Rajasekaran A.; Rajagopal K. A. Synthesis of some novel triazole derivatives as anti-nociceptive and anti-inflammatory agents. Acta Pharm. 2009, 59 (3), 355–64. 10.2478/v10007-009-0026-7. 19819831

[ref164] Al-Hourani B. J.; Sharma S. K.; Suresh M.; Wuest F. Novel 5-substituted *1H*-tetrazoles as cyclooxygenase-II (COX-II) inhibitors. Bioorg. Med. Chem. Lett. 2012, 22 (6), 2235–8. 10.1016/j.bmcl.2012.01.093. 22341941

[ref165] Shafi S.; Alam M. M.; Mulakayala N.; Mulakayala C.; Vanaja G.; Kalle A. M.; Pallu R.; Alam M. S. Synthesis of novel 2-mercapto benzothiazole and 1,2,3-triazole based bis-heterocycles: their anti-inflammatory and anti-nociceptive activities. Eur. J. Med. Chem. 2012, 49, 324–33. 10.1016/j.ejmech.2012.01.032. 22305614

[ref166] Kaur J.; Bhardwaj A.; Sharma S. K.; Wuest F. 1,4-Diaryl-substituted triazoles as cyclooxygenase-II inhibitors: Synthesis, biological evaluation and molecular modeling studies. Bioorg. Med. Chem. 2013, 21 (14), 4288–95. 10.1016/j.bmc.2013.04.074. 23706267

[ref167] Abuo-Rahma G. E.-D. A. A.; Abdel-Aziz M.; Farag N. A.; Kaoud T. S. Novel 1-[4-(Aminosulfonyl)phenyl]-*1H*-1,2,4-triazole derivatives with remarkable selective COX-II inhibition: design, synthesis, molecular docking, anti-inflammatory and ulcerogenicity studies. Eur. J. Med. Chem. 2014, 83, 398–408. 10.1016/j.ejmech.2014.06.049. 24983538

[ref168] Abdel-Aziz M.; Beshr E. A.; Abdel-Rahman I. M.; Ozadali K.; Tan O. U.; Aly O. M. 1-(4-Methoxyphenyl)-5-(3,4,5-trimethoxyphenyl)-1*H*-1,2,4-triazole-3-carboxamides: Synthesis, molecular modeling, evaluation of their anti-inflammatory activity and ulcerogenicity. Eur. J. Med. Chem. 2014, 77, 155–65. 10.1016/j.ejmech.2014.03.001. 24631895

[ref169] Lee K.; Pham V. C.; Choi M. J.; Kim K. J.; Lee K.-T.; Han S.-G.; Yu Y. G.; Lee J. Y. Fragment-based discovery of novel and selective mPGES-1 inhibitors Part 1: Identification of sulfonamido-1,2,3-triazole-4,5-dicarboxylic acid. Bioorg. Med. Chem. Lett. 2013, 23 (1), 75–80. 10.1016/j.bmcl.2012.11.019. 23218602

[ref170] Jawabrah Al-Hourani B.; El-Barghouthi M. I.; Al-Awaida W.; McDonald R.; Fattash I. A.; El Soubani F.; Matalka K.; Wuest F. Biomolecular docking, synthesis, crystal structure, and bioassay studies of 1-[4-(2-chloroethoxy)phenyl]-5-[4-(methylsulfonyl)phenyl]-*1H*-tetrazole and 2-(4-(5-(4-(methylsulfonyl)phenyl)-*1H*-tetrazol-1-yl)phenoxy)ethyl nitrate. J. Mol. Struct. 2020, 1202, 127323 10.1016/j.molstruc.2019.127323.

[ref171] Singh P.; Bhardwaj A. *Mono*-, *di*-, and *tri*aryl substituted tetrahydropyrans as cyclooxygenase-II and tumor growth inhibitors. Synthesis and biological evaluation. J. Med. Chem. 2010, 53 (9), 3707–17. 10.1021/jm1001327. 20387815

[ref172] Tietz O.; Marshall A.; Wuest M.; Wang M.; Wuest F. Radiotracers for molecular imaging of cyclooxygenase-II (COX-II) enzyme. Curr. Med. Chem. 2013, 20 (35), 4350–69. 10.2174/09298673113206660260. 24059237

[ref173] Irannejad H.; Kebriaieezadeh A.; Zarghi A.; Montazer-Sadegh F.; Shafiee A.; Assadieskandar A.; Amini M. Synthesis, docking simulation, biological evaluations and 3D-QSAR study of 5-Aryl-6-(4-methylsulfonyl)-3-(metylthio)-1,2,4-triazine as selective cyclooxygenase-II inhibitors. Bioorg. Med. Chem. 2014, 22 (2), 865–873. 10.1016/j.bmc.2013.12.002. 24361187

[ref174] Dou J.; Shi L.; Hu A.; Dong M.; Xu J.; Liu A.; Jiang Y. Synthesis and evaluation of 2-(2-arylmorpholino)ethyl esters of Ibuprofen hydrochlorides as COX-II and serotonin reuptake inhibitors. Arch. Pharm. 2014, 347 (2), 89–95. 10.1002/ardp.201300279. 24243443

[ref175] Seebacher W.; Faist J.; Presser A.; Weis R.; Saf R.; Kaserer T.; Temml V.; Schuster D.; Ortmann S.; Otto N.; Bauer R. Synthesis of new 4-phenylpyrimidine-2(1*H*)-thiones and their potency to inhibit COX-I and COX-II. Eur. J. Med. Chem. 2015, 101, 552–559. 10.1016/j.ejmech.2015.07.003. 26197159

[ref176] Dadashpour S.; Tuylu Kucukkilinc T.; Unsal Tan O.; Ozadali K.; Irannejad H.; Emami S. Design, Synthesis and *in vitro* study of 5,6-diaryl-1,2,4-triazine-3-ylthioacetate derivatives as COX-2 and β-amyloid aggregation inhibitors. Arch. Pharm. 2015, 348 (3), 179–187. 10.1002/ardp.201400400. 25690564

[ref177] Tietz O.; Kaur J.; Bhardwaj A.; Wuest F. R. Pyrimidine-based fluorescent COX-II inhibitors: Synthesis and biological evaluation. Org. Biomol. Chem. 2016, 14 (30), 7250–7257. 10.1039/C6OB00493H. 27383140

[ref178] Jadhav S. B.; Fatema S.; Patil R. B.; Sangshetti J. N.; Farooqui M. Pyrido[1,2-*a*]pyrimidin-4-ones: ligand-based design, synthesis, and evaluation as an anti-inflammatory agent. J. Heterocycl. Chem. 2017, 54 (6), 3299–3313. 10.1002/jhet.2950.

[ref179] Rainsford K. D. Nsaid-mucosal injury: Roles of drug chemistry in pathogenesis. In Cell/tissue injury and cytoprotection/organoprotection in the gastrointestinal tract; Karger Publishers, 2012; Vol. 30, pp 79–86.

[ref180] Mishra A. P.; Bajpai A.; Chandra S. A comprehensive review on the screening models for the pharmacological assessment of antiulcer drugs. Current clinical pharmacology 2019, 14, 175–196. 10.2174/1574884714666190312143846. 30864527

[ref181] Beiranvand M. A review of the most common in vivo models of stomach ulcers and natural and synthetic anti-ulcer compounds: A comparative systematic study. Phytomedicine Plus 2022, 2, 100264 10.1016/j.phyplu.2022.100264.

[ref182] Ganguly A. K. A method for quantitative assessment of experimentally produced ulcers in the stomach of albino rats. Experientia 1969, 25, 1224–1224. 10.1007/BF01900290. 5357845

[ref183] Ibrahim T. H.; Loksha Y. M.; Elshihawy H. A.; Khodeer D. M.; Said M. M. Synthesis of some novel 2,6-disubstituted pyridazin-3(*2H*)-one derivatives as analgesic, anti-inflammatory, and non-ulcerogenic agents. Archiv der Pharmazie 2017, 350 (9), 1700093 10.1002/ardp.201700093. 28792072

[ref184] Singh P.; Kaur S.; Kumari P.; Kaur B.; Kaur M.; Singh G.; Bhatti R.; Bhatti M. Tailoring the Substitution Pattern on 1,3,5-Triazine for Targeting Cyclooxygenase-2: Discovery and Structure–Activity Relationship of Triazine–4-Aminophenylmorpholin-3-one Hybrids that Reverse Algesia and Inflammation in Swiss Albino Mice. J. Med. Chem. 2018, 61 (17), 7929–7941. 10.1021/acs.jmedchem.8b00922. 30095904

[ref185] Bakr R. B.; Ghoneim A. A.; Azouz A. A. Selective cyclooxygenase inhibition and ulcerogenic liability of some newly prepared anti-inflammatory agents having thiazolo[4,5-*d*]pyrimidine scaffold. Bioorg. Chem. 2019, 88, 102964 10.1016/j.bioorg.2019.102964. 31075742

[ref186] Muthuraman S.; Sinha S.; Vasavi C. S.; Waidha K. M.; Basu B.; Munussami P.; Balamurali M. M.; Doble M.; Saravana Kumar R. Design, synthesis and identification of novel coumaperine derivatives for inhibition of human 5-LOX: Antioxidant, pseudoperoxidase and docking studies. Bioorg. Med. Chem. 2019, 27 (4), 604–619. 10.1016/j.bmc.2018.12.043. 30638966

[ref187] Puratchikody A.; Umamaheswari A.; Irfan N.; Sinha S.; Manju S. L.; Ramanan M.; Ramamoorthy G.; Doble M. A novel class of tyrosine derivatives as dual 5-LOX and COX-II/mPGES1 inhibitors with PGE_2_ mediated anticancer properties. New J. Chem. 2019, 43 (2), 834–846. 10.1039/C8NJ04385J.

[ref188] Dravyakar R. B.; Khedekar B. P.; Khan T.; Sherje P. A.; Patel N. K.; Suvarna V. Design and development of novel 2-(morpholinyl)-*n*-substituted phenylquinazolin-4-amines as selective COX-II inhibitor. Anti-Inflamm. Anti-Allergy Agents Med. Chem. 2019, 18 (1), 4–25. 10.2174/1871523017666181022144053. PMC6446527 30345927

[ref189] Gouda A. M.; Abdelazeem A. H.; Omar H. A.; Abdalla A. N.; Abourehab M. A. S.; Ali H. I. Pyrrolizines: Design, synthesis, anticancer evaluation and investigation of the potential mechanism of action. Bioorg. Med. Chem. 2017, 25 (20), 5637–5651. 10.1016/j.bmc.2017.08.039. 28916158

[ref190] Tan C.-M.; Chen G. S.; Chen C.-S.; Chang P.-T.; Chern J.-W. Design, synthesis and biological evaluation of benzo[1.3.2]dithiazolium ylide 1,1-dioxide derivatives as potential dual cyclooxygenase-II/5-lipoxygenase inhibitors. Bioorg. Med. Chem. 2011, 19 (21), 6316–6328. 10.1016/j.bmc.2011.09.003. 21958737

[ref191] Naaz F.; Preeti Pallavi M. C.; Shafi S.; Mulakayala N.; Shahar Yar M.; Sampath Kumar H. M. 1,2,3-Triazole Tethered Indole-3-Glyoxamide Derivatives as Multiple Inhibitors of 5-LOX, COX-2 & Tubulin: Their Anti-Proliferative & Anti-Inflammatory Activity. Bioorg. Chem. 2018, 81, 1–20. 10.1016/j.bioorg.2018.07.029. 30081353

[ref192] Omar Y. M.; Abdu-Allah H. H. M.; Abdel-Moty S. G. Synthesis, biological evaluation and docking study of 1,3,4-thiadiazole-thiazolidinone hybrids as anti-inflammatory agents with dual inhibition of COX-II and 5-LOX. Bioorg. Chem. 2018, 80, 461–471. 10.1016/j.bioorg.2018.06.036. 29986191

[ref193] Abdelgawad M. A.; Bakr R. B.; Azouz A. A. Novel pyrimidine-pyridine hybrids: Synthesis, cyclooxygenase inhibition, anti-inflammatory activity and ulcerogenic liability. Bioorg. Chem. 2018, 77, 339–348. 10.1016/j.bioorg.2018.01.028. 29421710

[ref194] Banerjee A. G.; Das N.; Shengule S. A.; Sharma P. A.; Srivastava R. S.; Shrivastava S. K. Design, synthesis, evaluation and molecular modelling studies of some novel 5,6-diphenyl-1,2,4-triazin-3(2*H*)-ones bearing five-member heterocyclic moieties as potential COX-II inhibitors: A hybrid pharmacophore approach. Bioorg. Chem. 2016, 69, 102–120. 10.1016/j.bioorg.2016.10.003. 27750057

[ref195] El-Miligy M. M. M.; Hazzaa A. A.; El-Messmary H.; Nassra R. A.; El-Hawash S. A. M. New hybrid molecules combining benzothiophene or benzofuran with rhodanine as dual COX-I/II and 5-LOX inhibitors: Synthesis, biological evaluation and docking study. Bioorg. Chem. 2017, 72, 102–115. 10.1016/j.bioorg.2017.03.012. 28390993

[ref196] Tageldin G. N.; Fahmy S. M.; Ashour H. M.; Khalil M. A.; Nassra R. A.; Labouta I. M. Design, synthesis and evaluation of some pyrazolo[3,4-*d*]pyrimidine derivatives bearing thiazolidinone moiety as anti-inflammatory agents. Bioorg. Chem. 2018, 80, 164–173. 10.1016/j.bioorg.2018.06.013. 29929077

[ref197] Moussa G.; Alaaeddine R.; Alaeddine L. M.; Nassra R.; Belal A. S. F.; Ismail A.; El-Yazbi A. F.; Abdel-Ghany Y. S.; Hazzaa A. Novel click modifiable thioquinazolinones as anti-inflammatory agents: Design, synthesis, biological evaluation and docking study. Eur. J. Med. Chem. 2018, 144, 635–650. 10.1016/j.ejmech.2017.12.065. 29289887

[ref198] Banerjee A. G.; Das N.; Shengule S. A.; Srivastava R. S.; Shrivastava S. K. Synthesis, characterization, evaluation and molecular dynamics studies of 5,6-diphenyl-1,2,4-triazin-3(2*H*)-one derivatives bearing 5-substituted 1,3,4-oxadiazole as potential anti-inflammatory and analgesic agents. Eur. J. Med. Chem. 2015, 101, 81–95. 10.1016/j.ejmech.2015.06.020. 26117820

[ref199] Abd El Razik H. A.; Badr M. H.; Atta A. H.; Mouneir S. M.; Abu-Serie M. M. Benzodioxole-Pyrazole Hybrids as Anti-Inflammatory and Analgesic Agents with COX-I,II/5-LOX Inhibition and Antioxidant Potential. Arch. Pharm. 2017, 350 (5), 1700026 10.1002/ardp.201700026. 28418202

[ref200] Abdelazeem A. H.; Salama S. A.; Maghrabi I. A. Design, Synthesis, and Anti-Inflammatory Evaluation of Novel Diphenylthiazole-Thiazolidinone Hybrids. Arch. Pharm. 2015, 348 (7), 518–530. 10.1002/ardp.201500104. 25989149

[ref201] Boshra A. N.; Abdu-Allah H. H. M.; Mohammed A. F.; Hayallah A. M. Click chemistry synthesis, biological evaluation and docking study of some novel 2′-hydroxychalcone-triazole hybrids as potent anti-inflammatory agents. Bioorg. Chem. 2020, 95, 103505 10.1016/j.bioorg.2019.103505. 31901755

[ref202] Abdelall E. K. A. Synthesis and biological evaluations of novel isoxazoles and furoxan derivative as anti-inflammatory agents. Bioorg. Chem. 2020, 94, 103441 10.1016/j.bioorg.2019.103441. 31859011

[ref203] Abdellatif K.; Fadaly W.; Kamel G.; Elshaier Y.; El-Magd M. Design, Synthesis, Modeling Studies and Biological Evaluation of Thiazolidine Derivatives Containing Pyrazole Core as Potential Anti-Diabetic PPAR-γ Agonists and Anti-Inflammatory COX-2 Selective Inhibitors. Bioorg. Chem. 2019, 82, 82 10.1016/j.bioorg.2018.09.034. 30278282

[ref204] Zabiulla; Gulnaz A. R.; Mohammed Y. H. E.; Khanum S. A. Design, synthesis and molecular docking of benzophenone conjugated with oxadiazole sulphur bridge pyrazole pharmacophores as anti inflammatory and analgesic agents. Bioorg. Chem. 2019, 92, 103220 10.1016/j.bioorg.2019.103220. 31493708

[ref205] Shaveta; Singh A.; Kaur M.; Sharma S.; Bhatti R.; Singh P. Rational design, synthesis and evaluation of chromone-indole and chromone-pyrazole based conjugates: identification of a lead for anti-inflammatory drug. Eur. J. Med. Chem. 2014, 77, 185–92. 10.1016/j.ejmech.2014.03.003. 24631898

[ref206] Rathore A.; Rahman M. U.; Siddiqui A. A.; Ali A.; Shaharyar M. Design and synthesis of benzimidazole analogs endowed with oxadiazole as selective COX-II inhibitor. Arch. Pharm. 2014, 347 (12), 923–935. 10.1002/ardp.201400219. 25303727

[ref207] Yatam S.; Jadav S. S.; Gundla K. P.; Paidikondala K.; Ankireddy A. R.; Babu B. N.; Ahsan M. J.; Gundla R. 2-Mercapto benzthiazole coupled benzyl triazoles as new COX-II inhibitors: design, synthesis, biological testing and molecular modeling studies. ChemistrySelect 2019, 4 (37), 11081–11092. 10.1002/slct.201902972.

[ref208] Haider S.; Alam M. S.; Hamid H.; Shafi S.; Nargotra A.; Mahajan P.; Nazreen S.; Kalle A. M.; Kharbanda C.; Ali Y.; Alam A.; Panda A. K. Synthesis of novel 1,2,3-triazole based benzoxazolinones: Their TNF-α based molecular docking with *in vivo* anti-inflammatory, antinociceptive activities and ulcerogenic risk evaluation. Eur. J. Med. Chem. 2013, 70, 579–588. 10.1016/j.ejmech.2013.10.032. 24211633

[ref209] Hanna M. M. New pyrimido[5,4-e]pyrrolo[1,2-c]pyrimidines: Synthesis, 2D-QSAR, anti-inflammatory, analgesic and ulcerogenicity studies. Eur. J. Med. Chem. 2012, 55, 12–22. 10.1016/j.ejmech.2012.06.048. 22818041

[ref210] Kulkarni R.; Madar J.; Shastri S.; Shaikh F.; Naik N.; Chougale R.; Shastri L.; Joshi S.; Dixit S.; Sunagar V. Green Synthesis of Coumarin-Pyrazolone Hybrids: In Vitro Anticancer and Anti-Inflammatory Activities and Their Computational Study on COX-2 Enzyme. Chem. Data Collect. 2018, 17–18, 497–506. 10.1016/j.cdc.2018.11.004.

[ref211] Kaur S.; Kumari P.; Singh G.; Bhatti R.; Singh P. Design and synthesis of aza-/oxa heterocycle-based conjugates as novel anti-inflammatory agents targeting Cyclooxygenase-II. ACS Omega 2018, 3, 5825–5845. 10.1021/acsomega.8b00445. 30023927 PMC6044720

[ref212] Shen F.-Q.; Wang Z.-C.; Wu S.-Y.; Ren S.-Z.; Man R.-J.; Wang B.-Z.; Zhu H.-L. Synthesis of novel hybrids of pyrazole and coumarin as dual inhibitors of COX-II and 5-LOX. Bioorg. Med. Chem. Lett. 2017, 27 (16), 3653–3660. 10.1016/j.bmcl.2017.07.020. 28720504

[ref213] Hayashi S.; Sumi Y.; Ueno N.; Murase A.; Takada J. Discovery of a novel COX-II inhibitor as an orally potent anti-pyretic and anti-inflammatory drug: Design, synthesis, and structure-activity relationship. Biochem. Pharmacol. 2011, 82 (7), 755–768. 10.1016/j.bcp.2011.06.036. 21741371

[ref214] Galal S. A.; Khairat S. H. M.; Ragab F. A. F.; Abdelsamie A. S.; Ali M. M.; Soliman S. M.; Mortier J.; Wolber G.; El Diwani H. I. Design, synthesis and molecular docking study of novel quinoxalin-2(1*H*)-ones as anti-tumor active agents with inhibition of tyrosine kinase receptor and studying their cyclooxygenase-II activity. Eur. J. Med. Chem. 2014, 86, 122–132. 10.1016/j.ejmech.2014.08.048. 25147154

[ref215] Swiatek P.; Strzelecka M.; Urniaz R.; Gebczak K.; Gebarowski T.; Gasiorowski K.; Malinka W. Synthesis, COX-I/II inhibition activities and molecular docking study of isothiazolopyridine derivatives. Bioorg. Med. Chem. 2017, 25 (1), 316–326. 10.1016/j.bmc.2016.10.036. 27842798

[ref216] Shaikh M. M.; Patel A. P.; Patel S. P.; Chikhalia K. H. Synthesis, *in vitro* COX-I/COX-II inhibition testing and molecular docking study of novel 1,4-benzoxazine derivatives. New J. Chem. 2019, 43 (26), 10305–10317. 10.1039/C9NJ00684B.

[ref217] Medda F.; Sells E.; Chang H.-H.; Dietrich J.; Chappeta S.; Smith B.; Gokhale V.; Meuillet E. J.; Hulme C. Synthesis and biological activity of aminophthalazines and aminopyridazines as novel inhibitors of PGE_2_ production in cells. Bioorg. Med. Chem. Lett. 2013, 23 (2), 528–531. 10.1016/j.bmcl.2012.11.030. 23237838 PMC3534862

[ref218] El-Husseiny W. M.; El-Sayed M. A.; Abdel-Aziz N. I.; El-Azab A. S.; Asiri Y. A.; Abdel-Aziz A. A. Structural alterations based on naproxen scaffold: Synthesis, evaluation of antitumor activity and COX-II inhibition and molecular docking. Eur. J. Med. Chem. 2018, 158, 134–143. 10.1016/j.ejmech.2018.09.007. 30216848

[ref219] Santos R. L. S. R.; Bergamo A.; Sava G.; de Oliveira Silva D. Synthesis and characterization of a diruthenium(II,III)-ketoprofen compound and study of the *in vitro* effects on CRC cells in comparison to the Naproxen and Ibuprofen derivatives. Polyhedron 2012, 42 (1), 175–181. 10.1016/j.poly.2012.05.012.

[ref220] Obermoser V.; Baecker D.; Schuster C.; Braun V.; Kircher B.; Gust R. Chlorinated cobalt alkyne complexes derived from acetylsalicylic acid as new specific antitumor agents. Dalton Trans. 2018, 47 (12), 4341–4351. 10.1039/C7DT04790H. 29492489

[ref221] Baecker D.; Obermoser V.; Kirchner E. A.; Hupfauf A.; Kircher B.; Gust R. Fluorination as tool to improve bioanalytical sensitivity and COX-II-selective antitumor activity of cobalt alkyne complexes. Dalton Trans. 2019, 48 (42), 15856–15868. 10.1039/C9DT03330K. 31617517

[ref222] Ren S.-Z.; Wang Z.-C.; Zhu D.; Zhu X.-H.; Shen F.-Q.; Wu S.-Y.; Chen J.-J.; Xu C.; Zhu H.-L. Design, synthesis and biological evaluation of novel ferrocene-pyrazole derivatives containing nitric oxide donors as COX-II inhibitors for cancer therapy. Eur. J. Med. Chem. 2018, 157, 909–924. 10.1016/j.ejmech.2018.08.048. 30149323

[ref223] Tabrizi L.; Olasunkanmi L. O.; Fadare O. A. Experimental and theoretical investigations of cyclometalated ruthenium(ii) complex containing CCC-pincer and anti-inflammatory drugs as ligands: synthesis, characterization, inhibition of cyclooxygenase and *in vitro* cytotoxicity activities in various cancer cell lines. Dalton Trans. 2019, 48 (2), 728–740. 10.1039/C8DT03266A. 30560261

[ref224] Gautam R.; Jachak S. M. Recent developments in anti-inflammatory natural products. Med. Res. Rev. 2009, 29 (5), 767–820. 10.1002/med.20156. 19378317

[ref225] Gosslau A.; Li S.; Ho C.-T.; Chen K. Y.; Rawson N. E. The importance of natural product characterization in studies of their anti-inflammatory activity. Mol. Nutr. Food Res. 2011, 55 (1), 74–82. 10.1002/mnfr.201000455. 21207514

[ref226] Gautam R.; Srivastava A.; Jachak S. M.; Saklani A. Anti-inflammatory, cyclooxygenase (COX)-II, COX-I inhibitory and antioxidant effects of Dysophylla stellata Benth. Fitoterapia 2010, 81 (1), 45–49. 10.1016/j.fitote.2009.07.004. 19632309

[ref227] Katsukawa M.; Nakata R.; Takizawa Y.; Hori K.; Takahashi S.; Inoue H. Citral, a component of lemongrass oil, activates PPARα and γ and suppresses COX-II expression. Biochim Biophys Acta Mol. Cell Biol. Lipids 2010, 1801 (11), 1214–1220. 10.1016/j.bbalip.2010.07.004. 20656057

[ref228] Huang G.-J.; Deng J.-S.; Liao J.-C.; Hou W.-C.; Wang S.-Y.; Sung P.-J.; Kuo Y.-H. Inducible nitric oxide synthase and cyclooxygenase-II participate in anti-inflammatory activity of imperatorin from glehnia littoralis. J. Agric. Food Chem. 2012, 60 (7), 1673–1681. 10.1021/jf204297e. 22188242

[ref229] Waller C. P.; Thumser A. E.; Langat M. K.; Crouch N. R.; Mulholland D. A. COX-II inhibitory activity of homoisoflavanones and xanthones from the bulbs of the southern african ledebouria socialis and ledebouria ovatifolia (Hyacinthaceae: Hyacinthoideae). Phytochemistry 2013, 95, 284–290. 10.1016/j.phytochem.2013.06.024. 23859260

[ref230] Romero J. C.; Martínez-Vázquez A.; Herrera M. P.; Martinez-Mayorga K.; Parra-Delgado H.; Pérez-Flores F. J.; Martínez-Vázquez M. Synthesis, anti-inflammatory activity and modeling studies of cycloartane-type terpenes derivatives isolated from Parthenium argentatum. Bioorg. Med. Chem. 2014, 22 (24), 6893–6898. 10.1016/j.bmc.2014.10.028. 25456078

[ref231] Ma Q.-G.; Wei R.-R.; Sang Z.-P. Biphenyl Derivatives from the Aerial Parts of *Oenanthe javanica* and Their COX-2 Inhibitory Activities. C&B 2019, 16 ( (1), ), e1800480. 10.1002/cbdv.201800480 30378266

[ref232] Santos M. F. C.; Alcântara B. G. V.; Feliciano C.; Silva A. F.; Maiolini T. C. S.; Neto A. K.; Murgu M.; de Paula D. A. C.; Soares M. G. New bicyclic [3.2.1] octane neolignans derivatives from Aniba firmula with potent *in vivo* anti-inflammatory activity on account of dual inhibition of PGE_2_ production and cell recruitment. Phytochem. Lett. 2019, 30, 31–37. 10.1016/j.phytol.2019.01.014.

[ref233] Kang S.-Y.; Shin J.-S.; Kim S.-Y.; Noh Y. S.; Lee S.-J.; Hwang H.; Deyou T.; Jang Y. P.; Lee K.-T. Caffeoyloxy-5,6-dihydro-4-methyl-(2*H*)-pyran-2-one isolated from the leaves of Olinia usambarensis attenuates LPS-induced inflammatory mediators by inactivating AP-1 and NF-κB. Chem. Biol. Interact. 2019, 309 (108718), 492–504. 10.1016/j.cbi.2019.06.031. 31211952

[ref234] Deb P. K.; Mailabaram R. P.; Al-Jaidi B.; Saadh M. Molecular basis of binding interactions of NSAIDs and computer-aided drug design approaches in the pursuit of the development of cyclooxygenase-II (COX-II) selective inhibitors. Nonsteroidal Anti Inflamm Drugs 2017, 23 (6), 101–121. 10.5772/intechopen.68318.

[ref235] Narsinghani T.; Chaturvedi S. C. QSAR analysis of meclofenamic acid analogues as selective COX-II inhibitors. Bioorg. Med. Chem. Lett. 2006, 16 (2), 461–468. 10.1016/j.bmcl.2005.07.067. 16290292

[ref236] Soltani S.; Abolhasani H.; Zarghi A.; Jouyban A. QSAR analysis of diaryl COX-II inhibitors: comparison of feature selection and train-test data selection methods. European journal of medicinal chemistry 2010, 45 (7), 2753–2760. 10.1016/j.ejmech.2010.02.055. 20332057

[ref237] Prasanna S.; Manivannan E.; Chaturvedi S. C. QSAR analysis of 2,3-diaryl benzopyrans/pyrans as selective COX-II inhibitors based on semiempirical am1 calculations. QSAR & Combinatorial Science 2004, 23 (8), 621–628. 10.1002/qsar.200430887.

[ref238] Arrault A.; Monge A.; Marot C.; Morin-Allory L. Computer-aided design of original COX-II-inhibitors: Docking protocols and virtual screening under pharmacophoric constraints, 10th Electronic Comp. Chem. Conference (ECCC10), 2005; pp 9–10.

[ref239] Uddin M. J.; Rao P. P.; Knaus E. E. Design and synthesis of novel Celecoxib analogues as selective cyclooxygenase-II (COX-II) inhibitors: replacement of the sulfonamide pharmacophore by a sulfonylazide bioisostere. Bioorganic & medicinal chemistry 2003, 11 (23), 5273–5280. 10.1016/j.bmc.2003.07.005. 14604691

[ref240] Razzaghi-Asl N.; Mirzayi S.; Mahnam K.; Sepehri S. Identification of COX-II inhibitors *via* structure-based virtual screening and molecular dynamics simulation. Journal of Molecular Graphics and Modelling 2018, 83, 138–152. 10.1016/j.jmgm.2018.05.010. 29936228

[ref241] Hollingsworth S. A.; Dror R. O. Molecular Dynamics Simulation for All. Neuron 2018, 99 (6), 1129–1143. 10.1016/j.neuron.2018.08.011. 30236283 PMC6209097

[ref242] Bhutani P.; Joshi G.; Raja N.; Bachhav N.; Rajanna P. K.; Bhutani H.; Paul A. T.; Kumar R. U.S. FDA Approved Drugs from 2015-June 2020: A Perspective. J. Med. Chem. 2021, 64 (5), 2339–2381. 10.1021/acs.jmedchem.0c01786. 33617716

[ref243] Naveja J. J.; Medina-Franco J. L. Finding constellations in chemical space through core analysis. Frontiers in chemistry 2019, 7, 510 10.3389/fchem.2019.00510. 31380353 PMC6646408

[ref244] Naveja J. J.; Medina-Franco J. L. Finding constellations in chemical space through core analysis. Frontiers in Chemistry 2019, 7, 510 10.3389/fchem.2019.00510. 31380353 PMC6646408

[ref245] Benet L. Z.; Hosey C. M.; Ursu O.; Oprea T. I. BDDCS, the Rule of 5 and drugability. Advanced drug delivery reviews 2016, 101, 89–98. 10.1016/j.addr.2016.05.007. 27182629 PMC4910824

[ref246] Zhang M.-Q.; Wilkinson B. Drug discovery beyond the ‘rule-of-five’. Curr. Opin. Biotechnol. 2007, 18 (6), 478–488. 10.1016/j.copbio.2007.10.005. 18035532

[ref247] Veber D. F.; Johnson S. R.; Cheng H.-Y.; Smith B. R.; Ward K. W.; Kopple K. D. Molecular properties that influence the oral bioavailability of drug candidates. J. Med. Chem. 2002, 45 (12), 2615–2623. 10.1021/jm020017n. 12036371

[ref248] Daina A.; Michielin O.; Zoete V. iLOGP: A simple, robust, and efficient description of n-octanol/water partition coefficient for drug design using the GB/SA approach. J. Chem. Inf. Model. 2014, 54 (12), 3284–3301. 10.1021/ci500467k. 25382374

[ref249] Gonzalez-Medina M.; Medina-Franco J. L. Platform for unified molecular analysis: PUMA. J. Chem. Inf Model 2017, 57 (8), 1735–1740. 10.1021/acs.jcim.7b00253. 28737911

