# Supplementary material for: Design and Development of COX-II Inhibitors: Current Scenario and Future Perspective
Source: ACS Omega. 2023 May 9;8(20):17446–98. doi: 10.1021/acsomega.3c00692 (PMC10210234; doi:10.1021/acsomega.3c00692)
Supplement: Supplementary file 1 — ao3c00692_si_001.pdf [file ao3c00692_si_001.pdf]

# Design and development of COX-II inhibitors: Current scenario and future prospective

Sandhya Chahal [a,#], Payal Rani [a,#], Kiran [a], Jayant Sindhu [a,*], Gaurav Joshi [b,c], Aravindhan Ganesan [d], Subha Kalyaanamoorthy [e], Mayank [f], Parvin Kumar [g], Rajvir Singh [a] & Arvind Negi [h,*]

[a] *Department of Chemistry, COBS&H, CCS Haryana Agricultural University, Hisar 125004, India*

[b] *Department of Pharmaceutical Sciences, Hemvati Nandan Bahuguna Garhwal (A Central) University, Chauras Campus, Tehri Garhwal - 249161 Uttarakhand, India*

[c] *Adjunct Faculty at Department of Biotechnology, Graphic Era (Deemed to be) University, 566/6, Bell Road, Clement Town, Dehradun - 248002, Uttarakhand, India*

[d] *ArGan'sLab, School of Pharmacy, University of Waterloo, Waterloo, Ontario N2G 1C5, Canada*

[e] *Department of Chemistry, University of Waterloo, Waterloo, Ontario N2L 3G1, Canada*

[f] University college of pharmacy, Guru Kashi University, Talwandi Sabo, Punjab 151302, India

[g] *Department of Chemistry, Kurukshetra University, Kurukshetra, India-136119*

[h] *Department of Bioproducts and Biosystems, School of Chemical Engineering, Aalto University, Espoo, 02150, Finland*

# Authors shared equal contribution, * Corresponding author

**Table S1.** Physiochemical parameters of COX inhibitors

| RANK | CATEGORY | ID | MW | TopoPSA | nRotB | nHBDon | nHBAcc | ALogP |
|---|---|---|---|---|---|---|---|---|
| 1. | 1,2,4-Trisubstitued pyrazole/ pyrazoline | **PYZ18** | 361.0861 | 73.71 | 4 | 1 | 3 | 3.5995 |
| 2. | 1,2,4-Trisubstitued pyrazole/ pyrazoline | **PYZ19** | 375.0766 | 68.86 | 5 | 0 | 5 | 1.0251 |
| 3. | 1,2,4-Trisubstitued pyrazole/ pyrazoline | **PYZ20** | 435.1253 | 102.6 | 4 | 1 | 7 | 0.6135 |
| 4. | 1,2,4-Trisubstitued pyrazole/ pyrazoline | **PYZ21** | 498.1537 | 96.61 | 5 | 1 | 7 | 1.1592 |
| 5. | 1,3,4 and 1,3,4,5-Substituted | **PYZ41** | 490.1448 | 41.9 | 8 | 0 | 4 | 4.1155 |

| | | | | | | | | |
|---|---|---|---|---|---|---|---|---|
| | pyrazole | | | | | | | |
| 6. | 1,3,4 and 1,3,4,5-Substituted pyrazole | **PYZ42** | 342.1481 | 47.94 | 5 | 1 | 5 | 2.0163 |
| 7. | 1,3,4 and 1,3,4,5-Substituted pyrazole | **PYZ43** | 417.1244 | 46.42 | 6 | 0 | 5 | 2.1171 |
| 8. | 1,3,4-Substituted pyrazole | **ODZ1** | 490.0189 | 80.98 | 7 | 2 | 5 | 3.8296 |
| 9. | 1,3,4-Substituted pyrazole | **ODZ2** | 345.0419 | 119.61 | 4 | 0 | 5 | 1.961 |
| 10. | 1,3,4-Substituted pyrazole | **ODZ3** | 356.0831 | 94.78 | 7 | 0 | 6 | 2.1636 |
| 11. | 1,3,4-Substituted pyrazole | **ODZ4** | 375.0444 | 101.07 | 6 | 1 | 4 | 3.0728 |
| 12. | 1,3,4-Substituted pyrazole | **ODZ5** | 401.1012 | 120.58 | 6 | 3 | 8 | 2.0232 |
| 13. | 1,3,4-Substituted pyrazole | **ODZ6** | 281.0913 | 87.44 | 5 | 2 | 7 | 0.5788 |
| 14. | 1,3,4,5-Substituted pyrazole | **PYZ24** | 395.1052 | 119.97 | 4 | 2 | 8 | 0.5059 |
| 15. | 1,3,4,5-Substituted pyrazole | **PYZ25** | 319.0627 | 110.44 | 2 | 1 | 7 | -0.2918 |
| 16. | 1,3,4,5-Substituted pyrazole | **PYZ26** | 416.0903 | 32.67 | 4 | 0 | 3 | 3.1818 |
| 17. | 1,3,4,5-Substituted pyrazole | **PYZ29** | 461.1488 | 104.12 | 5 | 0 | 5 | 3.9784 |
| 18. | 1,3,4,5-Substituted pyrazole | **PYZ27** | 459.1325 | 35.91 | 5 | 0 | 4 | 3.7965 |
| 19. | 1,3,5-Susbtituted pyrazole | **PYZ36** | 411.1947 | 46.42 | 8 | 0 | 5 | 3.0893 |
| 20. | 1,3,5-Susbtituted pyrazole | **PYZ37** | 338.1201 | 88.82 | 6 | 2 | 5 | 1.321 |
| 21. | 1,3,5-Susbtituted pyrazole | **PYZ40** | 456.1838 | 41.9 | 8 | 0 | 4 | 3.7468 |
| 22. | 2,3-Substituted pyrazole | **PYZ44** | 432.1485 | 71.36 | 6 | 1 | 6 | 1.3135 |
| 23. | 2,3-Substituted pyrazole | **PYZ45** | 492.0685 | 71.36 | 6 | 1 | 6 | 1.8564 |

| | | | | | | | | |
|---|---|---|---|---|---|---|---|---|
| 24. | 2,3-Substituted pyrazole | **PYZ46** | 444.1685 | 80.59 | 7 | 1 | 7 | 0.9051 |
| 25. | 3,4 and 3,4,5- Susbtituted isoxazole | **IXZ1** | 249.046 | 73.19 | 4 | 0 | 2 | 0.9157 |
| 26. | 3,4 and 3,4,5- Susbtituted isoxazole | **IXZ2** | 279.0565 | 82.42 | 5 | 0 | 3 | 0.4171 |
| 27. | Cinnoline linked pyrazole | **PYZ47** | 354.0684 | 57.39 | 2 | 0 | 5 | 1.6453 |
| 28. | Cinnoline linked pyrazole | **PYZ48** | 356.0476 | 66.62 | 3 | 0 | 6 | 0.6539 |
| 29. | Coumarin derivatives | **CMN1** | 178.0266 | 66.76 | 0 | 2 | 4 | -0.0952 |
| 30. | Coumarin derivatives | **CMN4** | 162.0317 | 46.53 | 0 | 1 | 3 | 0.4679 |
| 31. | Coumarin derivatives | **CMN5** | 366.0674 | 102.29 | 5 | 1 | 6 | 1.8799 |
| 32. | Coumarin derivatives | **CMN9** | 352.1311 | 53.99 | 3 | 0 | 5 | 1.1946 |
| 33. | Coumarin derivatives | **CMN 6** | 275.0728 | 94.81 | 4 | 2 | 5 | 1.2201 |
| 34. | Coumarin derivatives | **CMN 7** | 373.0732 | 133.93 | 4 | 1 | 8 | 1.0955 |
| 35. | Coumarin derivatives | **Cmn10** | 349.1552 | 41.46 | 1 | 1 | 5 | 1.2193 |
| 36. | Coumarin derivatives | **Cmn11** | 374.163 | 24.83 | 2 | 0 | 5 | 2.0313 |
| 37. | COX/LOX inhibitors | **Licofelone** | 379.1339 | 40.54 | 4 | 1 | 3 | 3.4903 |
| 38. | COX/LOX inhibitors | **Lonapalene** | 338.0557 | 71.06 | 6 | 0 | 6 | 0.2633 |
| 39. | COX/LOX inhibitors | **Penidone** | 162.0793 | 32.34 | 1 | 1 | 3 | 0.3814 |
| 40. | Hybridised molecules | **HYB1** | 449.1852 | 86.16 | 8 | 2 | 7 | 0.6944 |
| 41. | Hybridised molecules | **HYB2** | 449.1852 | 86.16 | 8 | 2 | 7 | 0.6944 |
| 42. | Hybridised molecules | **HYB3** | 449.1852 | 86.16 | 8 | 2 | 7 | 0.6944 |
| 43. | Hybridised molecules | **HYB4** | 385.0845 | 135.39 | 4 | 3 | 9 | -0.6958 |
| 44. | Hybridised molecules | **HYB11** | 489.0662 | 123.23 | 6 | 1 | 8 | 2.5375 |

| | | | | | | | | |
|---|---|---|---|---|---|---|---|---|
| 45. | Hybridised molecules | **HYB6** | 467.1342 | 134.15 | 7 | 1 | 8 | 2.5188 |
| 46. | Hybridised molecules | **HYB7** | 468.1368 | 116.31 | 7 | 1 | 8 | 2.3651 |
| 47. | Hybridised molecules | **HYB9** | 434.062 | 138 | 4 | 1 | 8 | 2.6287 |
| 48. | Hybridised molecules | **HYB12** | 439.1281 | 119.44 | 5 | 2 | 9 | 1.4234 |
| 49. | Hybridised molecules | **HYB13** | 340.0266 | 123.98 | 4 | 0 | 3 | 3.081 |
| 50. | Hybridised molecules | **HYB15** | 440.0766 | 116.78 | 4 | 1 | 5 | 3.3651 |
| 51. | Hybridised molecules | **HYB16** | 489.097 | 104.42 | 4 | 1 | 4 | 4.7694 |
| 52. | Hybridised molecules | **HYB18** | 480.1434 | 111.87 | 7 | 1 | 9 | 0.2879 |
| 53. | Hybridised molecules | **HYB19** | 309.1113 | 71.33 | 4 | 0 | 4 | 0.688 |
| 54. | Hybridised molecules | **HYB20** | 455.061 | 138.82 | 5 | 1 | 8 | 0.9266 |
| 55. | Hybridised molecules | **HYB21** | 455.061 | 138.82 | 5 | 1 | 8 | 0.9266 |
| 56. | Hybridised molecules | **HYB23** | 289.0739 | 55.4 | 1 | 1 | 4 | 0.4927 |
| 57. | Hybridised molecules | **HYB24** | 366.9844 | 55.4 | 1 | 1 | 4 | 0.9454 |
| 58. | Hybridised molecules | **HYB25** | 352.1106 | 99.57 | 6 | 0 | 7 | -0.4342 |
| 59. | Hybridised molecules | **HYB27** | 372.027 | 90.92 | 5 | 0 | 4 | 3.2533 |
| 60. | Hybridised molecules | **HYB28** | 324.1023 | 57.5 | 3 | 0 | 6 | 1.1597 |
| 61. | Hybridised molecules | **HYB30** | 466.1753 | 97.68 | 4 | 1 | 9 | 0.2167 |
| 62. | Hybridised molecules | **HYB31** | 304.0848 | 67.76 | 1 | 1 | 5 | 0.9215 |
| 63. | Hybridised molecules | **HYB32** | 344.1161 | 58.97 | 2 | 0 | 5 | 1.7801 |
| 64. | Hybridised molecules | **HYB35** | 376.1035 | 78.76 | 6 | 2 | 5 | 0.3582 |
| 65. | Hybridised molecules | **HYB36** | 429.1913 | 93.92 | 5 | 2 | 9 | 0.4253 |

| | | | | | | | | |
|---|---|---|---|---|---|---|---|---|
| 66. | Hybridised molecules | **HYB37** | 442.223 | 87.93 | 5 | 2 | 9 | 0.6949 |
| 67. | Hybridised molecules | **HYB38** | 387.1808 | 84.69 | 5 | 2 | 8 | 0.7376 |
| 68. | Isatin derivatives | **IST1** | 440.071 | 113.24 | 5 | 2 | 7 | 0.7616 |
| 69. | Isatin derivatives | **IST2** | 480.0103 | 139.54 | 7 | 2 | 9 | -0.314 |
| 70. | Isatin derivatives | **IST3** | 282.1368 | 40.62 | 2 | 0 | 4 | 0.0399 |
| 71. | Miscellaneous five membered | **MIS1** | 394.0655 | 93.79 | 5 | 1 | 5 | 0.6414 |
| 72. | Miscellaneous five membered | **MIS2** | 418.1794 | 68.49 | 5 | 1 | 5 | 1.9129 |
| 73. | Miscellaneous five membered | **MIS3** | 366.0708 | 97.69 | 3 | 1 | 3 | 2.1038 |
| 74. | Miscellaneous five membered | **MIS4** | 386.0395 | 97.69 | 3 | 1 | 3 | 1.7554 |
| 75. | Miscellaneous molecules | **MISF1** | 401.0965 | 69.16 | 5 | 0 | 5 | 1.5133 |
| 76. | Miscellaneous molecules | **MISF2** | 286.1106 | 33.62 | 2 | 1 | 3 | 0.7718 |
| 77. | Miscellaneous molecules | **MISF3** | 299.131 | 12.47 | 2 | 0 | 2 | 2.461 |
| 78. | Miscellaneous molecules | **MISF4** | 427.2147 | 46.61 | 8 | 0 | 4 | 3.4046 |
| 79. | Miscellaneous molecules | **NPX1** | 346.1317 | 63.41 | 4 | 1 | 5 | 2.2949 |
| 80. | Miscellaneous molecules | **NPX2** | 404.1307 | 101.54 | 5 | 2 | 6 | 2.465 |
| 81. | Molecules derived from natural products | **NP (1)** | 342.074 | 113.29 | 3 | 3 | 7 | -0.3826 |
| 82. | Molecules derived from natural products | **NP (2)** | 456.3603 | 46.53 | 1 | 1 | 3 | 1.3908 |
| 83. | Molecules derived from natural products | **NP (3)** | 426.277 | 71.44 | 3 | 1 | 4 | 1.7298 |

| | | | | | | | | |
|---|---|---|---|---|---|---|---|---|
| 84. | Molecules derived from natural products | **NP (4)** | 336.1725 | 49.69 | 3 | 2 | 3 | 3.2366 |
| 85. | Molecules derived from natural products | **NP (5)** | 448.2097 | 100.52 | 9 | 1 | 8 | -0.9413 |
| 86. | Molecules derived from natural products | **NP (6)** | 474.2254 | 100.52 | 10 | 1 | 8 | 0.4165 |
| 87. | Molecules derived from natural products | **NP (7)** | 290.079 | 93.06 | 4 | 2 | 6 | 0.9951 |
| 88. | Molecules derived from natural products | **NP (8)** | 444.2876 | 75.99 | 0 | 2 | 5 | -0.7881 |
| 89. | Non Heterocyclic compounds | **NHC1** | 267.0662 | 58.89 | 5 | 1 | 2 | 1.3422 |
| 90. | Non Heterocyclic compounds | **NHC2** | 232.1212 | 64.68 | 5 | 1 | 2 | 0.5785 |
| 91. | Non Heterocyclic compounds | **NHC3** | 362.0977 | 59.59 | 5 | 0 | 3 | 2.8621 |
| 92. | Non Heterocyclic compounds | **NHC4** | 316.2038 | 46.53 | 9 | 1 | 3 | 2.7033 |
| 93. | Non Heterocyclic compounds | **NHC5** | 232.1099 | 46.53 | 5 | 1 | 3 | 2.296 |
| 94. | Non Heterocyclic compounds | **NHC8** | 226.0994 | 37.3 | 3 | 1 | 2 | 2.4614 |
| 95. | Pyrazole linked acetamide | **PYZ31** | 339.0193 | 137.03 | 5 | 2 | 8 | -0.2238 |
| 96. | Pyrazole linked benzoxazolone | **PYZ3** | 389.0987 | 54.37 | 4 | 0 | 6 | 1.7105 |
| 97. | Pyrazole linked benzoxazolone | **PYZ4** | 393.0492 | 45.14 | 3 | 0 | 5 | 2.5778 |
| 98. | Pyrazole linked chromenone | **PYZ30** | 454.174 | 88.05 | 7 | 0 | 9 | -1.1577 |
| 99. | Pyrazole linked pyrazoline | **PYZ5** | 302.1531 | 39.99 | 3 | 1 | 4 | 1.7994 |

| | | | | | | | | |
|---|---|---|---|---|---|---|---|---|
| 100. | Pyrazole linked pyrazoline | **PYZ6** | 366.048 | 39.99 | 3 | 1 | 4 | 1.8057 |
| 101. | Pyrazole linked thiadiazole and thiazole | **PYZ38** | 435.1365 | 111.9 | 7 | 1 | 8 | 1.3507 |
| 102. | Pyrazole linked thiadiazole and thiazole | **PYZ39** | 421.1209 | 122.9 | 6 | 2 | 8 | 1.2862 |
| 103. | Pyrazole linked thiadiazole and thiazole | **PYZ35** | 375.0766 | 68.86 | 5 | 0 | 5 | 1.0251 |
| 104. | Pyrazole linked thiazoline | **PYZ1** | 350.1201 | 96.02 | 4 | 2 | 5 | 1.3453 |
| 105. | Pyrazole linked thiazoline | **PYZ2** | 364.1358 | 96.02 | 4 | 2 | 5 | 1.7917 |
| 106. | Pyrazole linked thiourea | **PYZ10** | 331.1467 | 109.64 | 6 | 4 | 6 | -1.7858 |
| 107. | Pyrazole/pyrazoline linked hydrazone, benzoimidazole and benzothiazole | **PYZ8** | 360.1335 | 98.52 | 4 | 2 | 8 | -0.5759 |
| 108. | Pyrazole/pyrazoline linked hydrazone, benzoimidazole and benzothiazole | **PYZ9** | 411.1154 | 94.72 | 4 | 1 | 6 | 1.5962 |
| 109. | Pyrazole/pyrazoline linked hydrazone, benzoimidazole and benzothiazole | **PYZ11** | 271.1433 | 72.58 | 5 | 2 | 6 | -0.4849 |
| 110. | Selective COX-II inhibitors | **Ibuprofen** | 206.1307 | 37.3 | 4 | 1 | 2 | 1.9613 |
| 111. | Selective COX-II inhibitors | **Indomethacin** | 357.0768 | 66.84 | 5 | 1 | 5 | 1.264 |

| | | | | | | | | |
|---|---|---|---|---|---|---|---|---|
| 112. | Selective COX-II inhibitors | **Diclofenac** | 295.0167 | 49.33 | 4 | 2 | 3 | 1.8296 |
| 113. | Selective COX-II inhibitors | **Acetylsalicylic acid** | 180.0423 | 63.6 | 3 | 1 | 4 | 0.44 |
| 114. | Selective COX-II inhibitors | **Celecoxib** | 381.0759 | 84.14 | 4 | 1 | 5 | 2.1475 |
| 115. | Selective COX-II inhibitors | **Rofecoxib** | 314.0613 | 68.82 | 3 | 0 | 4 | 1.643 |
| 116. | Selective COX-II inhibitors | **Etoricoxib** | 358.0543 | 67.24 | 3 | 0 | 4 | 1.7643 |
| 117. | Selective COX-II inhibitors | **Valdecoxib** | 313.0773 | 64.11 | 3 | 0 | 2 | 1.6511 |
| 118. | Six membered exclude pyridine | **NHCXP1** | 438.142 | 54.76 | 6 | 1 | 2 | 4.1682 |
| 119. | Six membered exclude pyridine | **NHCXP2** | 425.0821 | 79.27 | 6 | 1 | 5 | 1.8697 |
| 120. | Six membered exclude pyridine | **NHCXP3** | 392.0168 | 130.92 | 4 | 1 | 6 | 1.7099 |
| 121. | Six membered exclude pyridine | **NHCXP5** | 249.0572 | 99.29 | 2 | 2 | 2 | 2.1026 |
| 122. | Six membered exclude pyridine | **NHCXP9** | 244.1212 | 41.9 | 4 | 0 | 4 | 0.8646 |
| 123. | Six membered exclude pyridine | **NHCXP10** | 374.1258 | 81.89 | 4 | 1 | 8 | -1.8268 |
| 124. | Six membered exclude pyridine | **NHCXP11** | 430.0712 | 90.68 | 5 | 2 | 8 | 0.0513 |
| 125. | Six membered exclude pyridine | **NHCXP13** | 289.1314 | 81 | 4 | 3 | 5 | -1.1282 |
| 126. | Six membered exclude pyridine | **NHCXP14** | 273.1365 | 60.77 | 4 | 2 | 4 | -0.5651 |
| 127. | Six membered exclude pyridine | **NHCXP15** | 385.0499 | 125.8 | 8 | 2 | 8 | -0.9818 |
| 128. | Six membered exclude pyridine | **NHCXP17** | 324.1586 | 69.12 | 3 | 3 | 6 | 0.2891 |
| 129. | Substitued pyrole and pyrrolidine | **PRLD1** | 476.1054 | 124.43 | 10 | 0 | 5 | 2.263 |
| 130. | Substitued pyrole and pyrrolidine | **PRLD2** | 431.1203 | 92.29 | 8 | 1 | 6 | 1.1102 |
| 131. | Substitued pyrole and pyrrolidine | **PRLD3** | 395.0143 | 117.95 | 4 | 2 | 7 | -0.3493 |

| | | | | | | | | |
|---|---|---|---|---|---|---|---|---|
| 132. | Substitued pyrole and pyrrolidine | **PRLD4** | 373.0732 | 135.35 | 5 | 1 | 8 | -1.8767 |
| 133. | Substitued pyrole and pyrrolidine | **PRLD5** | 386.0936 | 122.99 | 5 | 1 | 7 | 0.1303 |
| 134. | Substitued pyrole and pyrrolidine | **PRLD6** | 376.0285 | 105.92 | 3 | 1 | 6 | 0.8216 |
| 135. | Substitued pyrole and pyrrolidine | **PRLD8** | 431.1389 | 71.06 | 8 | 0 | 3 | 2.169 |
| 136. | Substituted Benzoxazoles derivatives | **BXZ1** | 321.0557 | 41.82 | 2 | 1 | 3 | 2.6025 |
| 137. | Substituted Benzoxazoles derivatives | **BXZ2** | 335.0713 | 30.82 | 3 | 0 | 3 | 2.667 |
| 138. | Substituted Benzoxazoles derivatives | **BXZ3** | 408.0877 | 69.15 | 6 | 1 | 6 | 1.2824 |
| 139. | Substituted Benzoxazoles derivatives | **BXZ4** | 475.1088 | 54.26 | 4 | 0 | 5 | 3.5431 |
| 140. | Substituted Benzoxazoles derivatives | **BXZ5** | 485.1739 | 63.49 | 5 | 0 | 6 | 3.1222 |
| 141. | Substituted Benzoxazoles derivatives | **BXZ6** | 408.0877 | 69.15 | 6 | 1 | 6 | 1.2824 |
| 142. | Substituted Benzoxazoles derivatives | **BXZ7** | 388.1423 | 69.15 | 6 | 1 | 6 | 1.3601 |
| 143. | Substituted Benzoxazoles derivatives | **BXZ8** | 374.1267 | 69.15 | 6 | 1 | 6 | 0.9137 |
| 144. | Substituted Benzoxazoles | **BXZ9** | 340.0266 | 123.98 | 4 | 0 | 3 | 3.081 |

| | | | | | | | | |
|---|---|---|---|---|---|---|---|---|
| | derivatives | | | | | | | |
| 145. | Substituted indole derivatives | **IND4** | 461.1306 | 53.6 | 7 | 2 | 5 | 1.2631 |
| 146. | Substituted indole derivatives | **IND7** | 376.1035 | 78.76 | 6 | 2 | 5 | 0.3582 |
| 147. | Substituted indole derivatives | **IND8** | 412.1057 | 80.57 | 5 | 2 | 4 | 1.7953 |
| 148. | Substituted indole derivatives | **IND9** | 392.1136 | 32.67 | 5 | 0 | 3 | 2.9101 |
| 149. | Substituted indole derivatives | **IND13** | 395.0991 | 63.78 | 4 | 1 | 4 | 1.2231 |
| 150. | Substituted indole derivatives | **IND14** | 433.1148 | 54.99 | 6 | 0 | 4 | 1.8987 |
| 151. | Substituted indole derivatives | **IND15** | 389.0886 | 45.76 | 3 | 0 | 3 | 1.9065 |
| 152. | Substituted indole derivatives | **IND16** | 372.0932 | 71.78 | 3 | 1 | 4 | 1.3831 |
| 153. | Substituted indole derivatives | **IND22** | 412.1091 | 68.49 | 6 | 1 | 5 | 2.4914 |
| 154. | Substituted non heterocyclic compounds | **NHC9** | 376.1078 | 64.99 | 6 | 1 | 5 | 1.7155 |
| 155. | Substituted non heterocyclic compounds | **NHC10** | 374.0921 | 53.99 | 4 | 0 | 5 | 1.2309 |
| 156. | Substituted non heterocyclic compounds | **NHC11** | 280.0736 | 52.6 | 3 | 0 | 4 | 0.7028 |
| 157. | Substituted non heterocyclic compounds | **NHC12** | 368.21 | 81.92 | 6 | 3 | 5 | 3.3416 |
| 158. | Substituted non heterocyclic compounds | **NHC13** | 384.0361 | 46.53 | 4 | 1 | 3 | 2.4528 |
| 159. | Substituted non heterocyclic | **NHC14** | 334.1205 | 52.6 | 6 | 0 | 4 | 2.1796 |

| | | | | | | | | |
|---|---|---|---|---|---|---|---|---|
| | compounds | | | | | | | |
| 160. | Substituted non heterocyclic compounds | **NHC15** | 376.2977 | 57.53 | 3 | 2 | 3 | -0.3897 |
| 161. | Substituted non heterocyclic compounds | **NHC16** | 280.0947 | 93.06 | 7 | 2 | 6 | 0.877 |
| 162. | Substituted non heterocyclic compounds | **NHC32** | 476.1074 | 100.71 | 9 | 1 | 6 | 3.7725 |
| 163. | Substituted non Heterocyclic compounds | **NHC23** | 342.0578 | 17.07 | 2 | 0 | 1 | 3.2704 |
| 164. | Substituted non Heterocyclic compounds | **NHC24** | 247.1572 | 40.54 | 5 | 1 | 3 | 1.9692 |
| 165. | Substituted non Heterocyclic compounds | **NHC25** | 360.1508 | 97.64 | 7 | 2 | 5 | 0.901 |
| 166. | Substituted non Heterocyclic compounds | **NHC26** | 316.1423 | 87.66 | 8 | 3 | 6 | 0.4334 |
| 167. | Substituted non Heterocyclic compounds | **NHC27** | 346.1641 | 125.71 | 9 | 5 | 8 | -1.2093 |
| 168. | Substituted non Heterocyclic compounds | **NHC28** | 302.0822 | 50.69 | 5 | 1 | 4 | 2.0631 |
| 169. | Substituted Phenyl linked thiazolidine | **THZD1** | 397.0322 | 126.18 | 4 | 2 | 6 | 0.7116 |

| | | | | | | | | |
|---|---|---|---|---|---|---|---|---|
| 170. | Substituted Phenyl linked thiazolidine | **THZD2** | 381.0617 | 126.18 | 4 | 2 | 6 | 0.2527 |
| 171. | Substituted Phenyl linked thiazolidine | **THZD3** | 388.0285 | 109.8 | 6 | 2 | 6 | 1.4673 |
| 172. | Tetrazole and Triazole derivatives | **TTZ1** | 344.1055 | 112.1 | 4 | 1 | 8 | 0.2709 |
| 173. | Tetrazole and Triazole derivatives | **TTZ2** | 342.0409 | 90.92 | 4 | 0 | 4 | 2.5316 |
| 174. | Tetrazole and Triazole derivatives | **TTZ3** | 362.0604 | 96.5 | 5 | 1 | 6 | 1.0777 |
| 175. | Tetrazole and Triazole derivatives | **TTZ5** | 453.0662 | 125.6 | 6 | 2 | 8 | 0.3835 |
| 176. | Tetrazole and Triazole derivatives | **TTZ6** | 398.159 | 93.98 | 8 | 1 | 9 | -1.4506 |
| 177. | Tetrazole and Triazole derivatives | **TTZ7** | 399.1543 | 120 | 8 | 2 | 10 | -2.1484 |
| 178. | Tetrazole and Triazole derivatives | **TTZ9** | 343.1103 | 86.08 | 4 | 0 | 7 | 0.8845 |
| 179. | Tetrazole and Triazole derivatives | **TTZ10** | 378.0553 | 92.07 | 6 | 0 | 7 | 1.2801 |
| 180. | Triazole linked pyrazole | **PYZ49** | 397.1009 | 86.08 | 4 | 0 | 7 | 1.0019 |
| 181. | Triazole linked pyrazole | **PYZ50** | 394.1212 | 112.1 | 4 | 1 | 8 | -0.1046 |
| 182. | Substituted quinoline derivatives | **QIN (2)** | 403.0878 | 92.18 | 4 | 1 | 5 | 2.1381 |
| 183. | Substituted quinoline derivatives | **QIN (3)** | 382.0833 | 95.31 | 5 | 3 | 5 | 0.6951 |
| 184. | Substituted quinoline derivatives | **QIN (4)** | 397.1193 | 65.37 | 4 | 1 | 4 | 2.3135 |
| 185. | Substituted quinoline derivatives | **QIN (6)** | 406.1084 | 58.97 | 5 | 0 | 5 | 1.6455 |
| 186. | Substituted quinoline derivatives | **QIN (7)** | 440.0694 | 58.97 | 5 | 0 | 5 | 2.0142 |
